# Supplementary material for: Nutrient sensing receptor GPRC6A regulates mTORC1 signaling and Tau biology
Source: Neurobiol Dis. Author manuscript; Available in PMC 2026 May 28. (PMC13218200; doi:10.1016/j.nbd.2025.107054)
Supplement: 1 [file NIHMS2120879-supplement-1.pdf]

Figure 2 Raw Blots

Tau-5 exp 7min

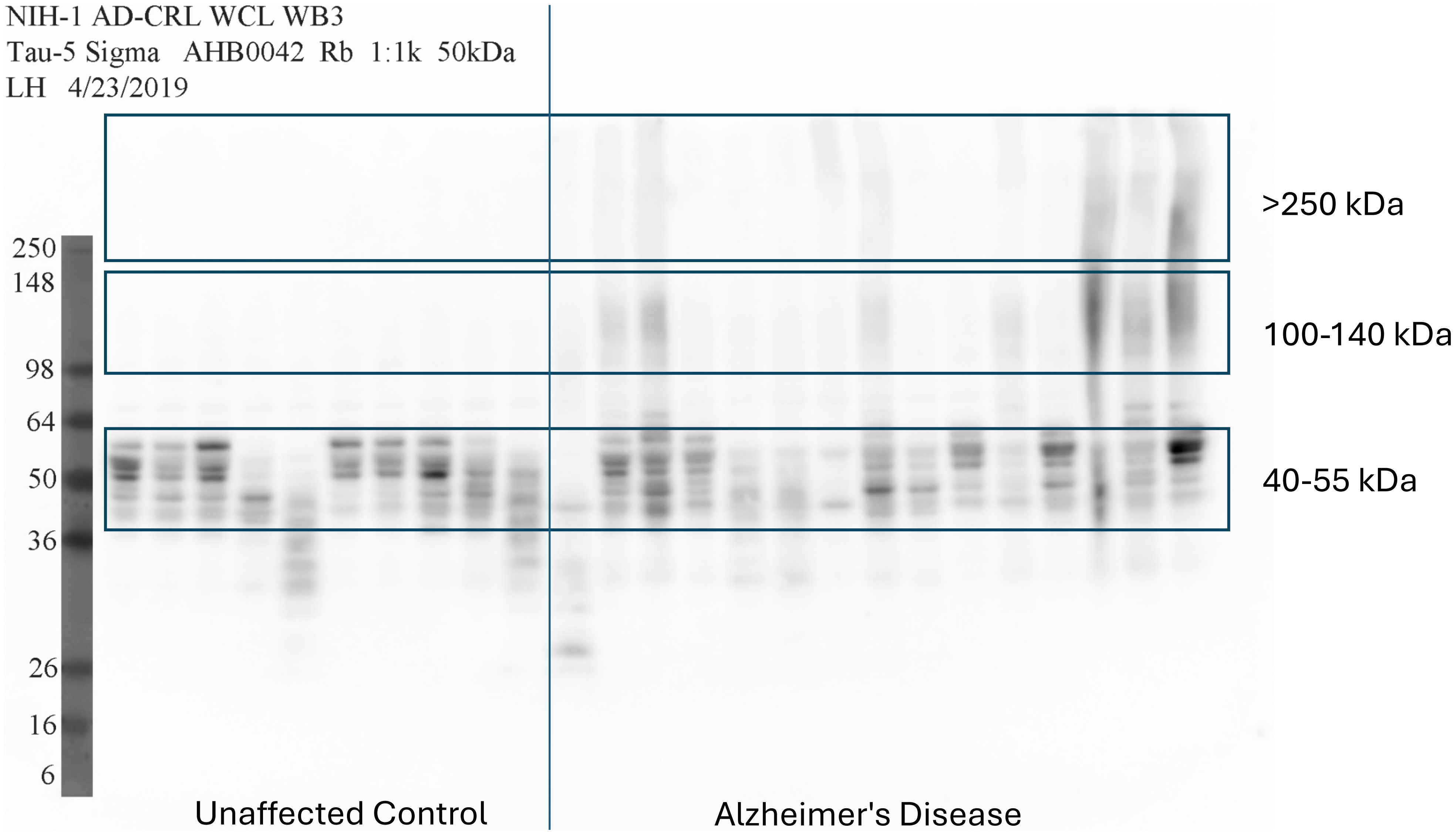

Tau-5, Fig 2 A

Tau-AT8 exp 10min

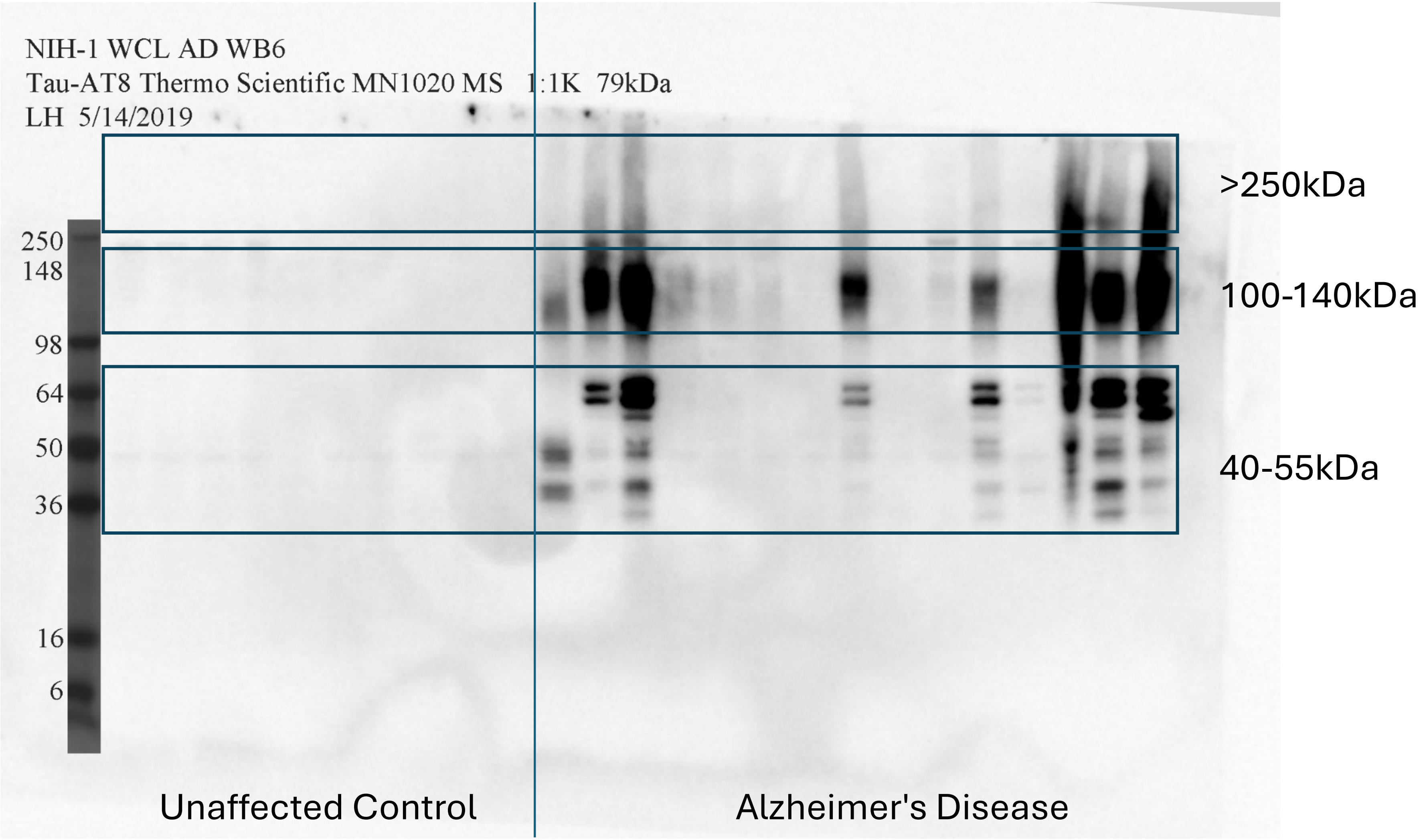

AT-8, Fig 2 A

NIH-AD-WCL-WB5: reprobe with Tau pS214 (abcam)

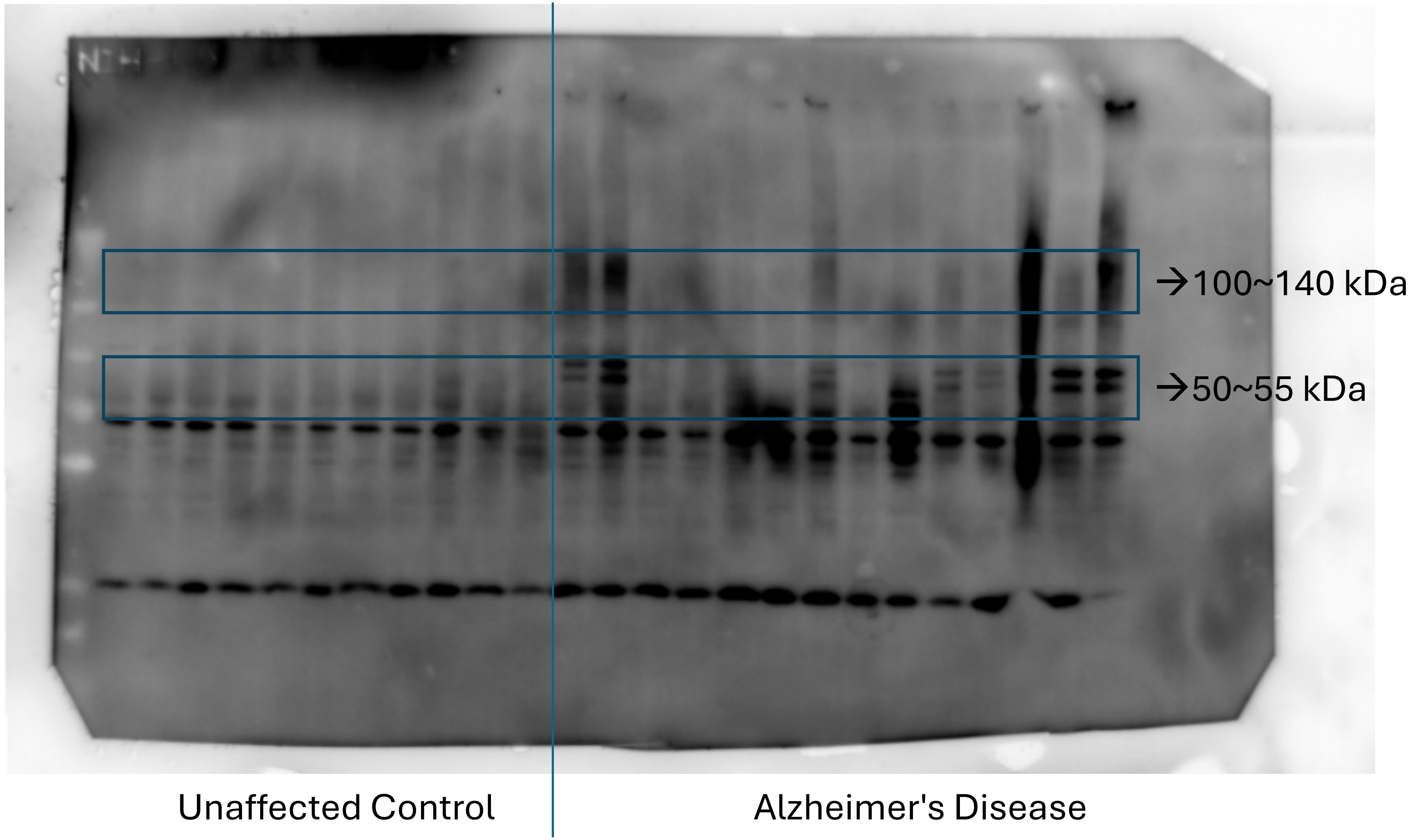

Tau pS214, Fig 2 A

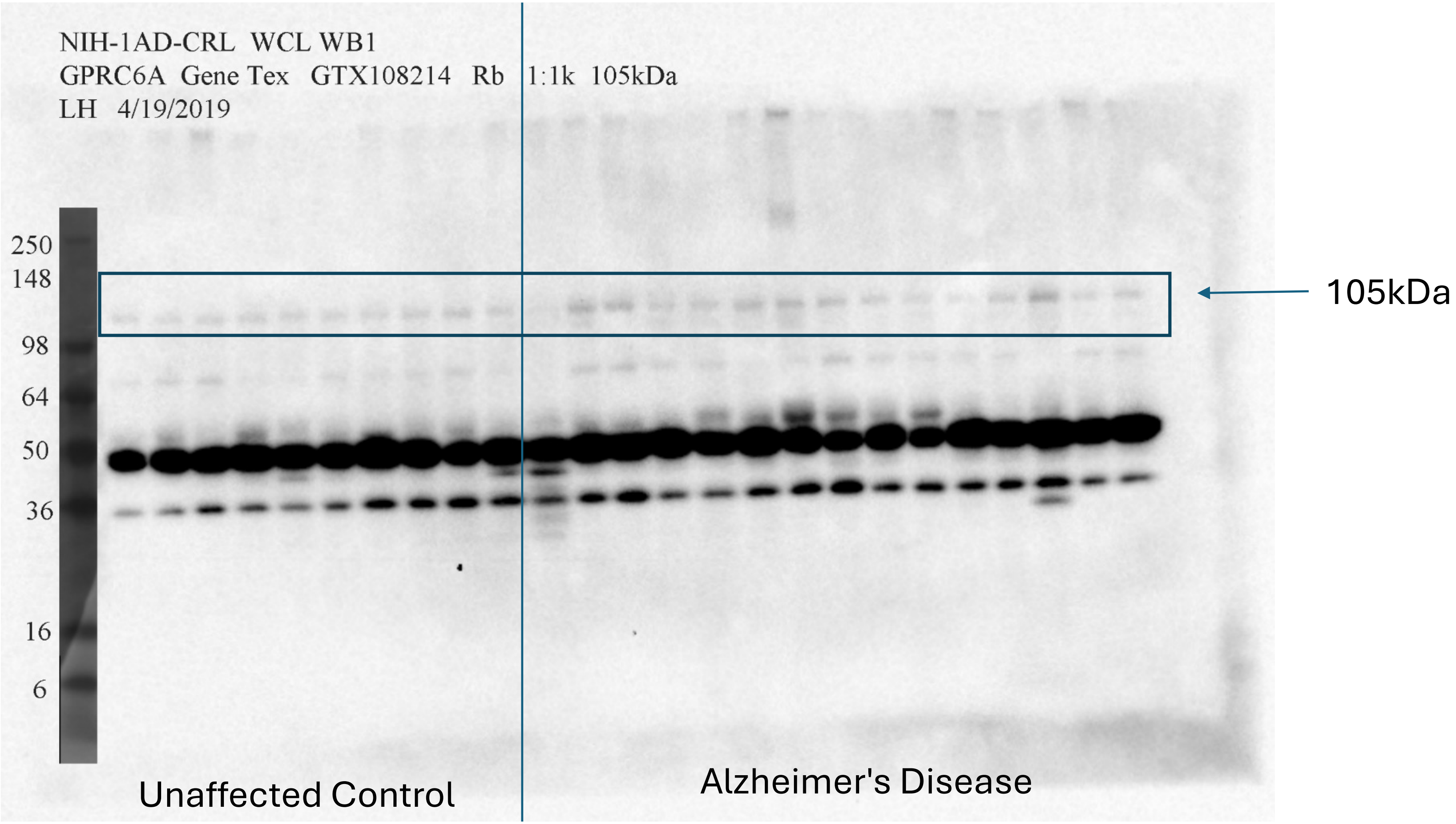

GPRC6A, Fig 2 A

Figure 3 Raw Blots

AK_MTA_WB4 Tau-H150 30s RAW

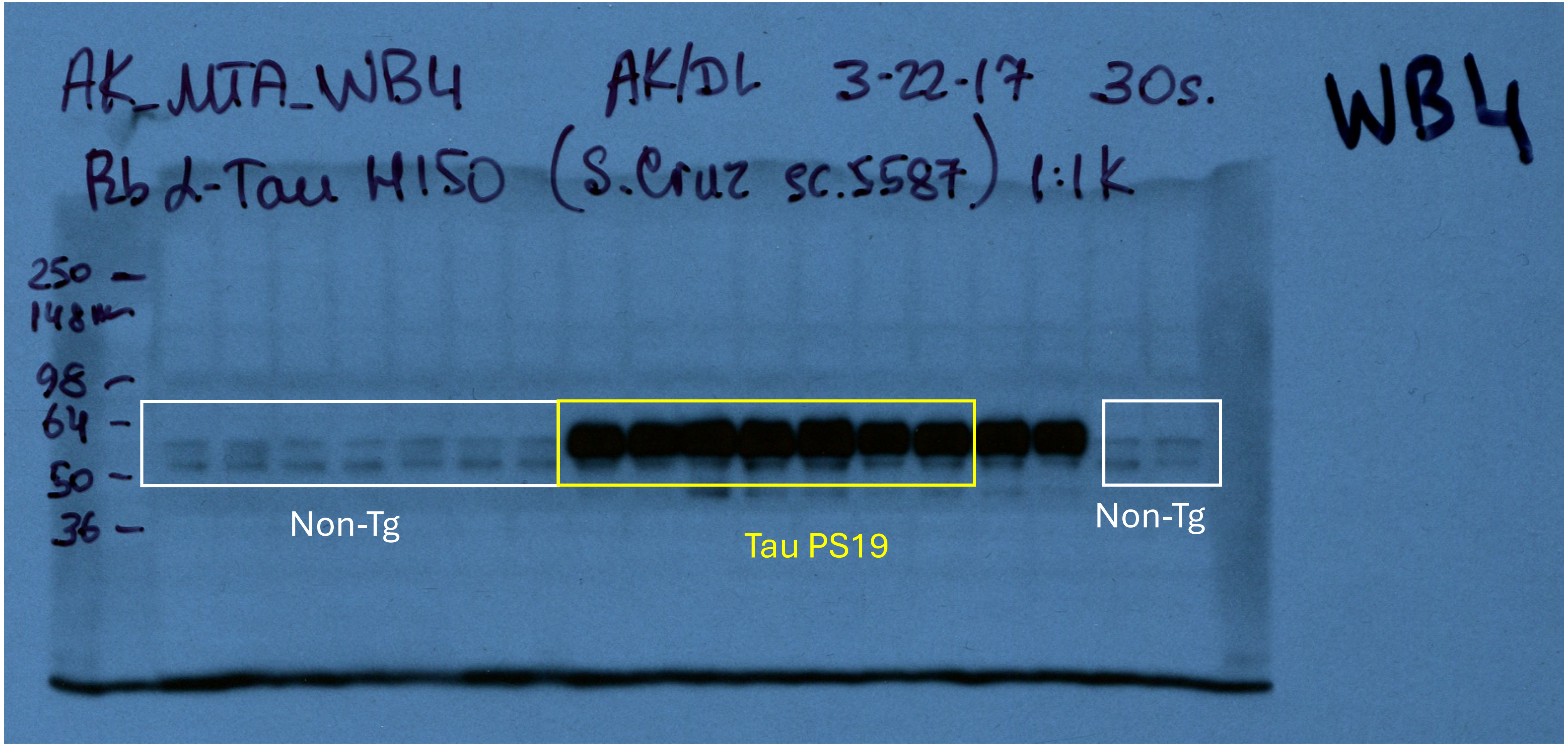

Tau (H150), Fig 3 A

AK-MTA-WB1-Tau pS199-202 exp 5s- RAW

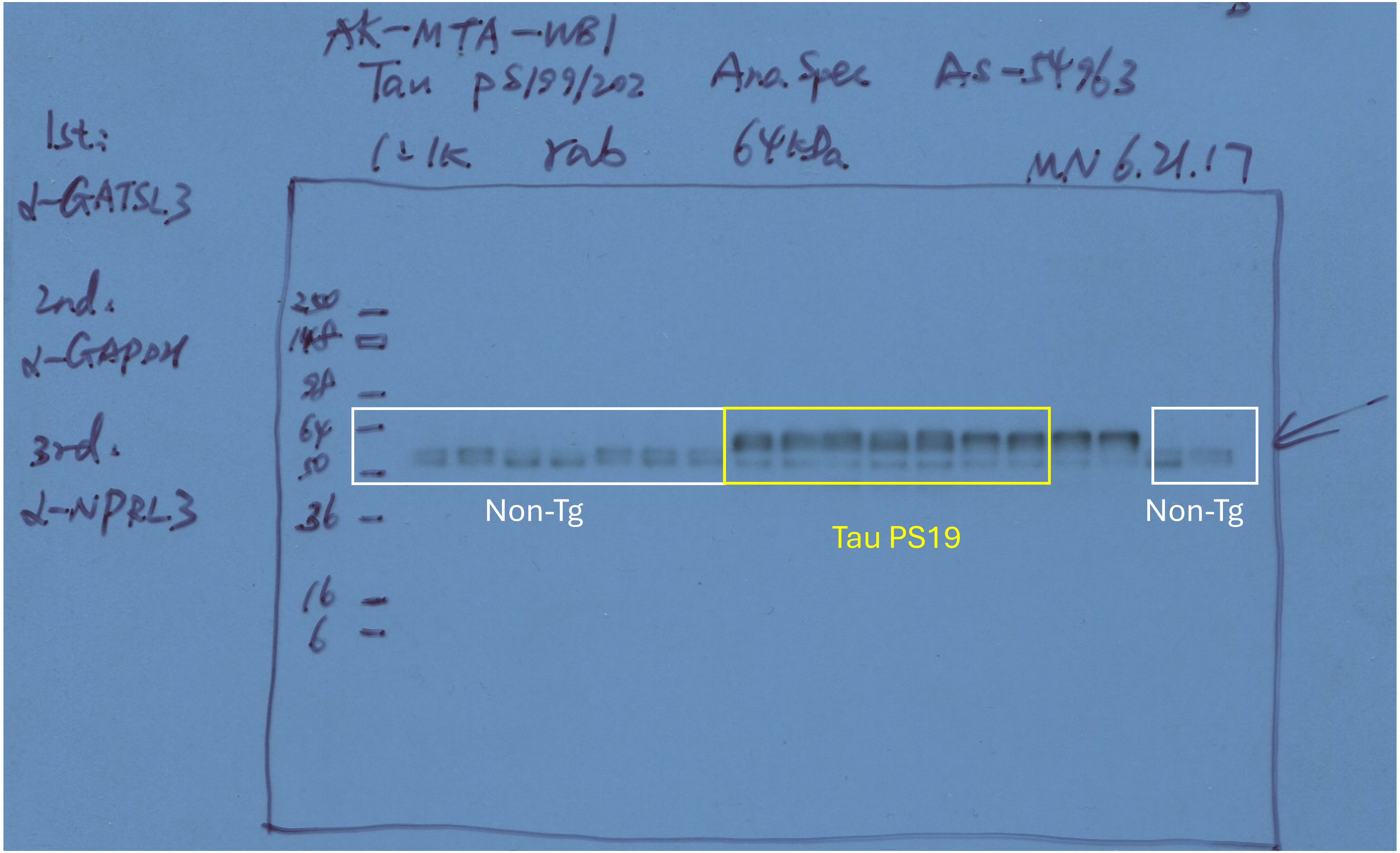

Tau (pS199/202), Fig 3 A

AK-MTA-WB17-AT8 exp 3min RAW

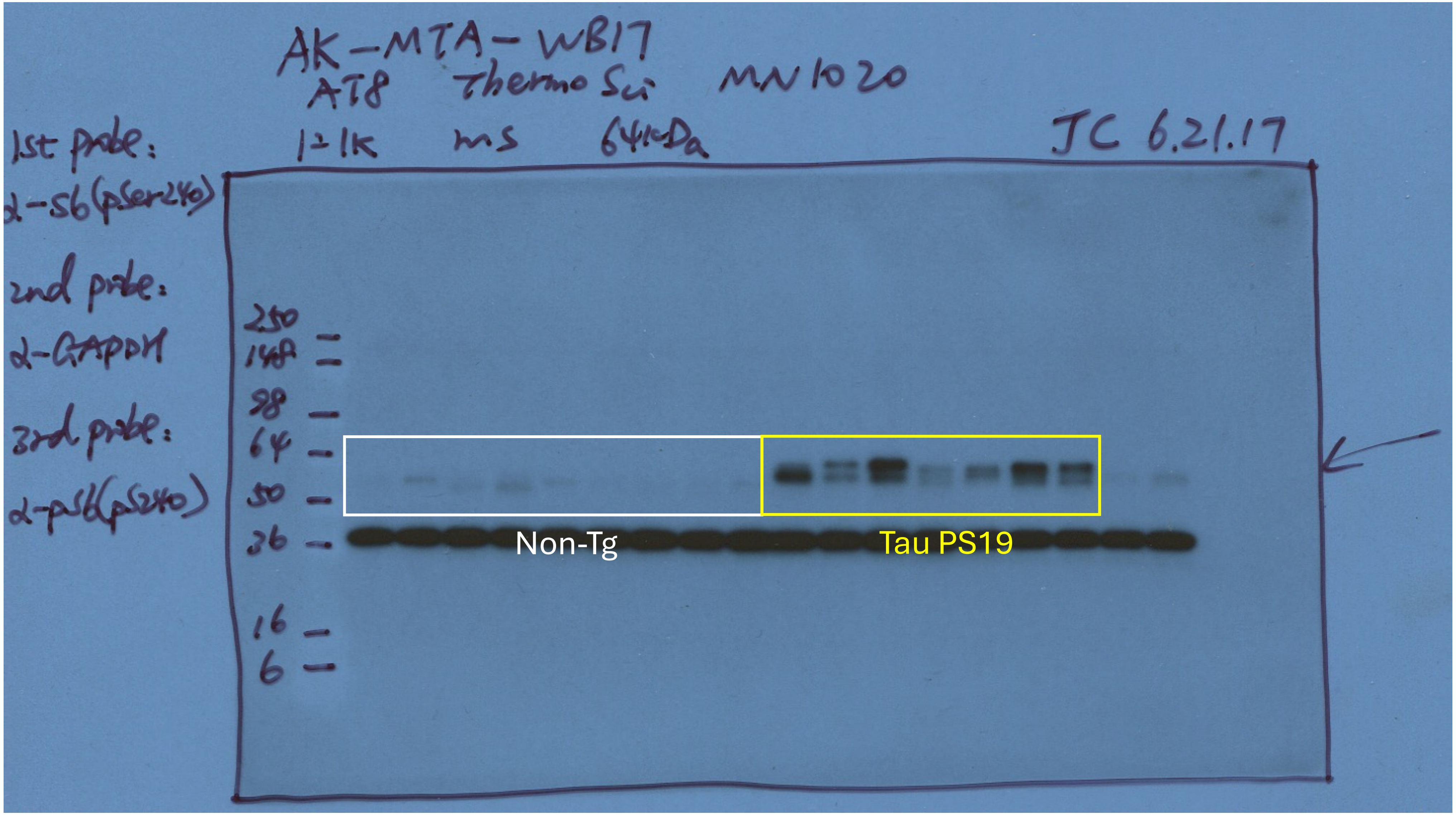

Tau (AT8), Fig 3 A

AK-MTA-WB18-Tau pS396 exp 5s RAW

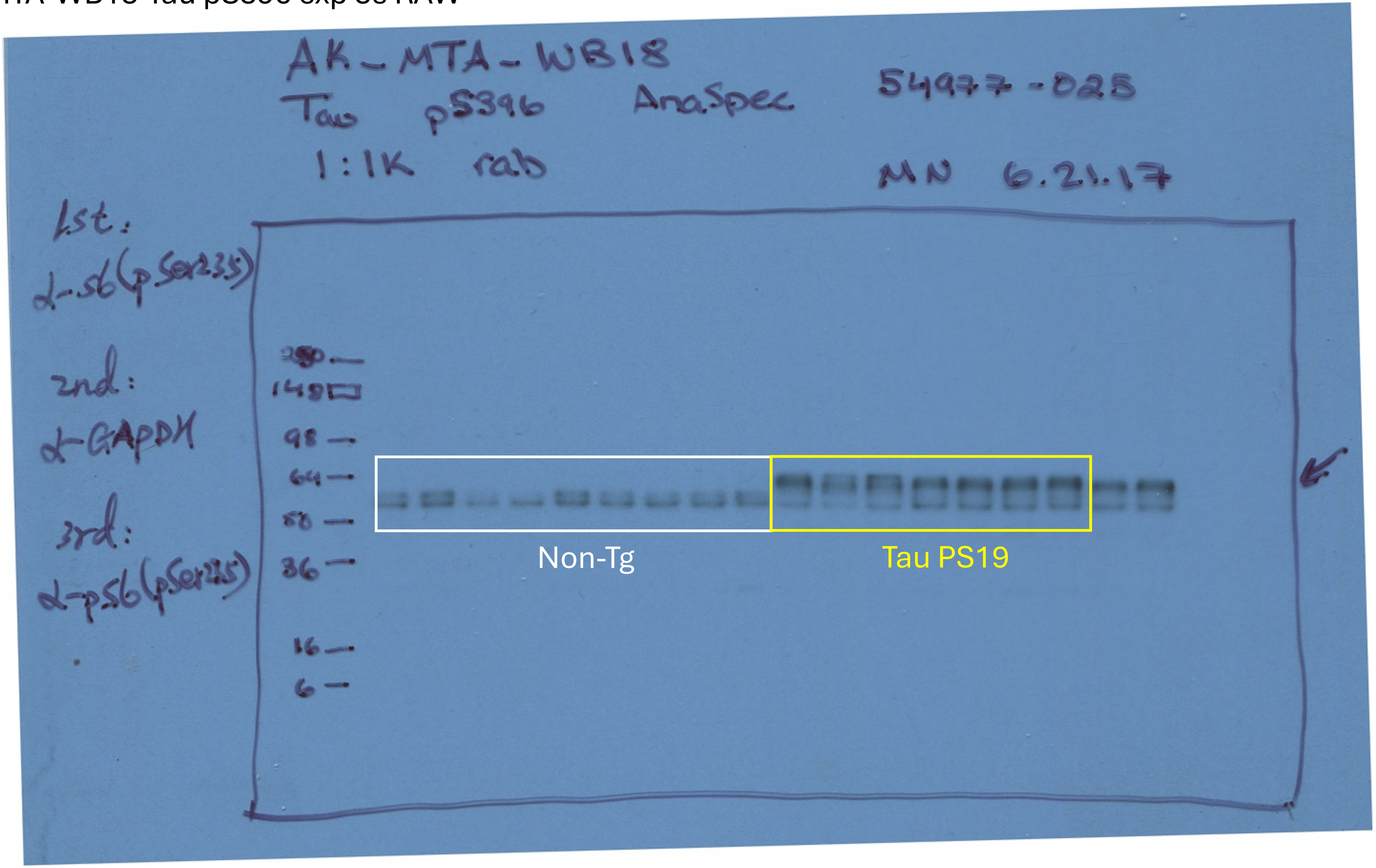

Tau (pS396), Fig 3 A

S1F10-0719-122158_pub

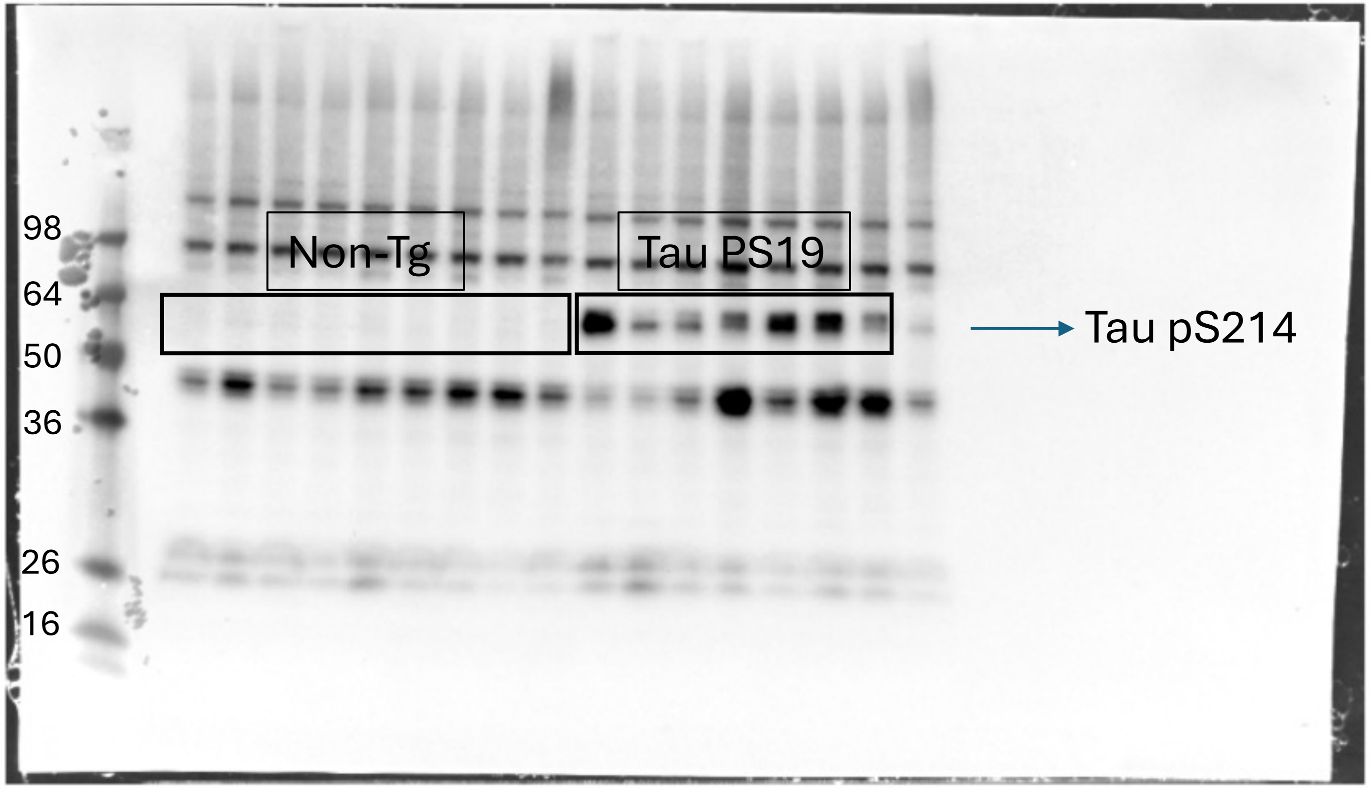

Tau (pS214), Fig 3 A

MTA_WB13_ASS1 reprobe

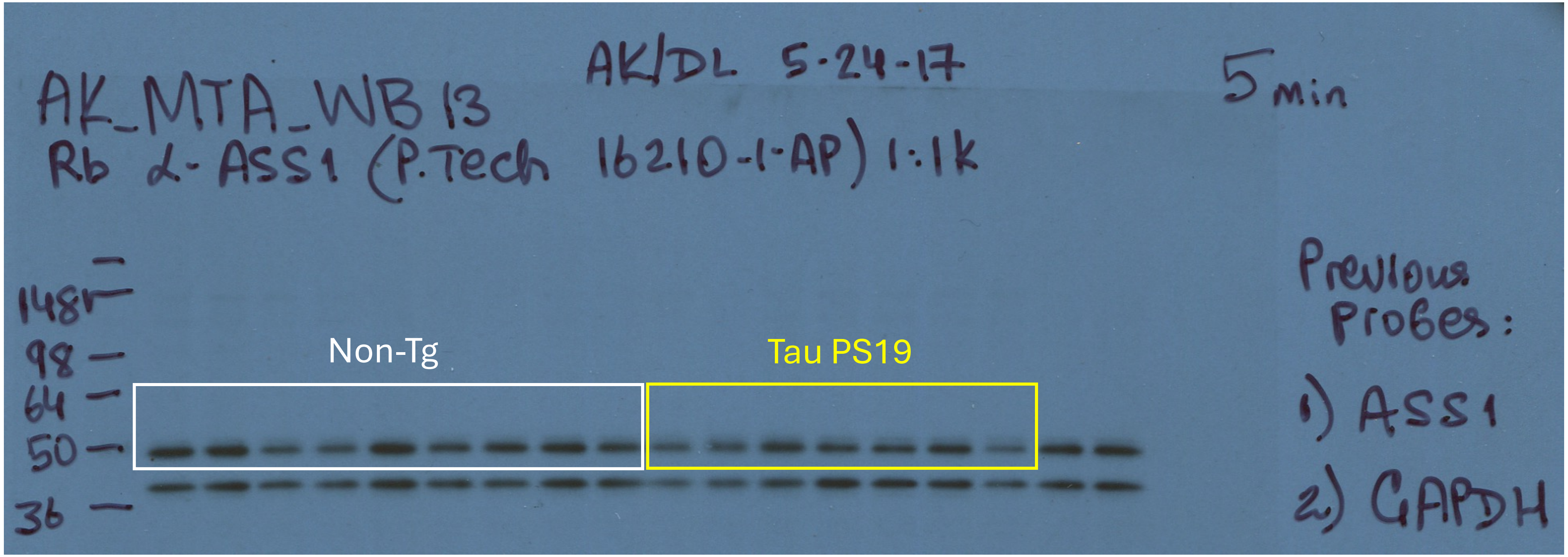

ASS1, Fig 3 C

MTA-WB14 ASL 1min RAW

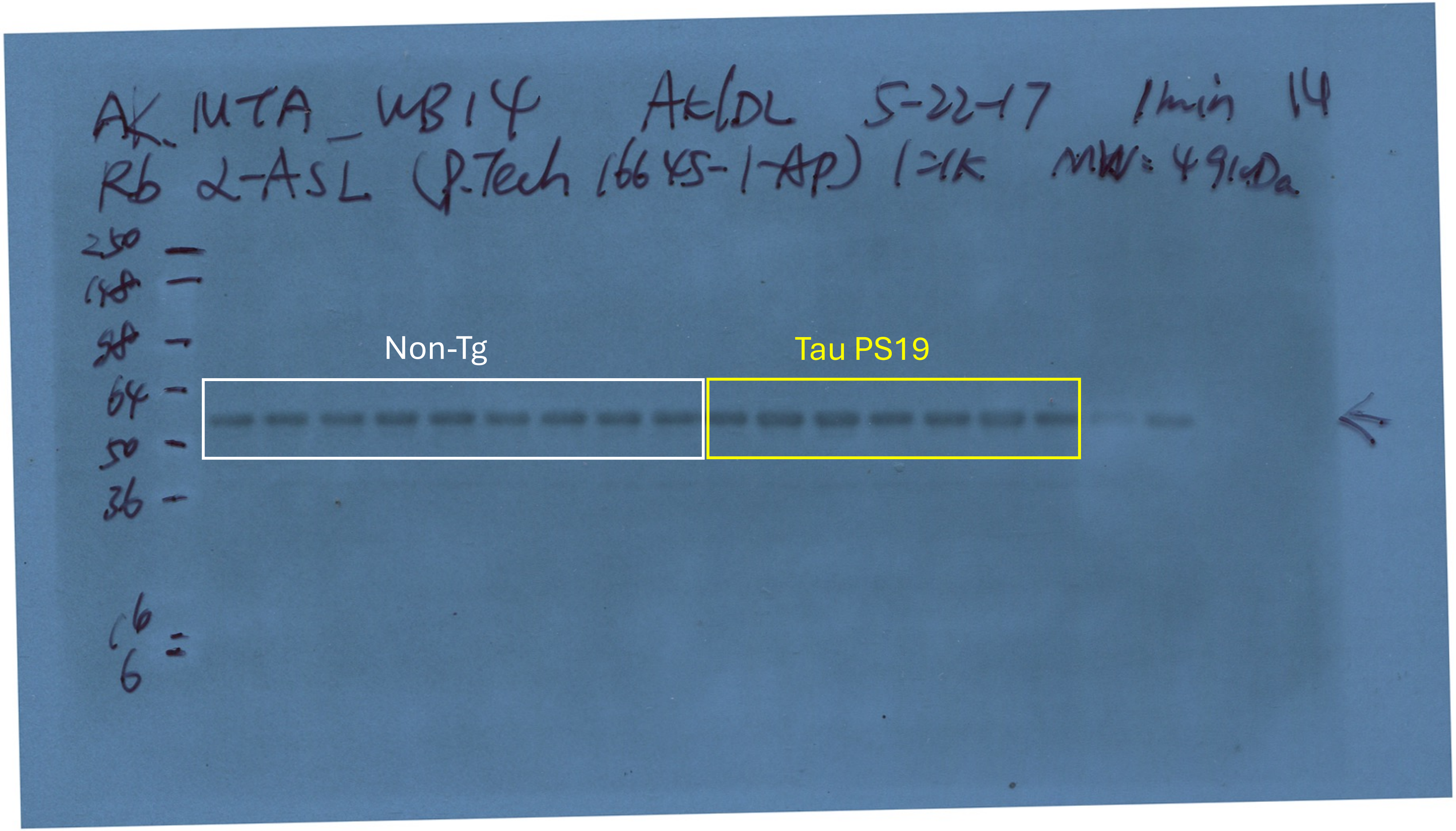

ASL, Fig 3 C

MTA-WB20-GPRC6A exp 1min RAW

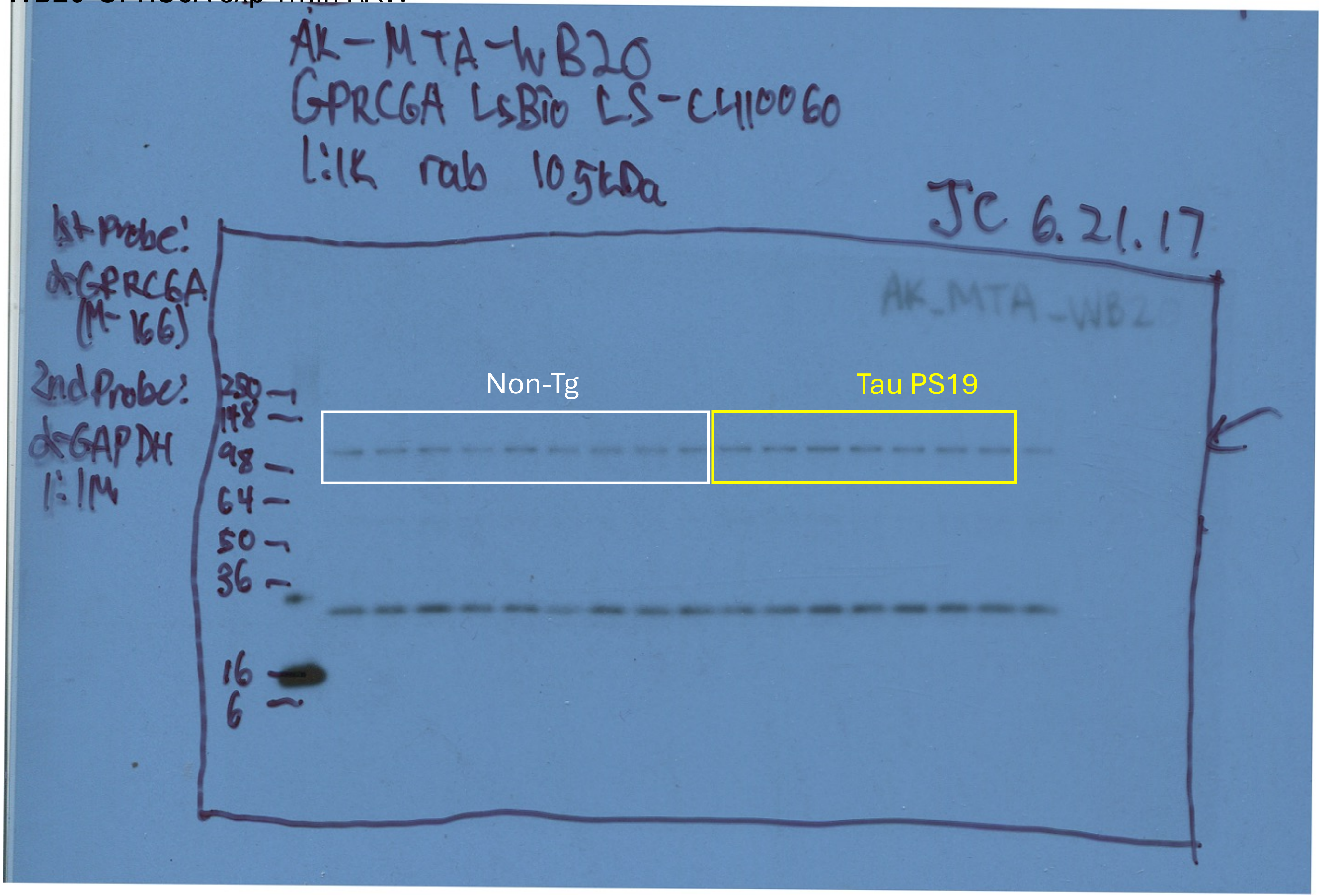

GPRC6A, Fig 3 C

AKMCMTA-WCL-WB4b
S1F10-0719-121536_pub

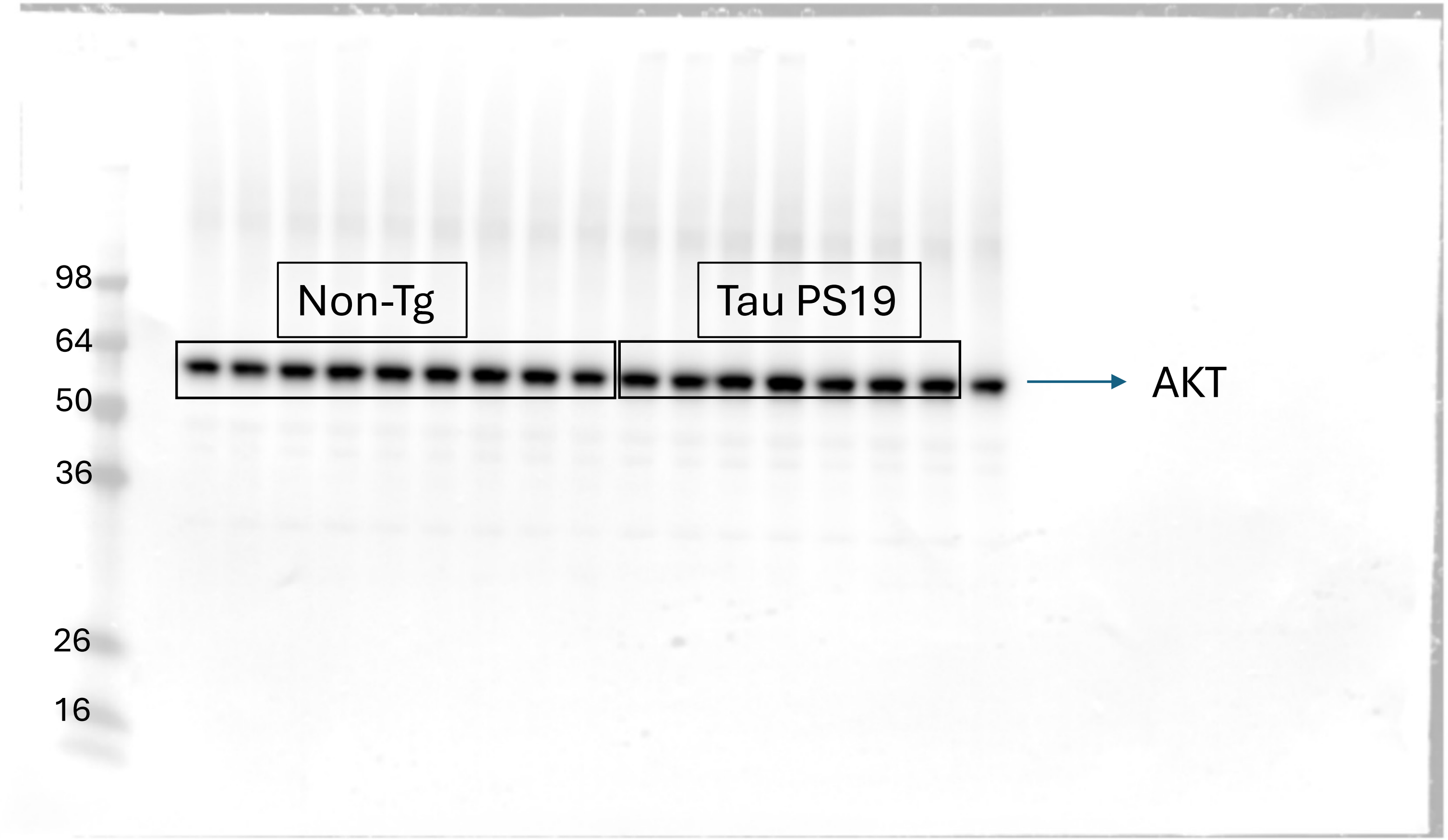

AKT, Fig 3 C

AKMCMTA-WCL-WB4a
S2F5-0718-121802_pub

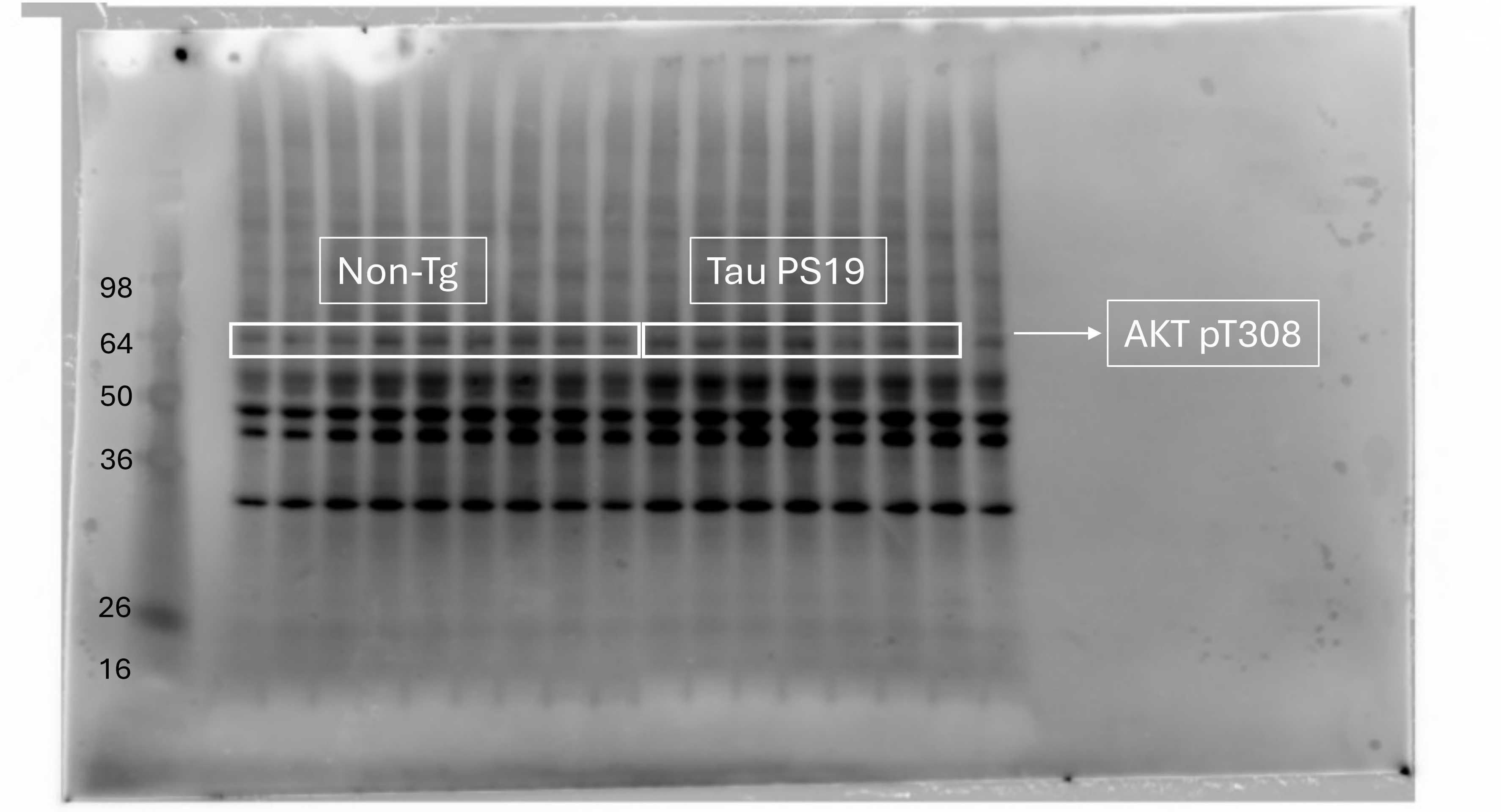

AKT (pT308), Fig 3 C

AKMCMTA-WCL-WB4c
S2F10-0720-161412_pub

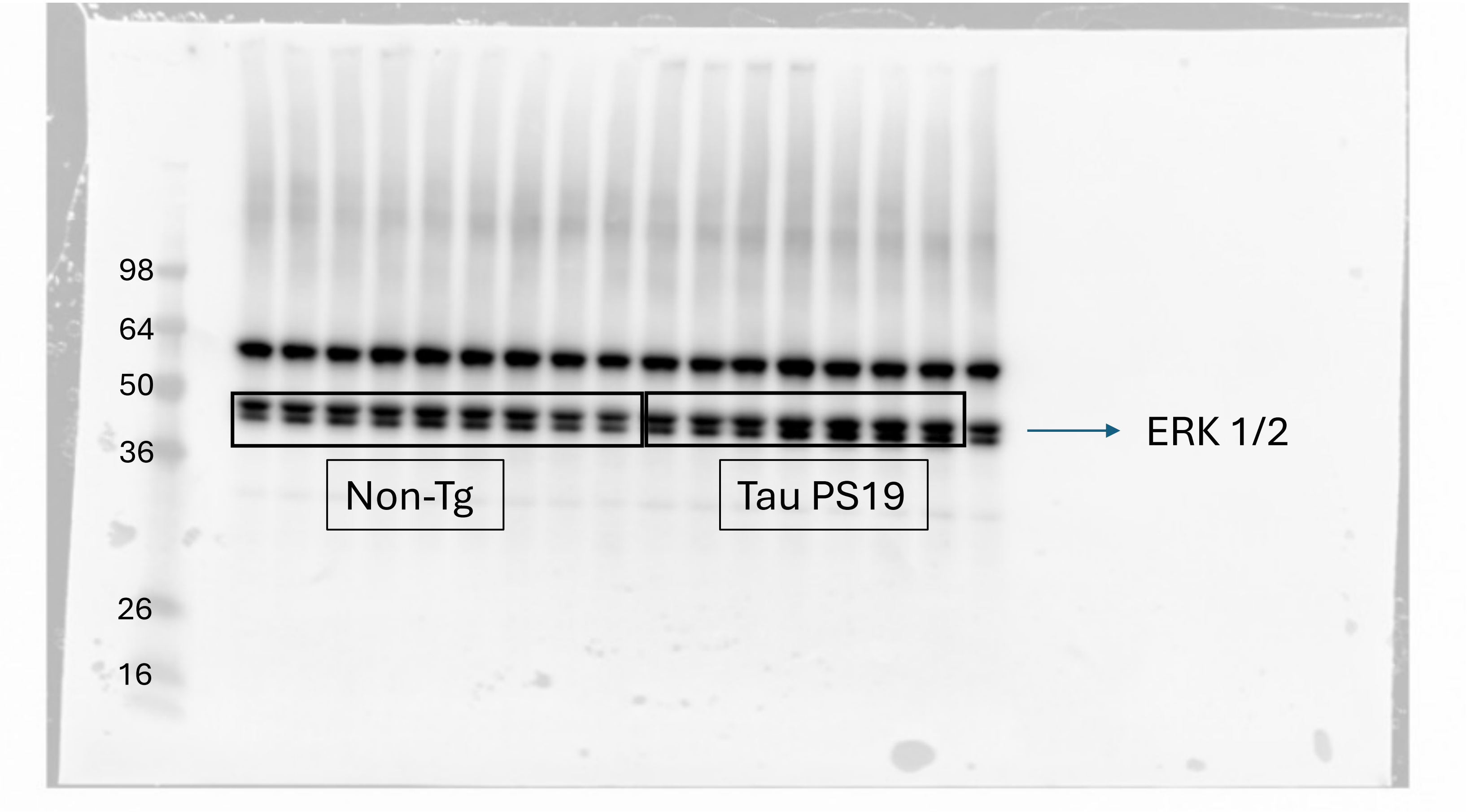

ERK, Fig 3 C

AKMCMTA-WCL-WB5
S1F10-0720-145037_pub

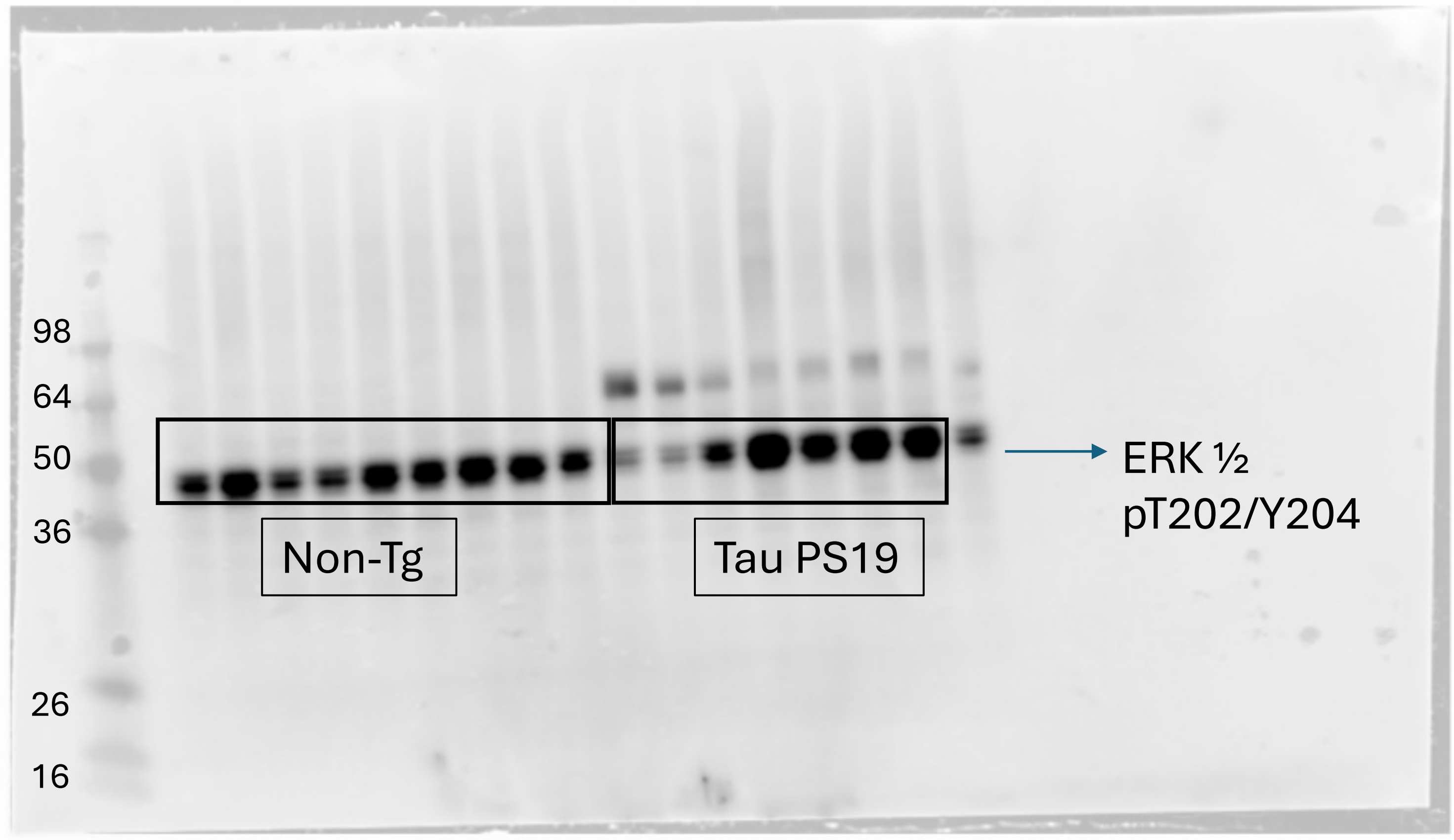

ERK, Fig 3 C

Figure 4 Raw Blots

S2F10-0720-161412_pub

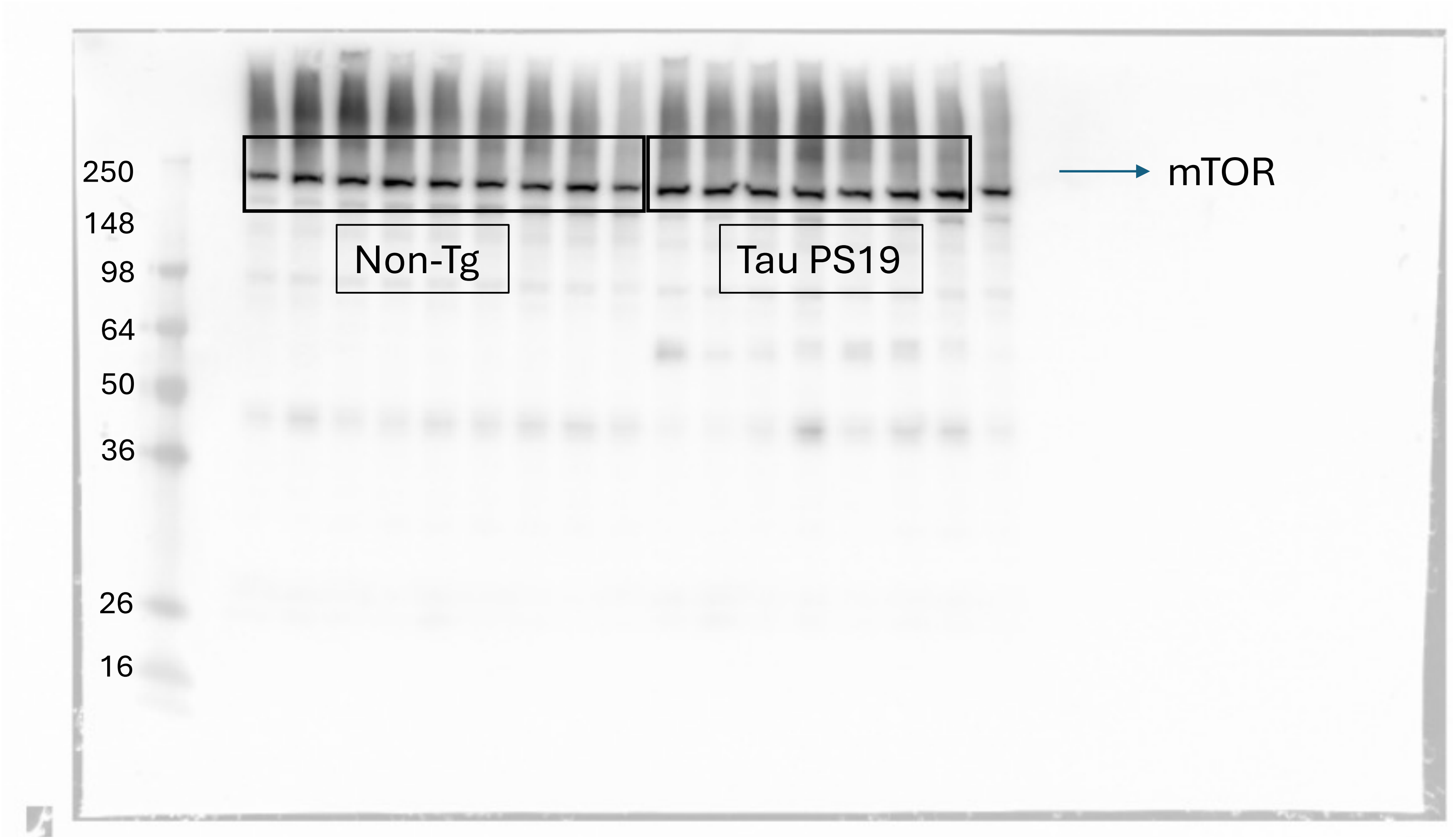

mTOR, Fig 4 A

S1F10-0715-141841_pub

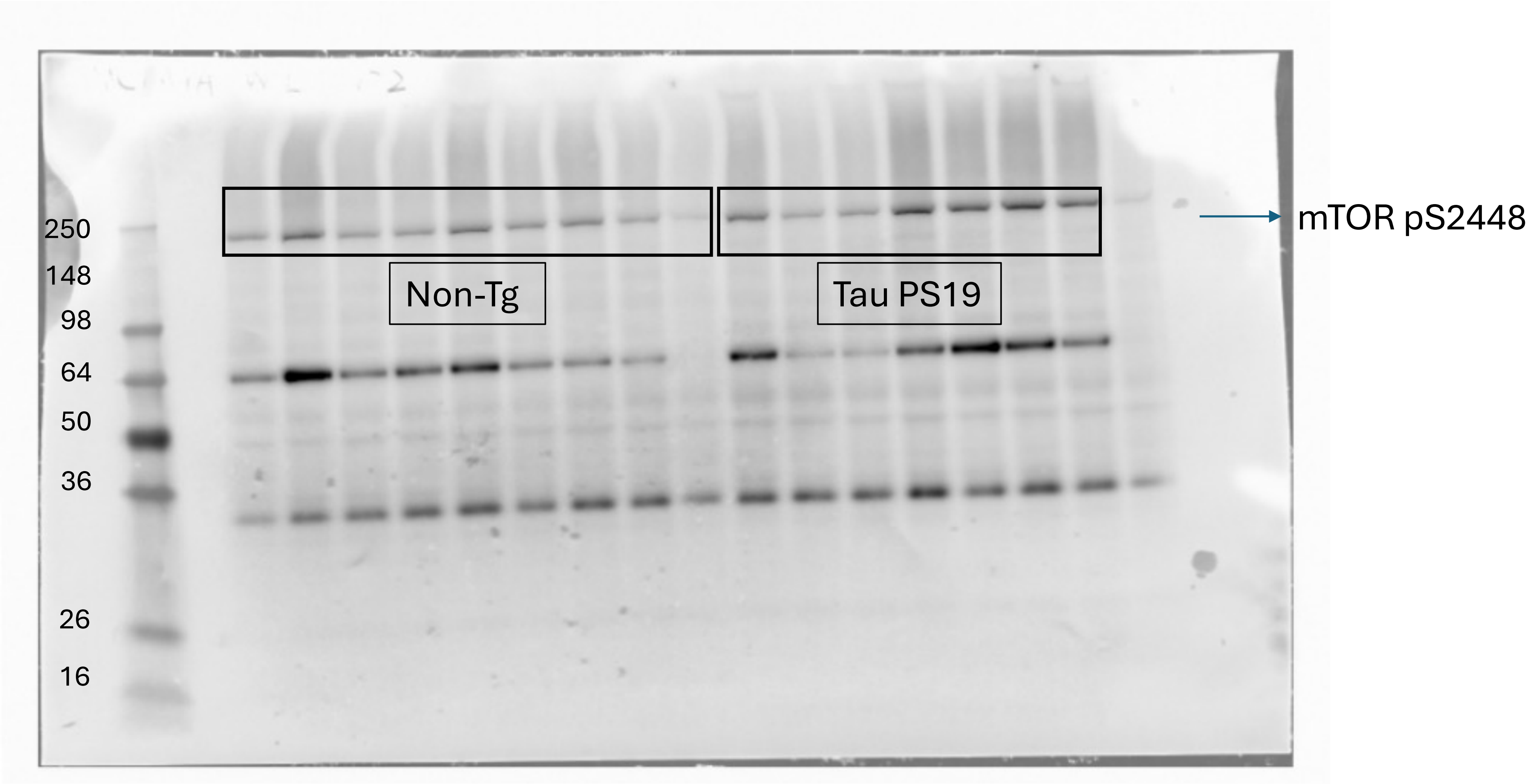

mTOR pS2448, Fig 4 A

AK/JH MTA Study WB10

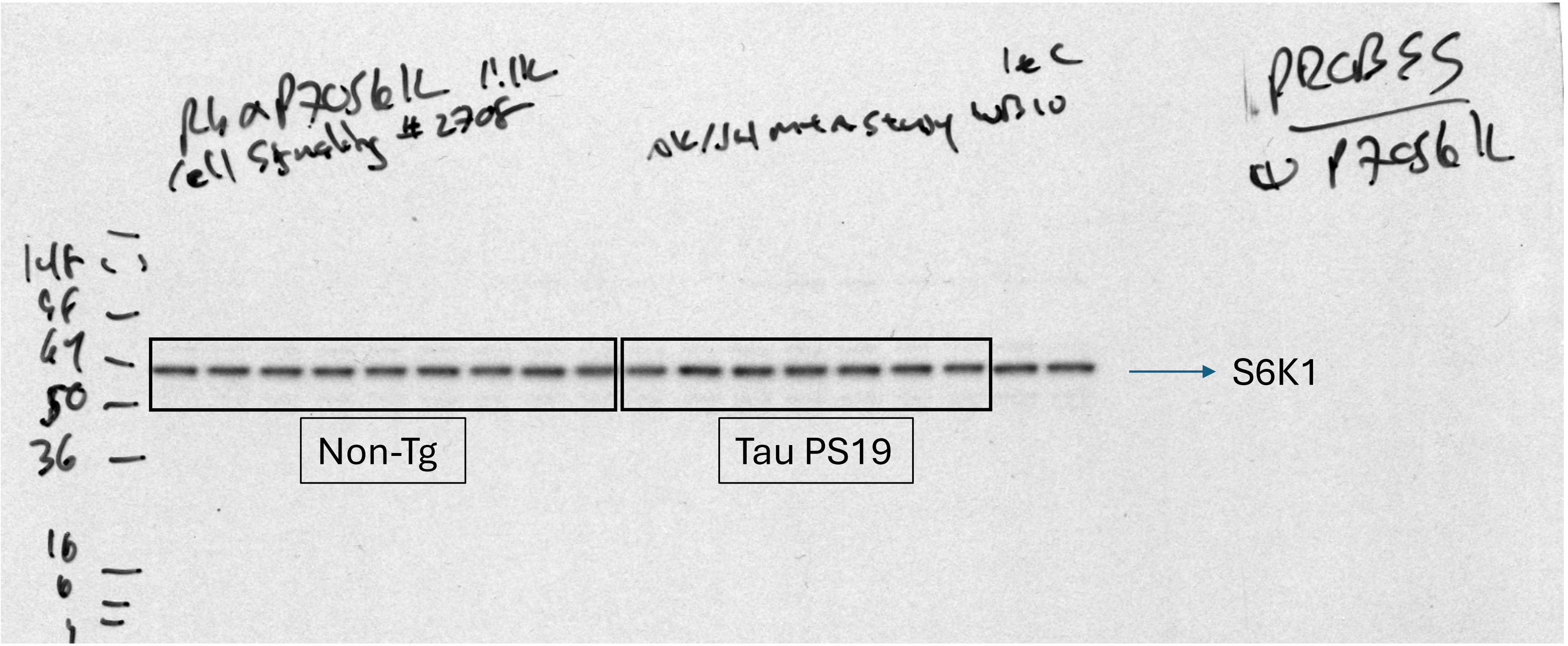

S6K1, Fig 4 A

S2F5-0712-135852_pub

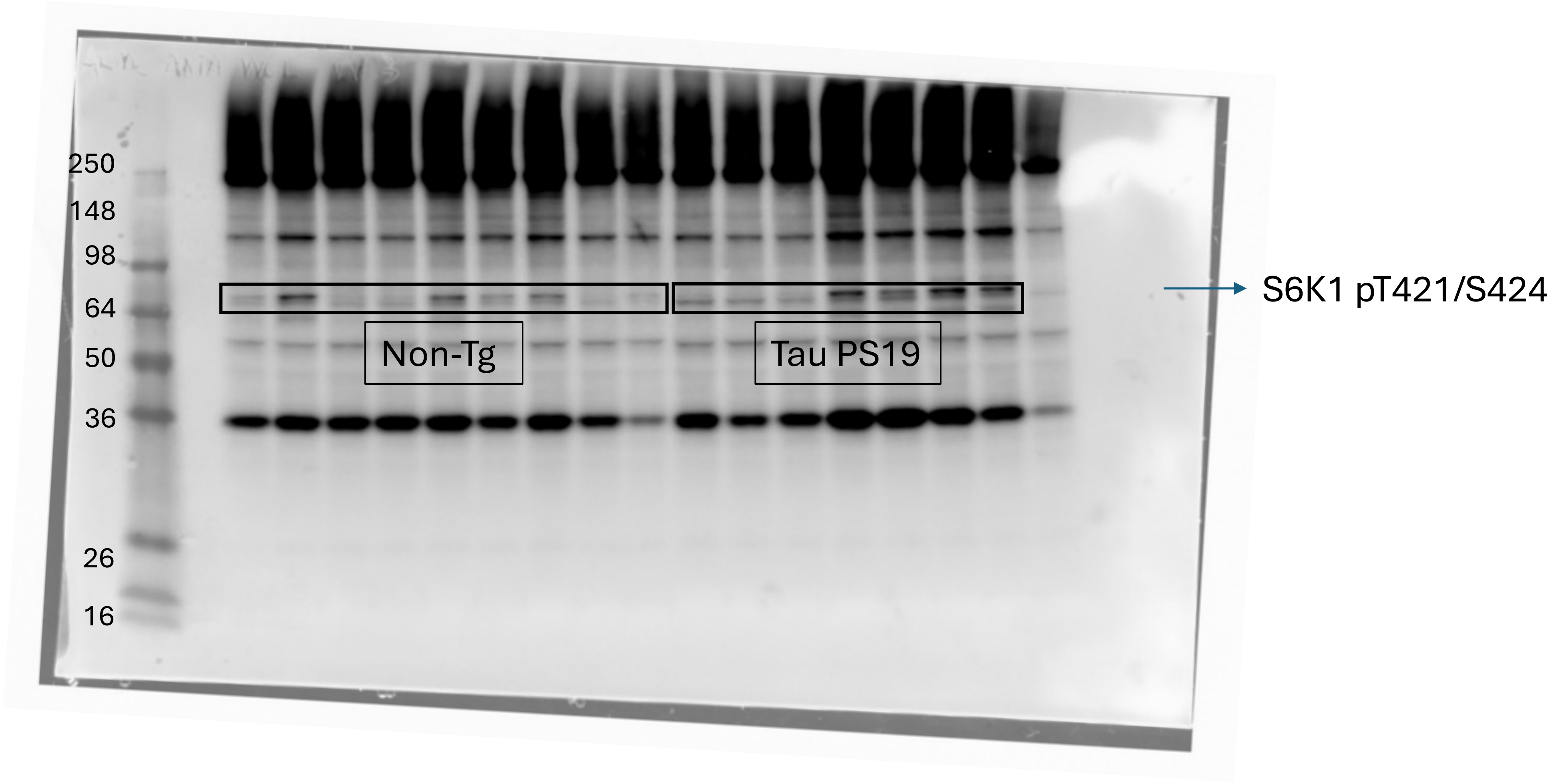

S6K1 pT421/S424, Fig 4 A

AK/JH MTA Study WB11

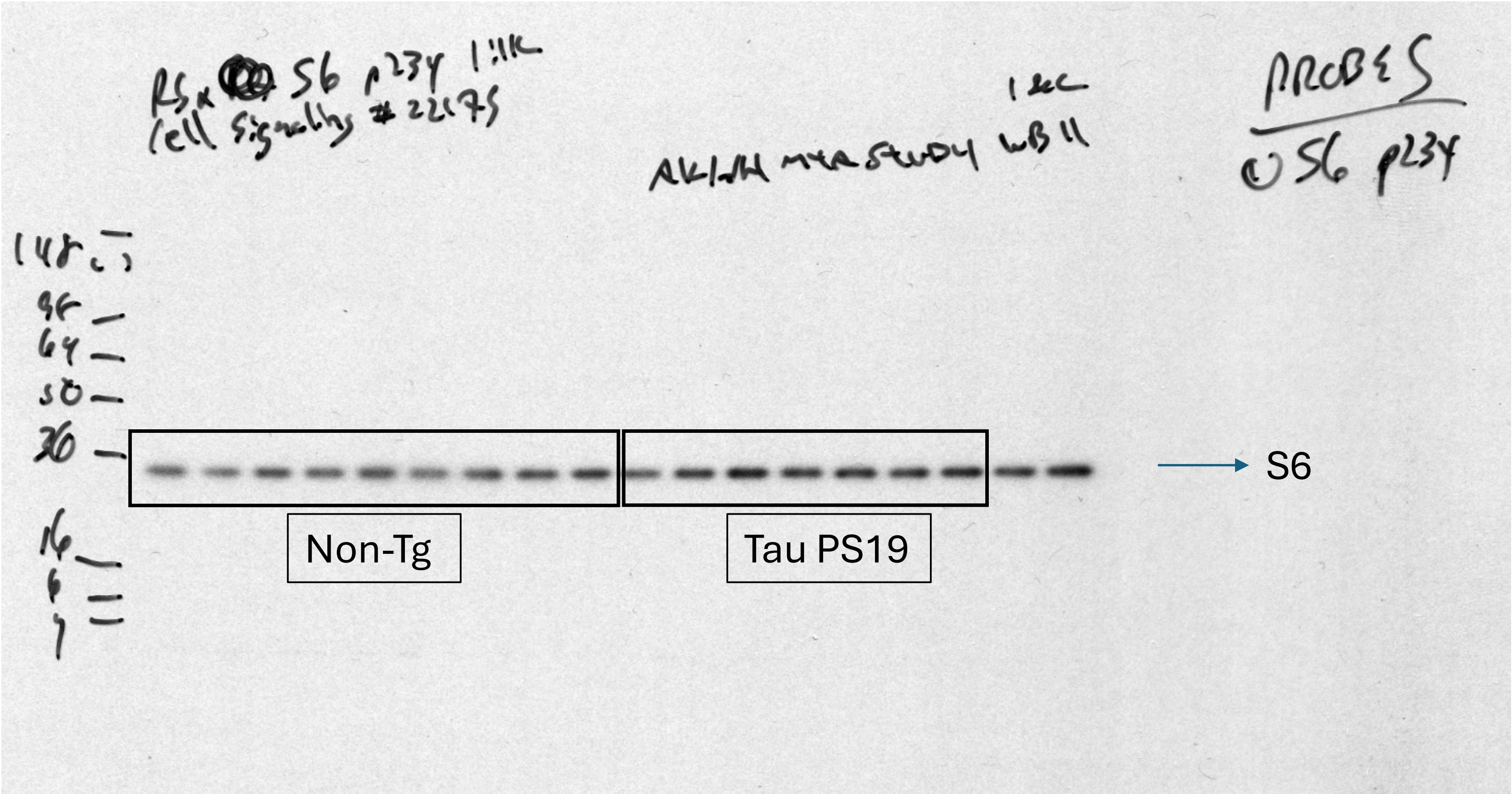

S6, Fig 4 A

7-11-19_WB2 reECL: S1F10-0711-124611_pub

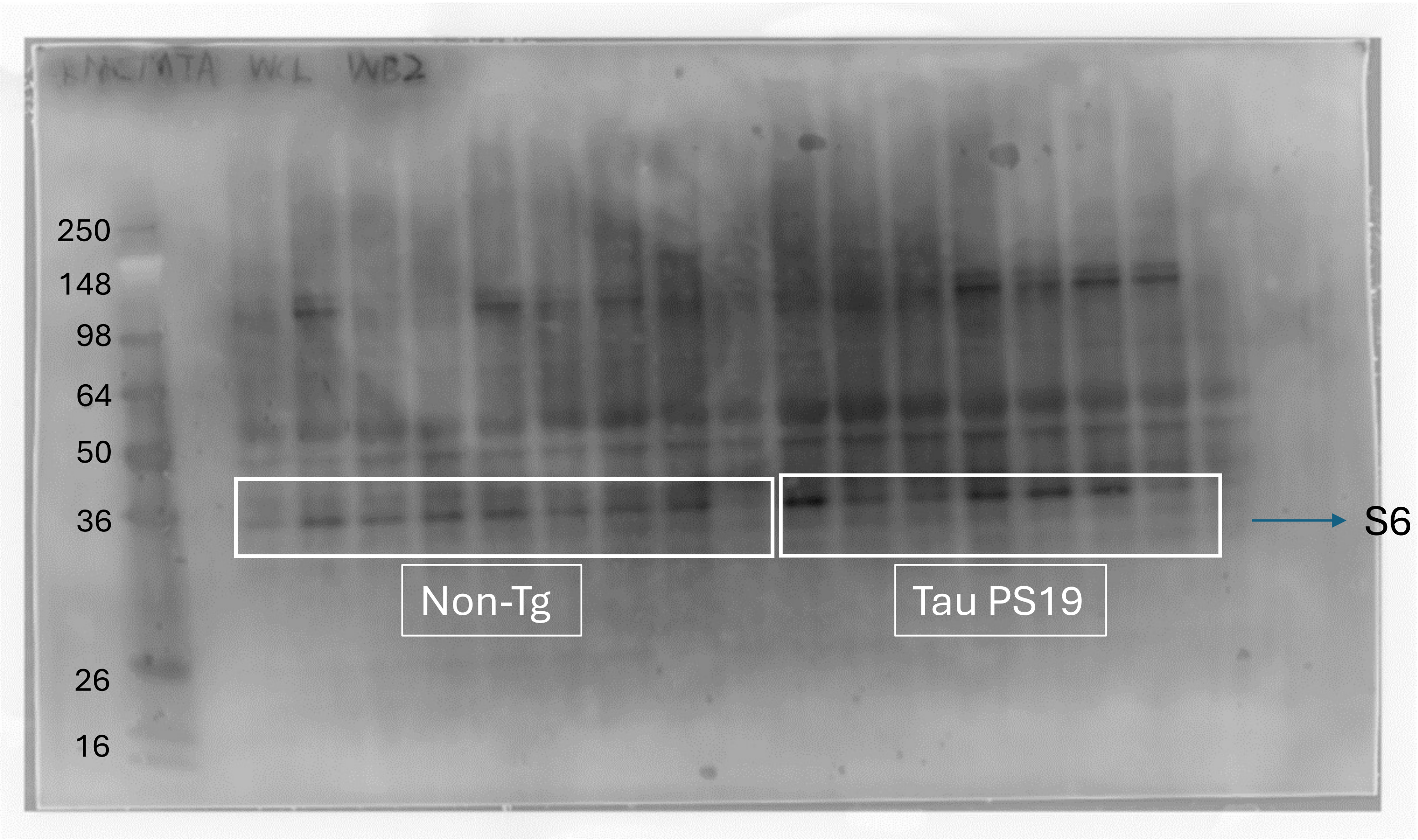

S6 pS235/236, Fig 4 A

AK_MTA_WB5

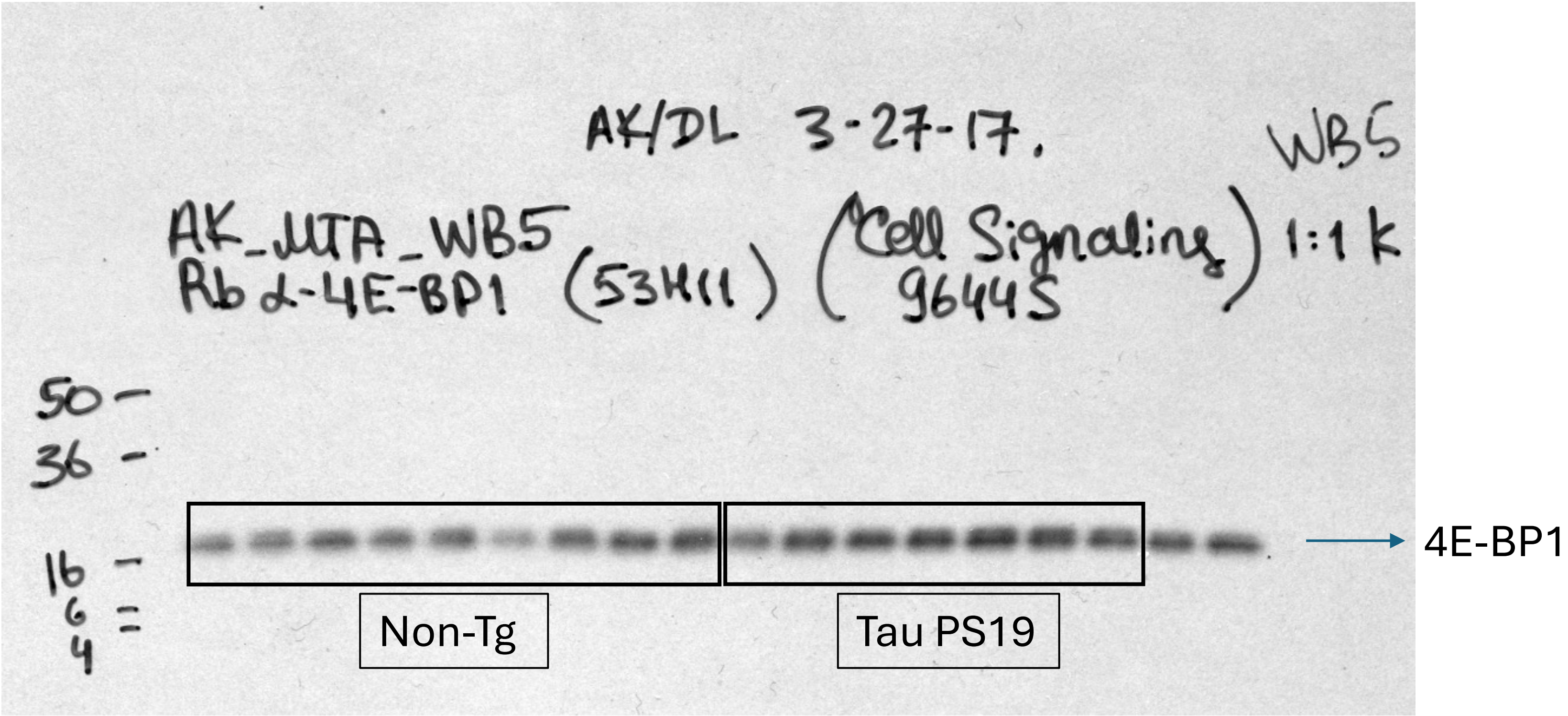

4E-BP1, Fig 4 A

AK_MTA_WB6

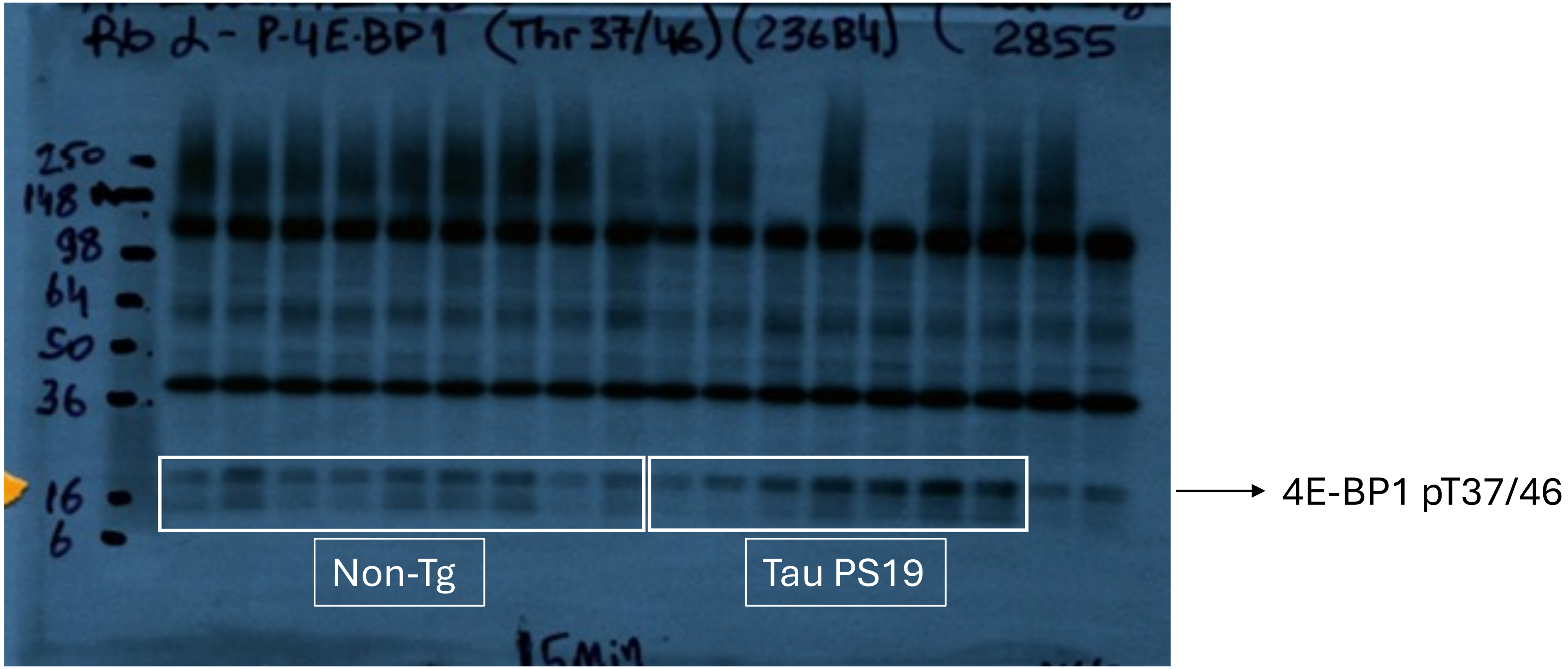

4E-BP1 pT37/46, Fig 4 A

AK_MTA_WB7

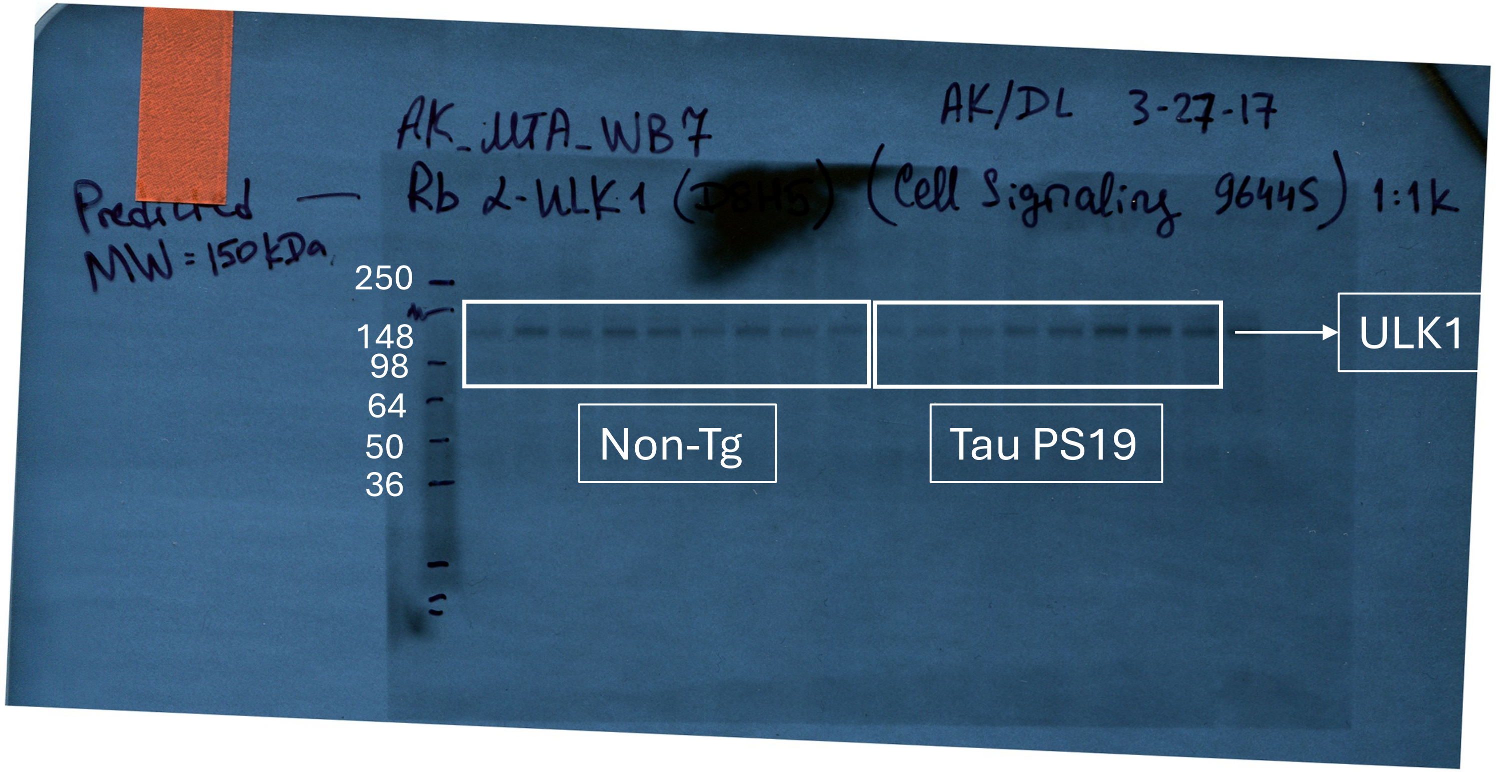

ULK1, Fig 4 F

AK_MTA_WB6

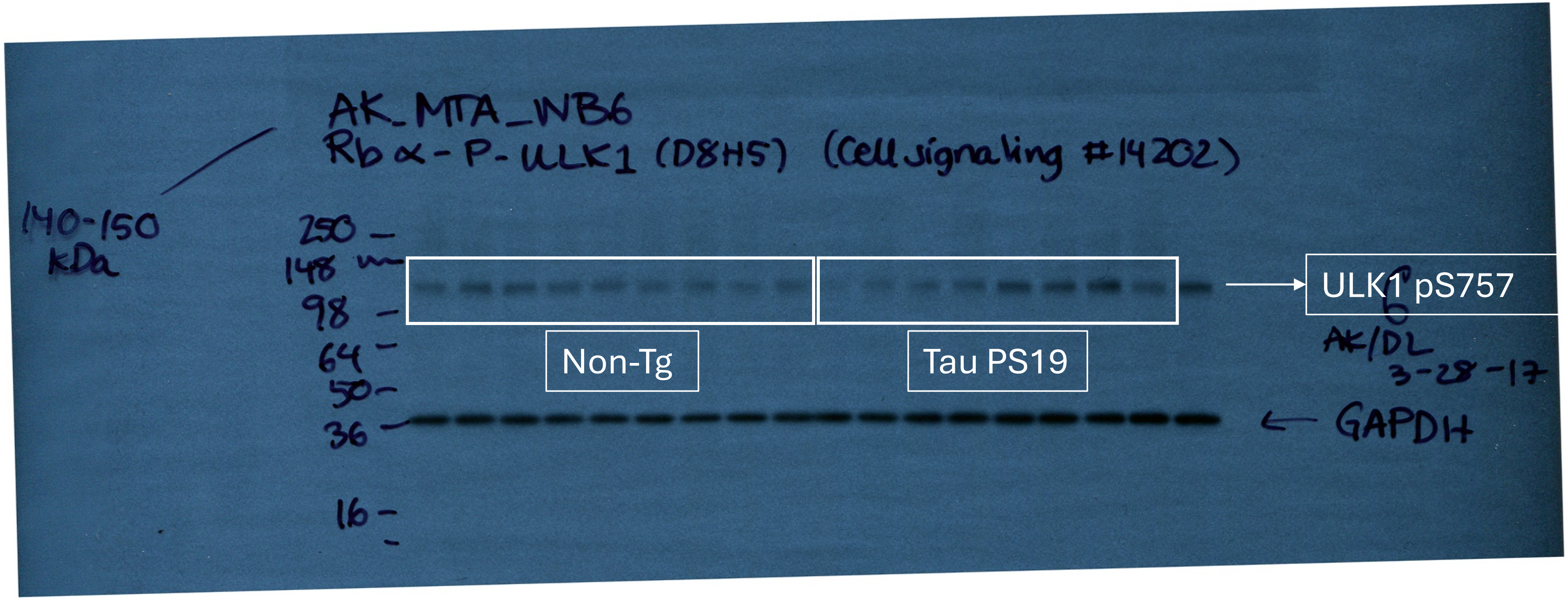

ULK1 pS757, Fig 4 F

AKMCMTA-WCL-WB3 07-14-19

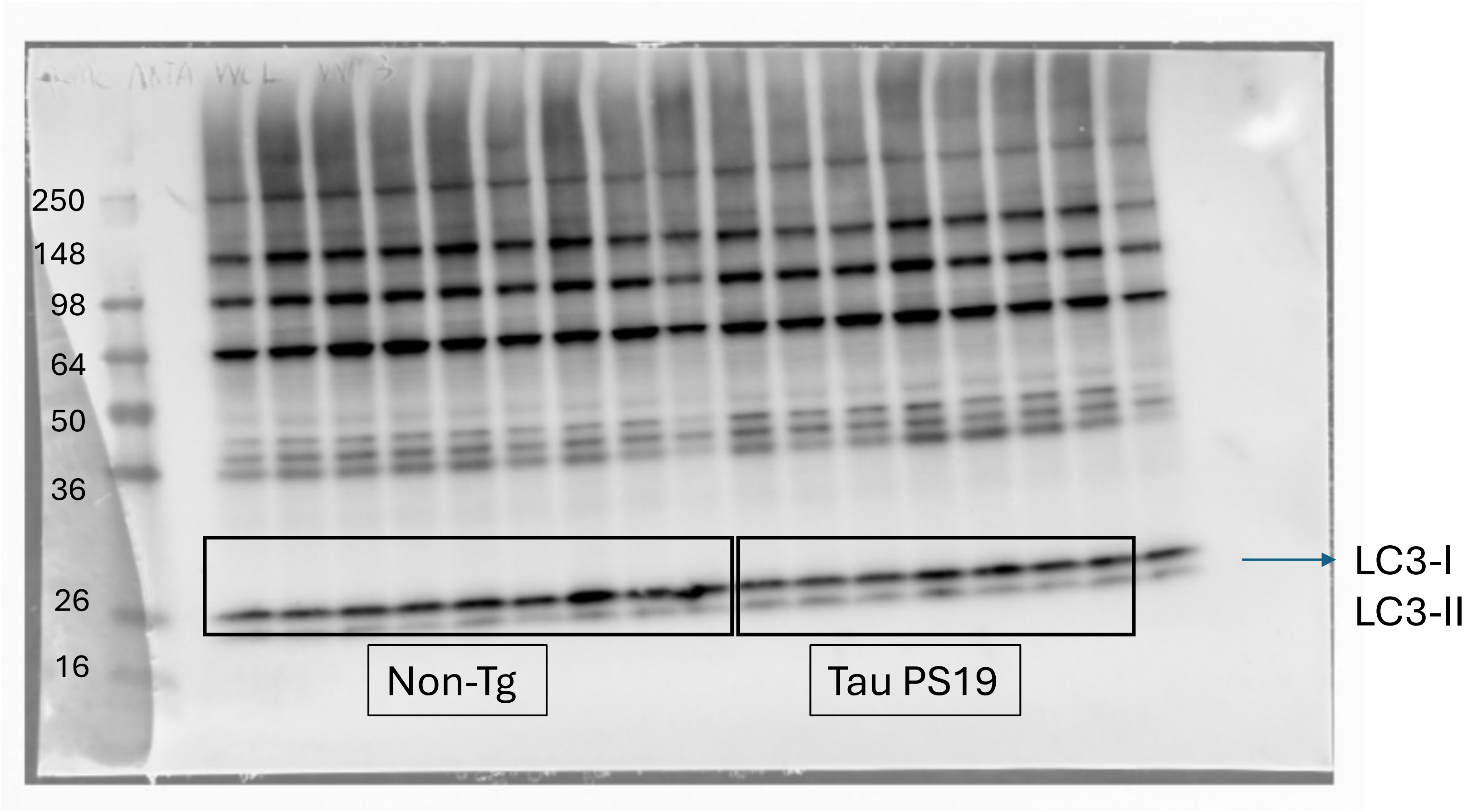

LC3-I, LC3-II, Fig 4 F

AK_MTA_WB14

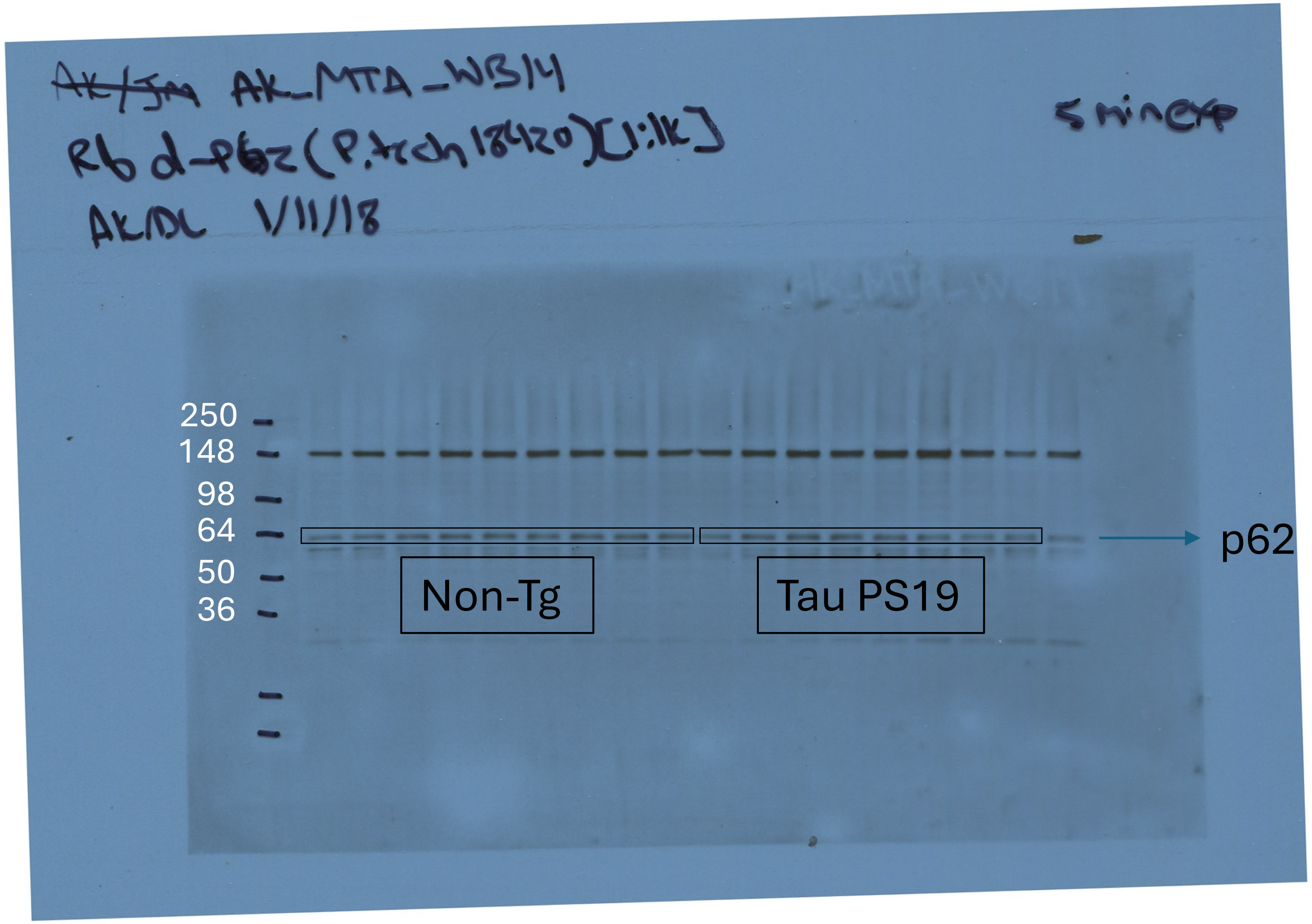

p62, Fig 4 F

S3F10-0713-135028_pub

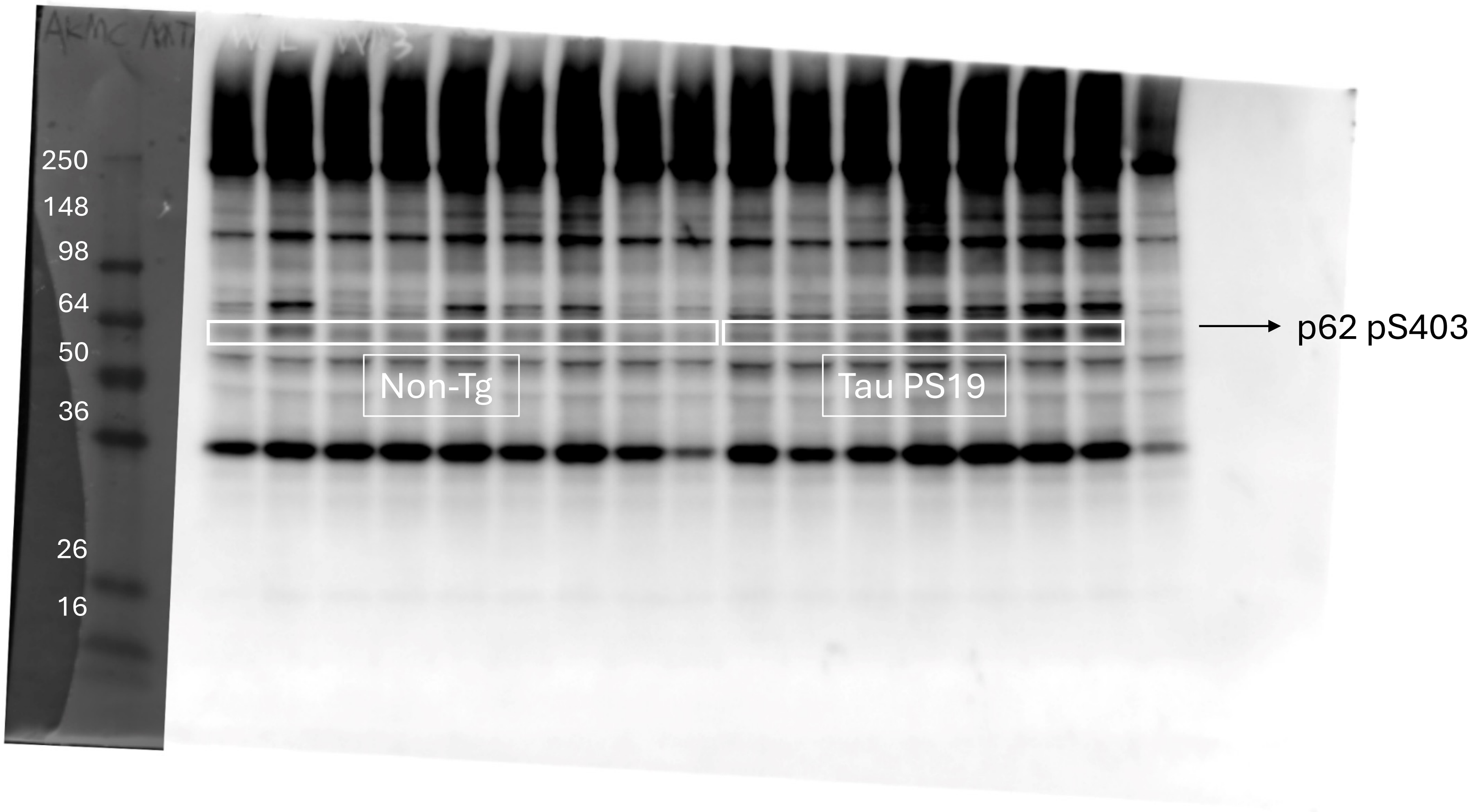

p62 pS403, Fig 4 F

Figure 5 Raw Blots

MCLB-01-WB1

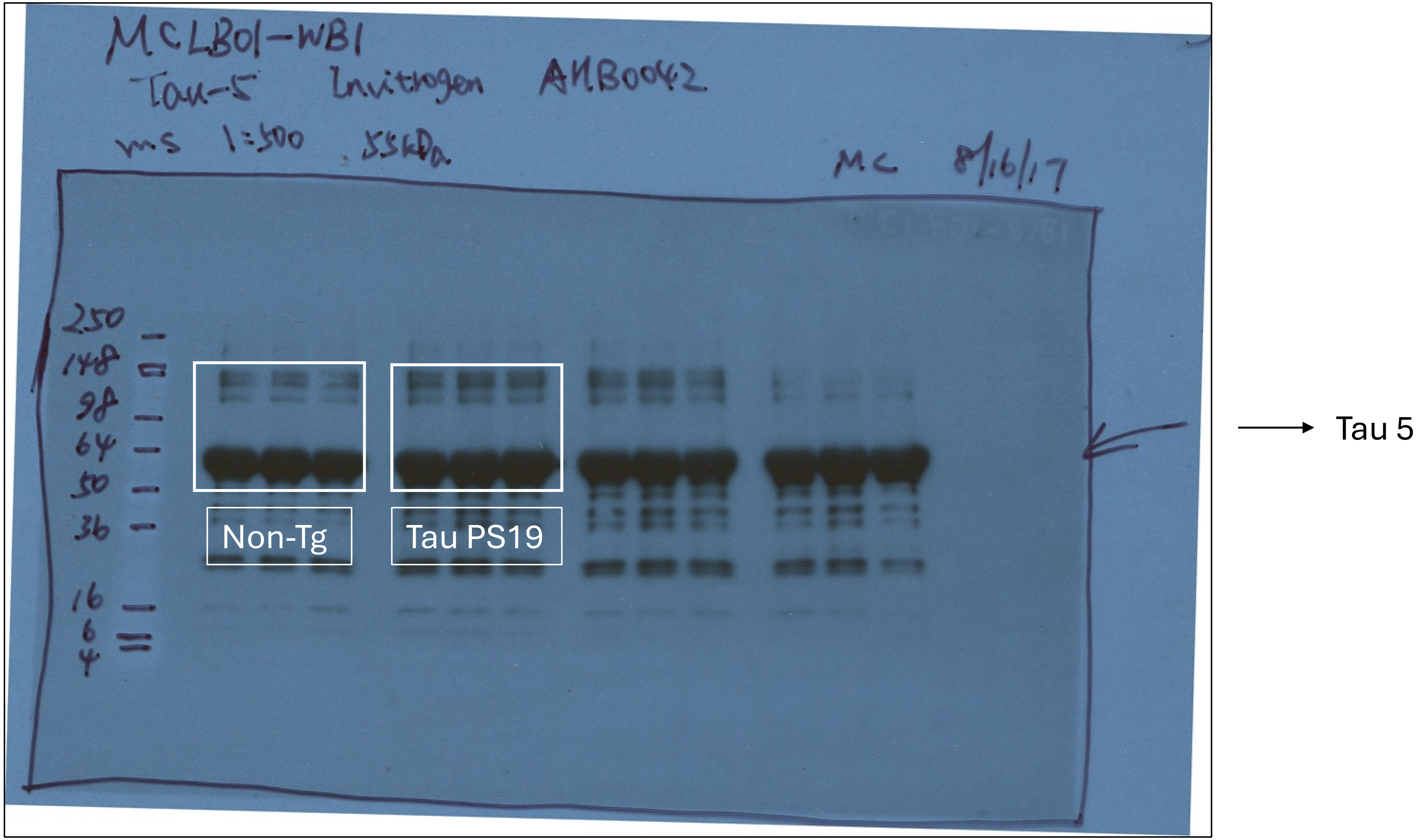

Tau-5, Fig 5 D

MCLB01-WB2

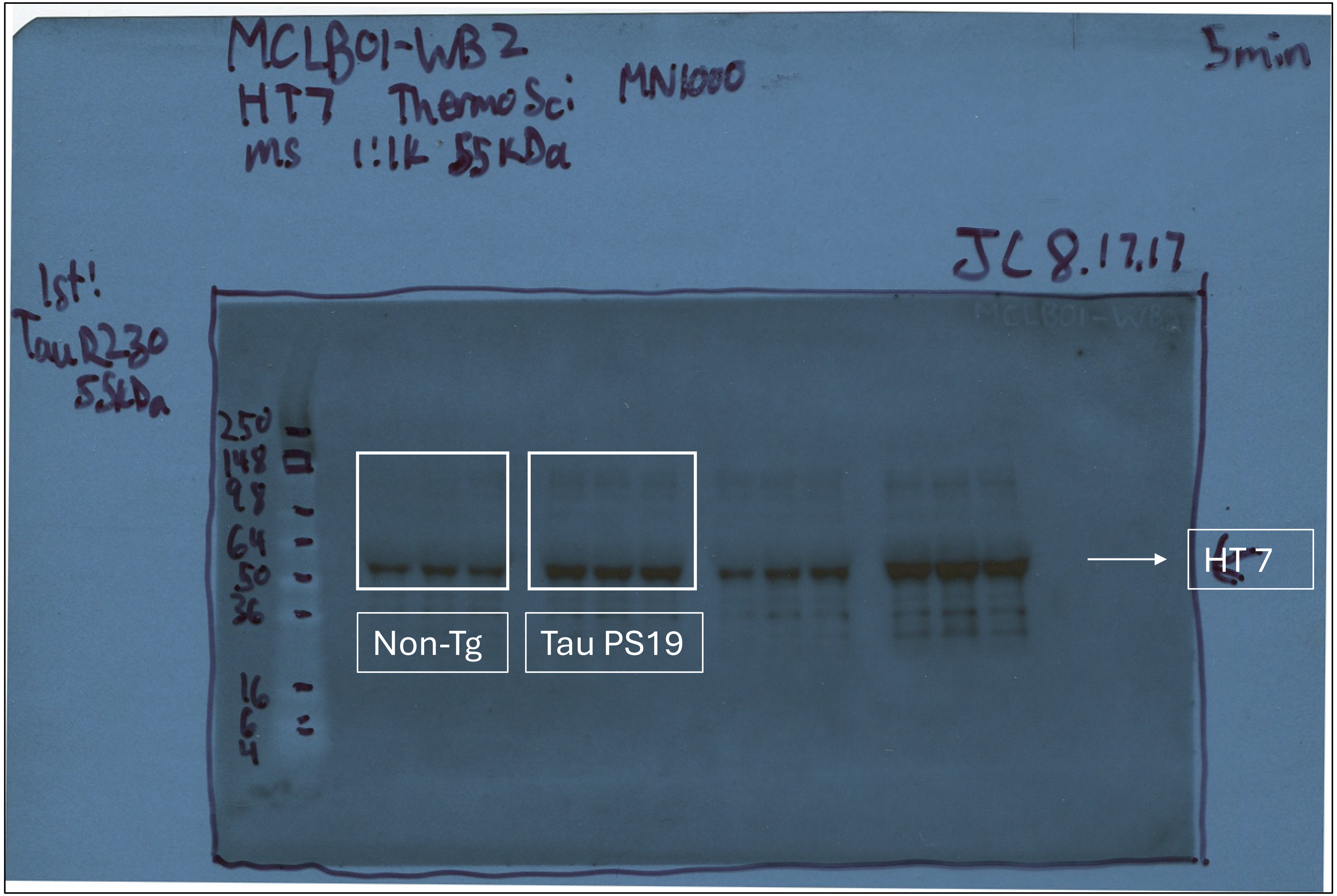

HT-7, Fig 5 D

Figure 6 Raw Blots

MC046-24h-WB4-mTOR-exp 1s-a RAW

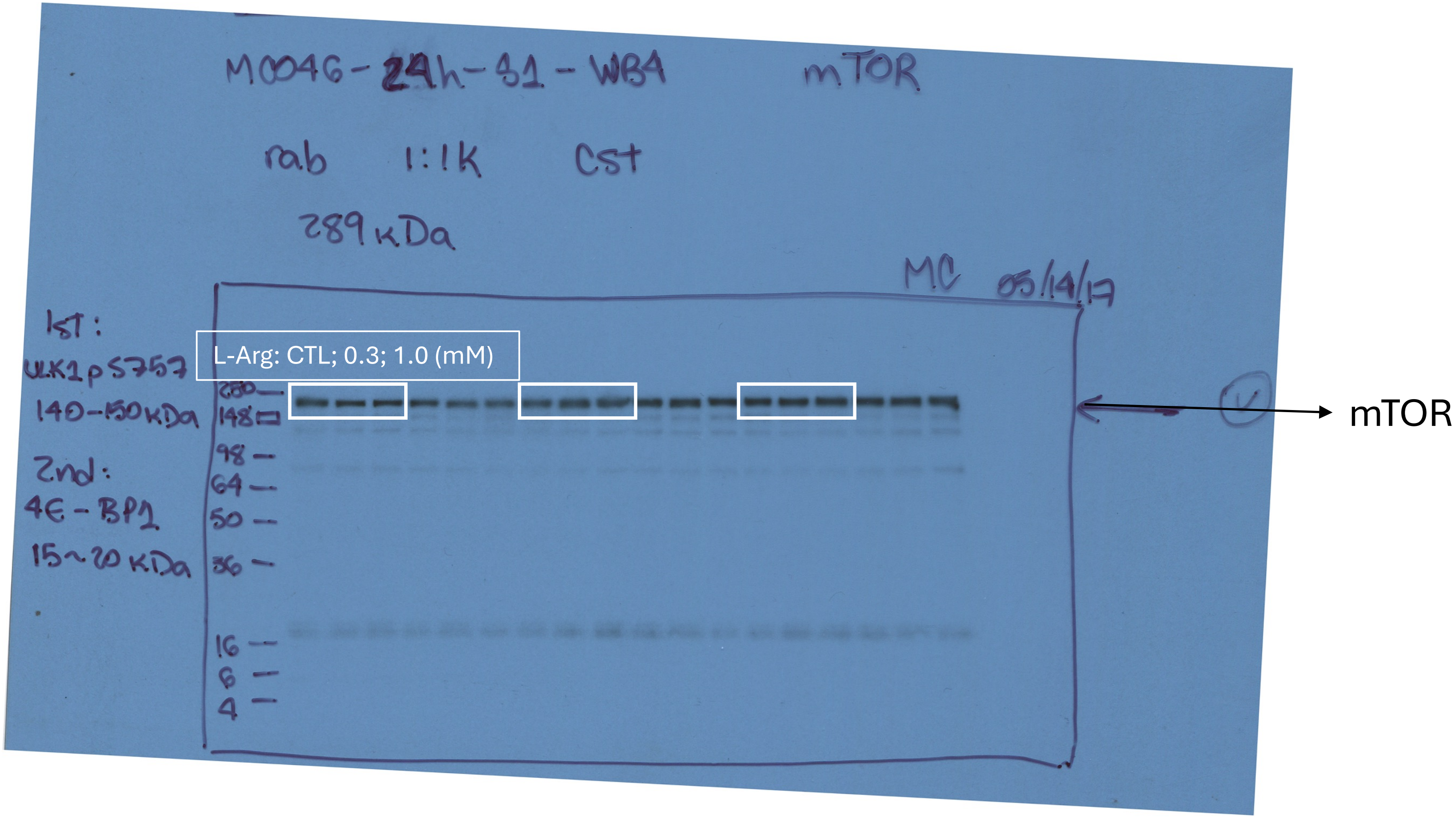

mTOR, Fig 6 A

MC046S1WB1- mTOR pS2448--exp 1s-b RAW

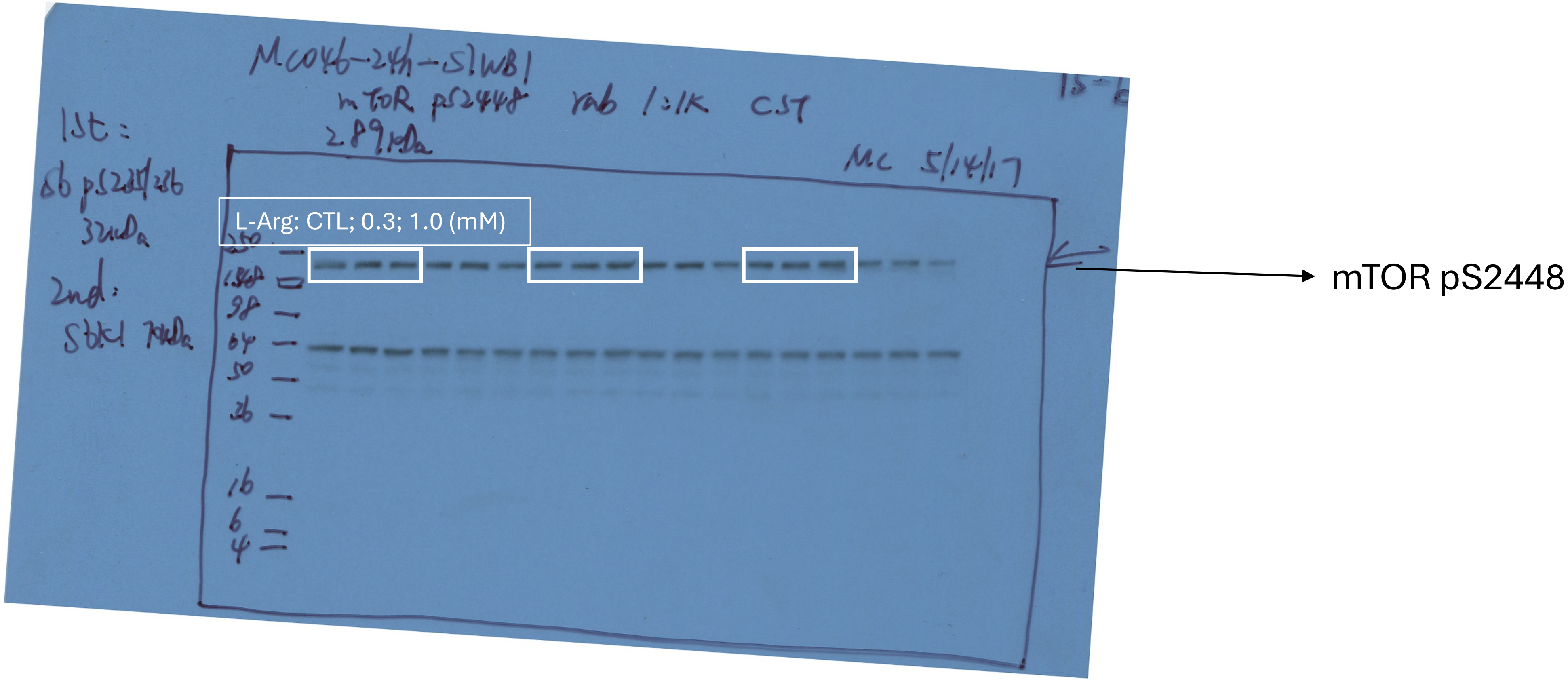

mTOR pS2448, Fig 6 A

MC046-24h-WB2-S6-exp 1s-a RAW

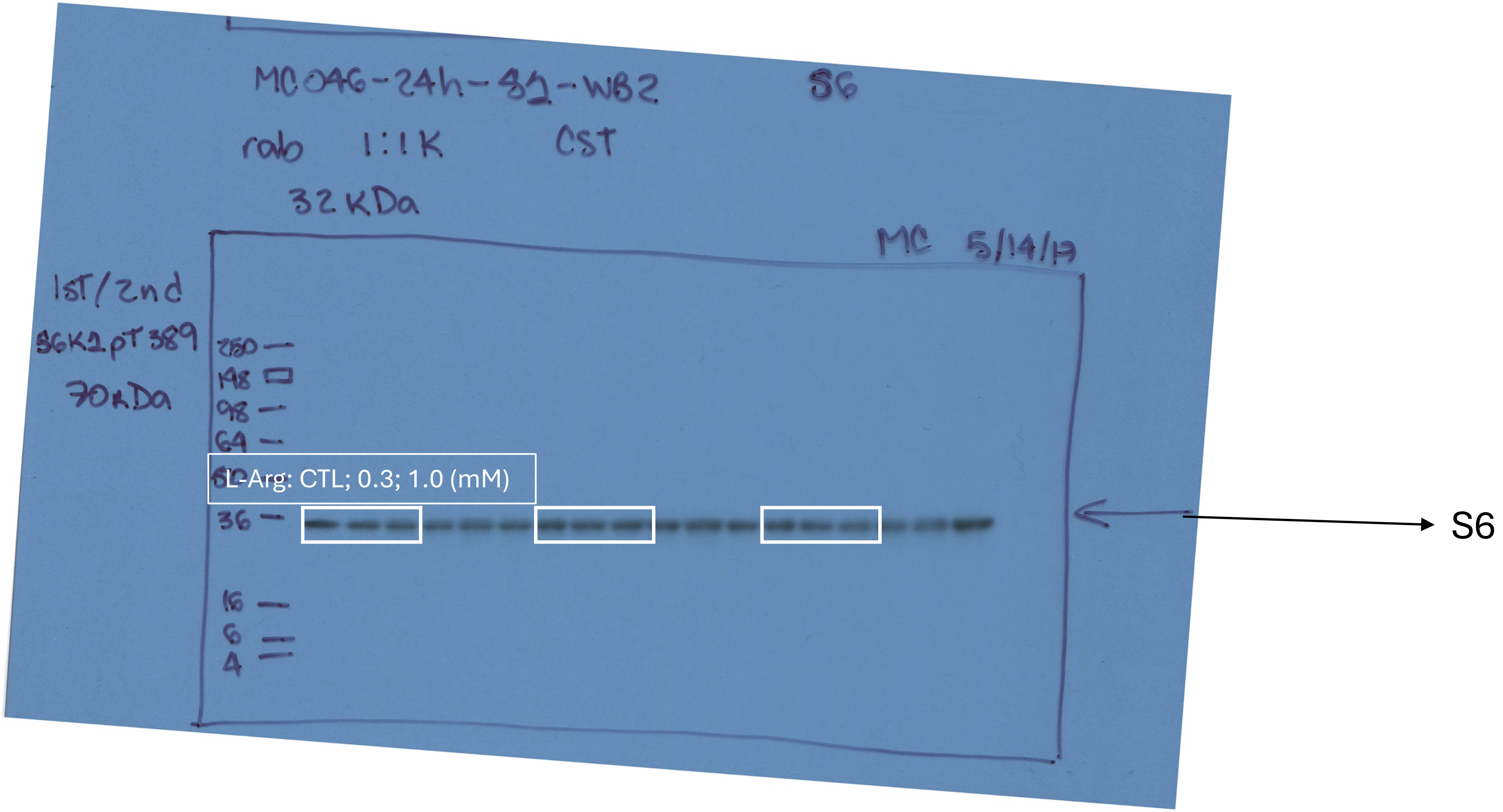

S6, Fig 6 A

MC046-24h-WB1-S6 pS235-236-exp 1min RAW

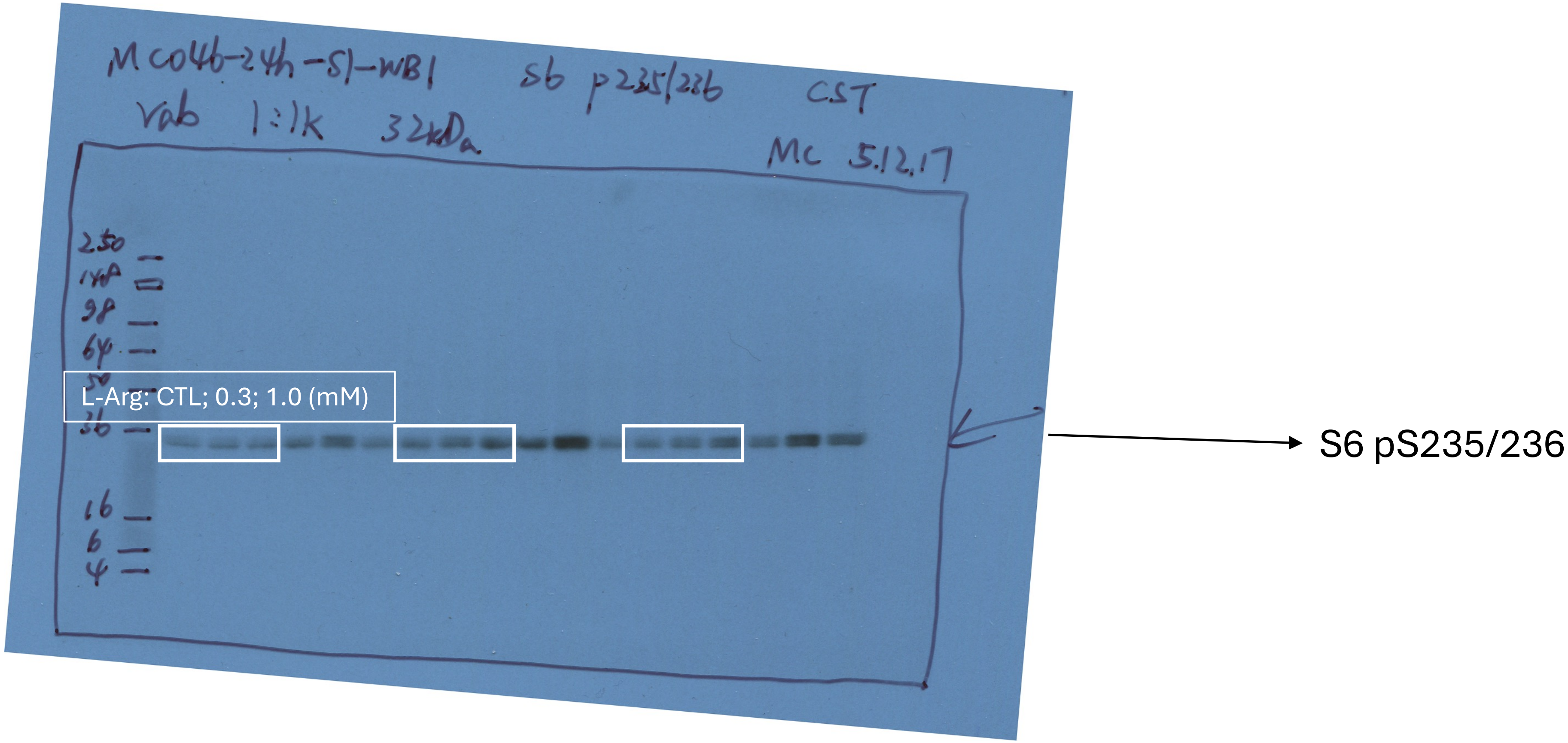

S6 pS235/236, Fig 6 A

MC046-24h-WB4- 4E-BP1-exp 1s-a RAW

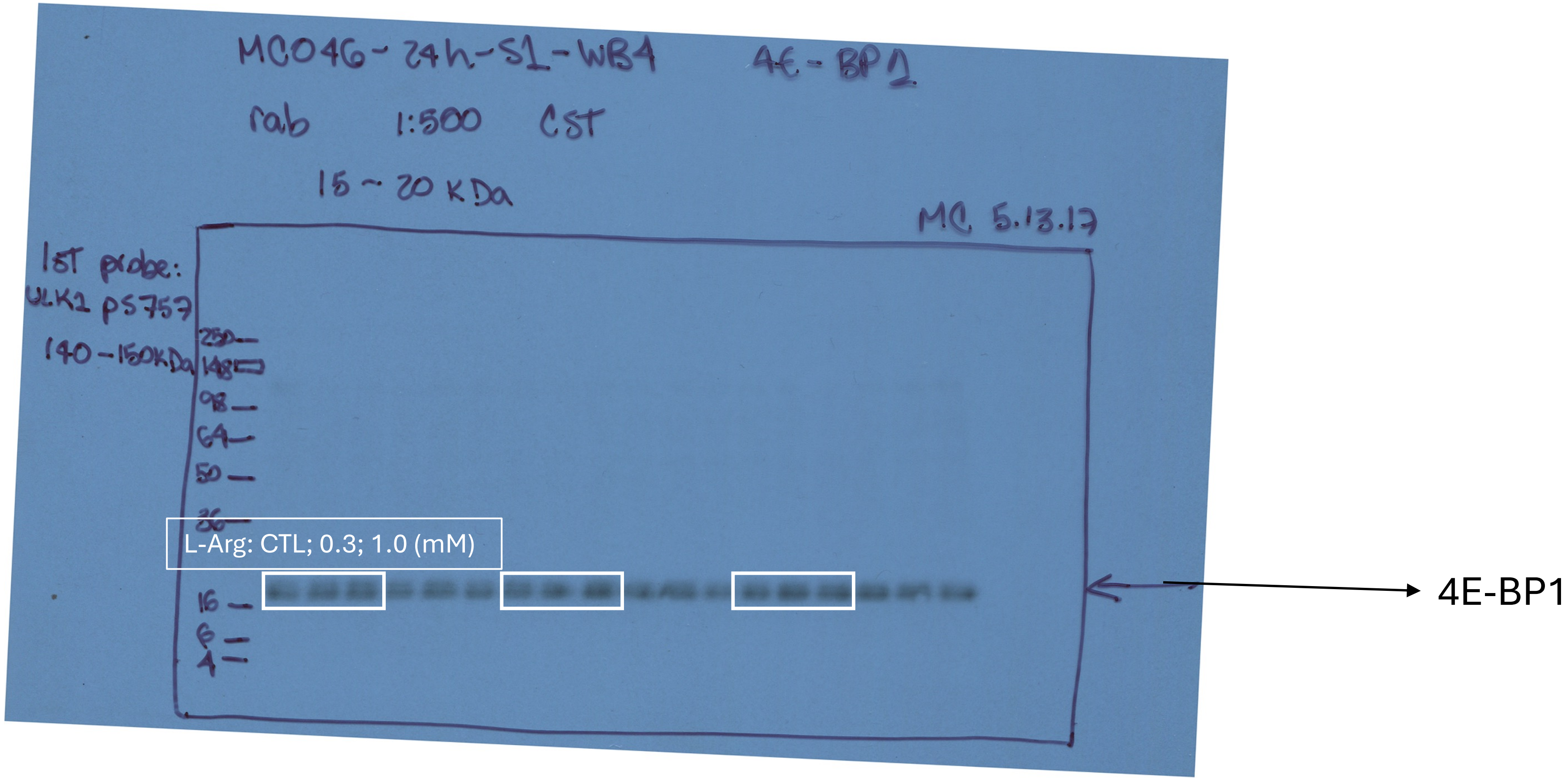

4E-BP1, Fig 6 A

MC046-24h- WB3- 4E-BP1 pT37-46-exp 5min RAW

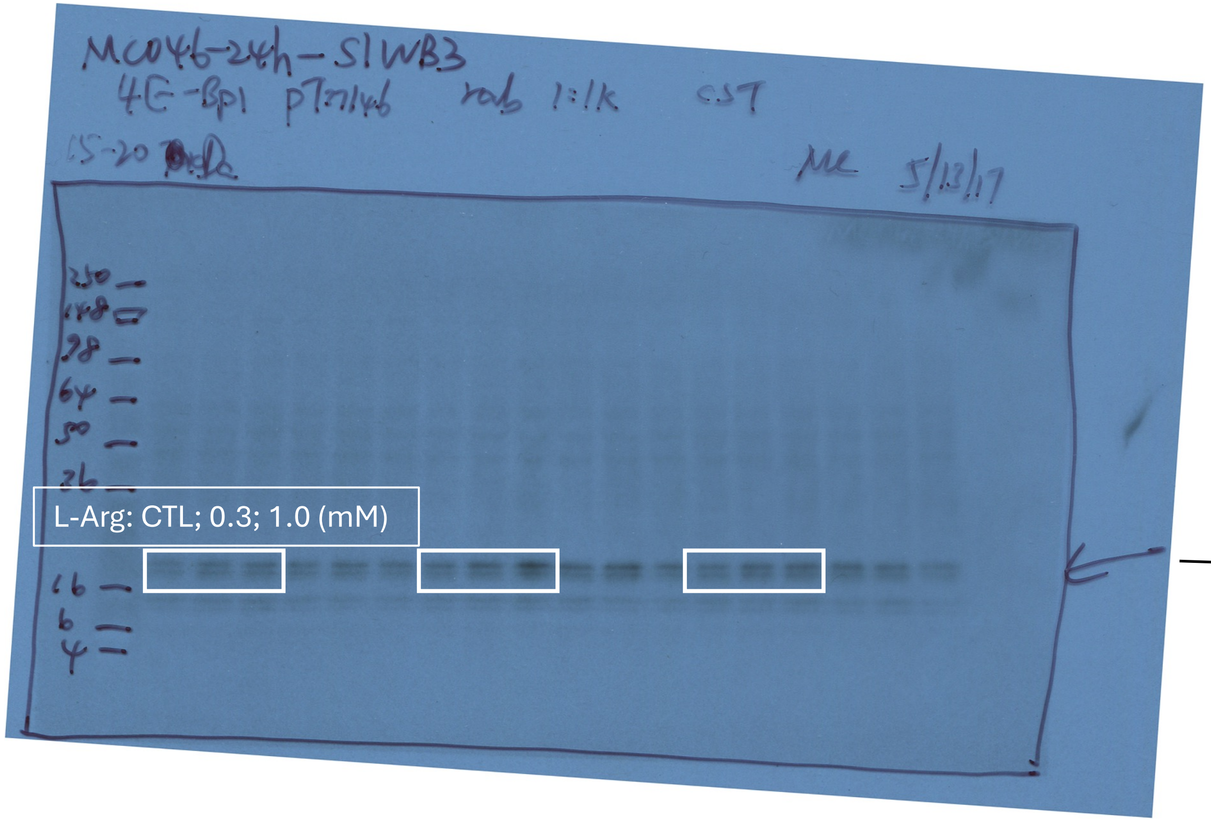

4E-BP1 pT37/46

4E-BP1 pT37/46 , Fig 6 A

MC046-WB3-5s RAW

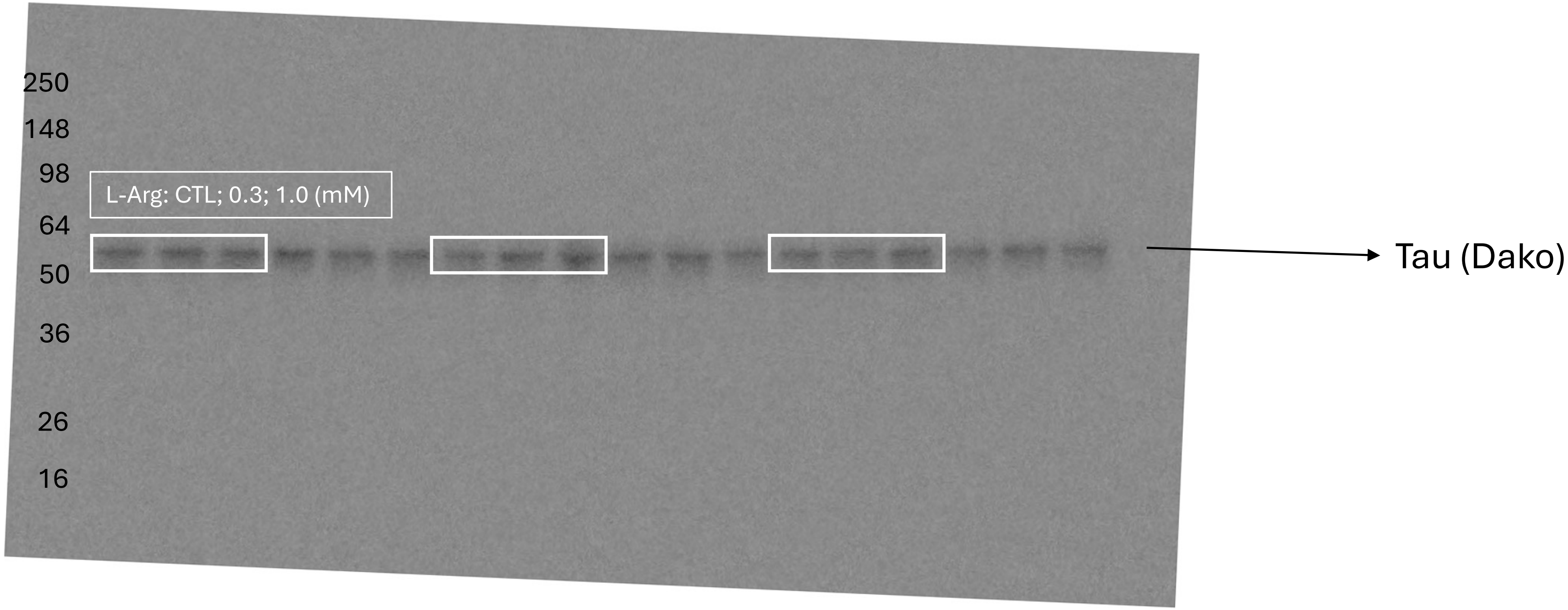

Tau (Dako) , Fig 6 A

Figure 7 Raw Blots

MK08 WB1 5 min RAW

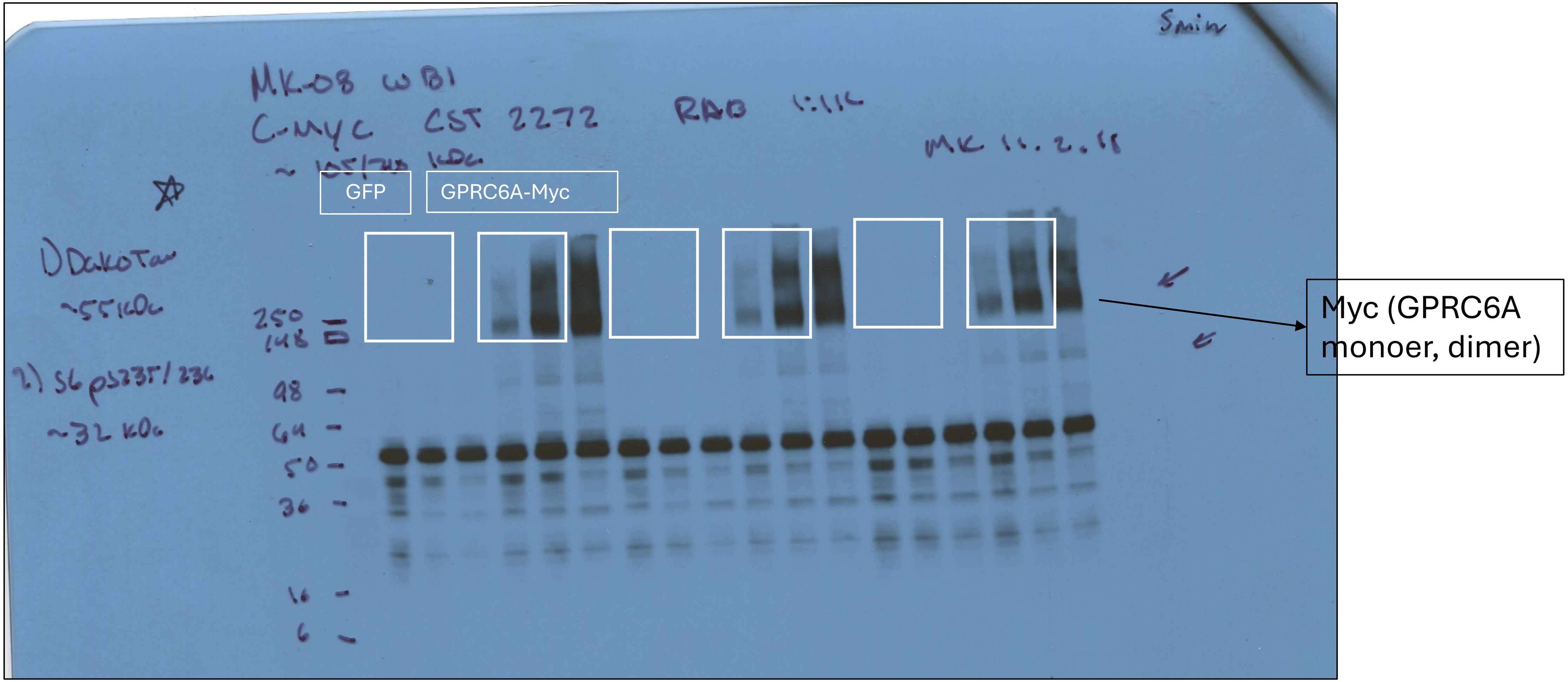

Myc (GPRC6A monoer, dimer), Fig 7 A

MK03 WB9 5 min RAW
(21.02.26_13.09.45_S3_F05_PUB_300_RAW)

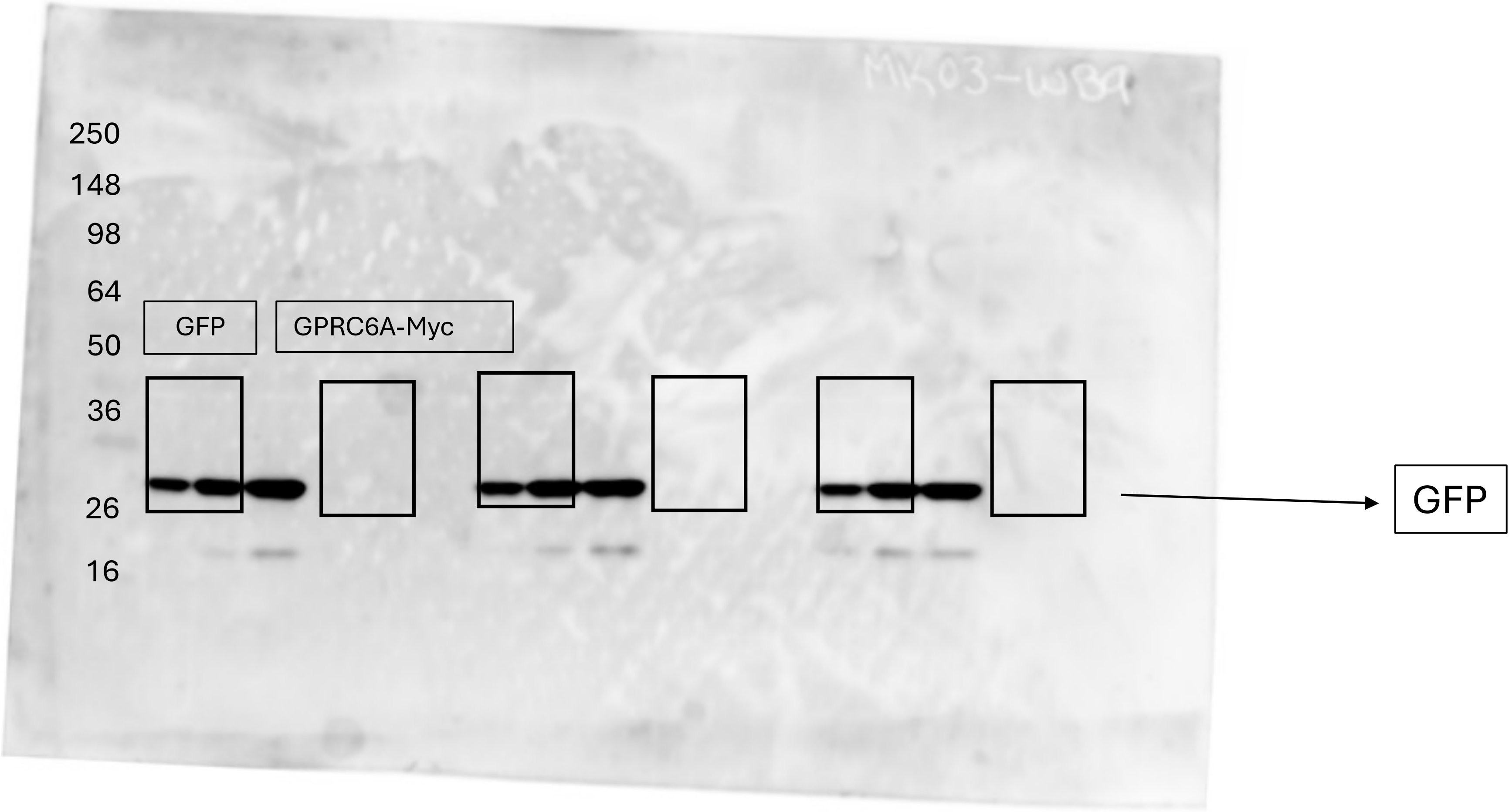

GFP, Fig 7 A

MK03 WB11 RAW
(21.03.03_13.59.47_S3_F01_PUB_300 RAW)

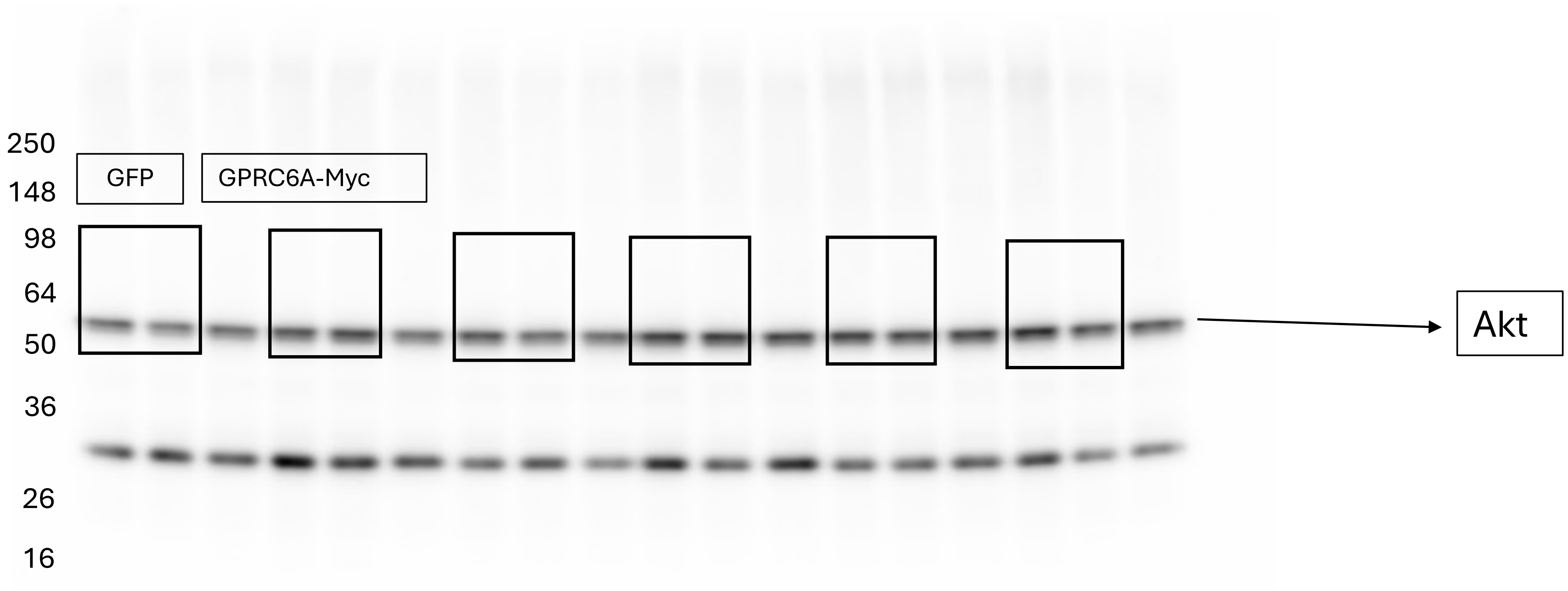

Akt, Fig 7 A

MK03WCL-WB6 1.5 min RAW

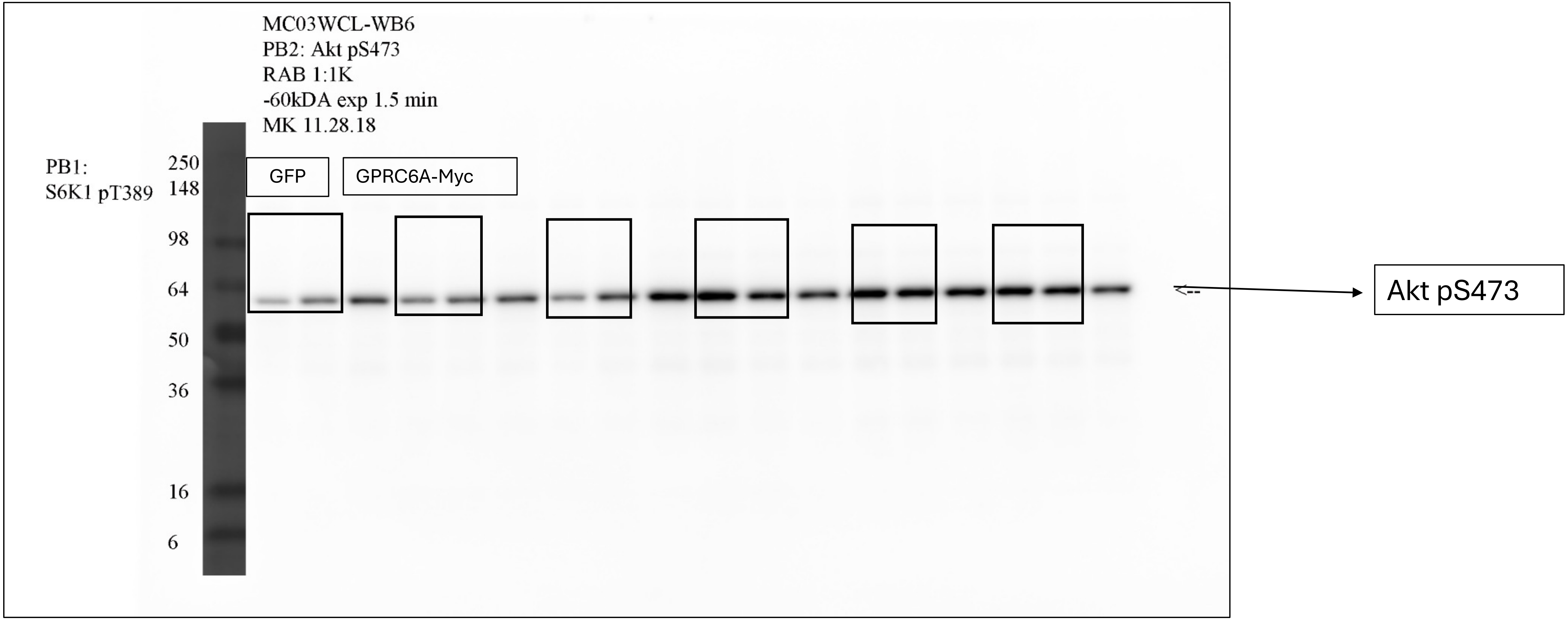

AKT pS473, Fig 7 A

MK03-WB7 1.5 min RAW

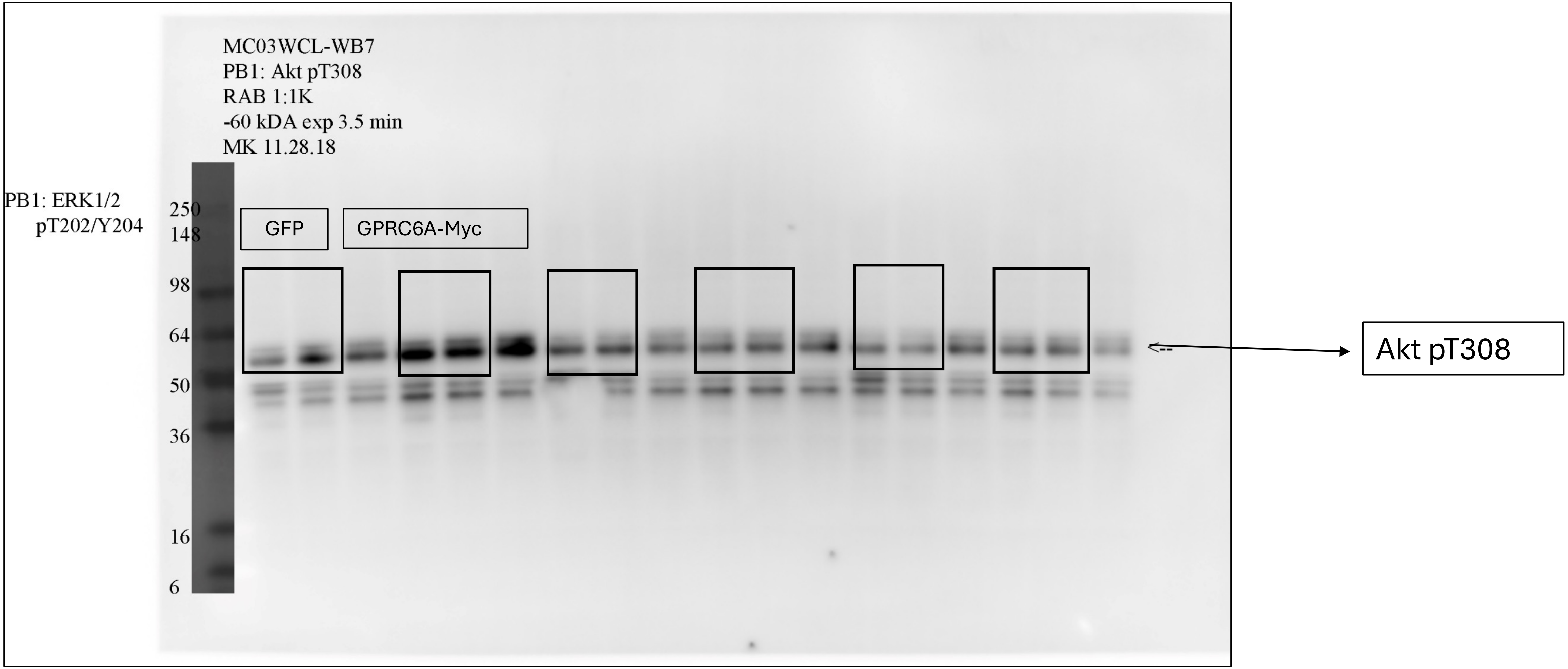

AKT pT308, Fig 7 A

MK03-WB12 RAW
(21.03.03_13.59.47_S3_F05_PUB_300 RAW)

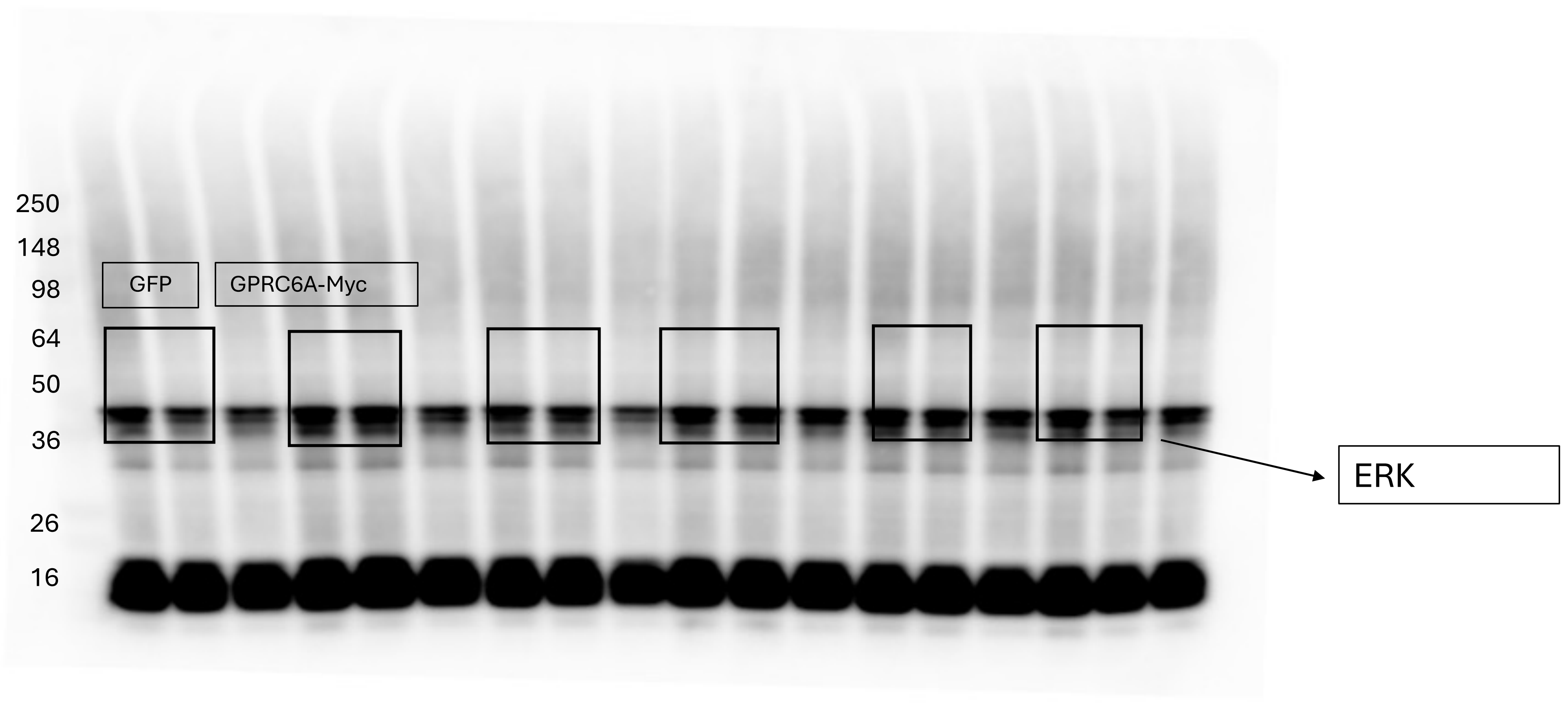

ERK, Fig 7 A

MK03-WB7 RAW

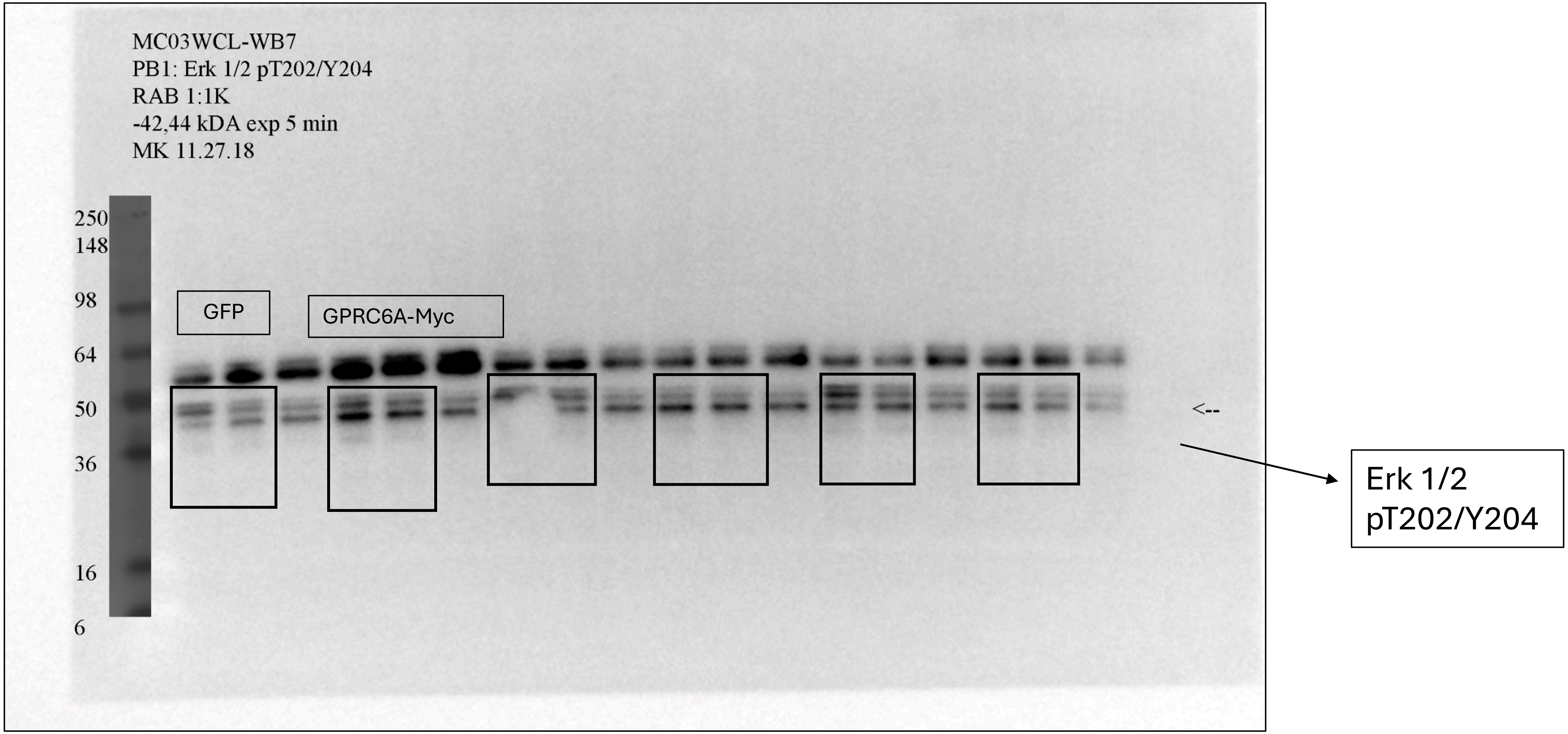

Erk 1/2 pT202/Y204, Fig 7 A

MK03-WB12 RAW
(21.03.04_13.39.46_S2_F06_PUB_300 RAW)

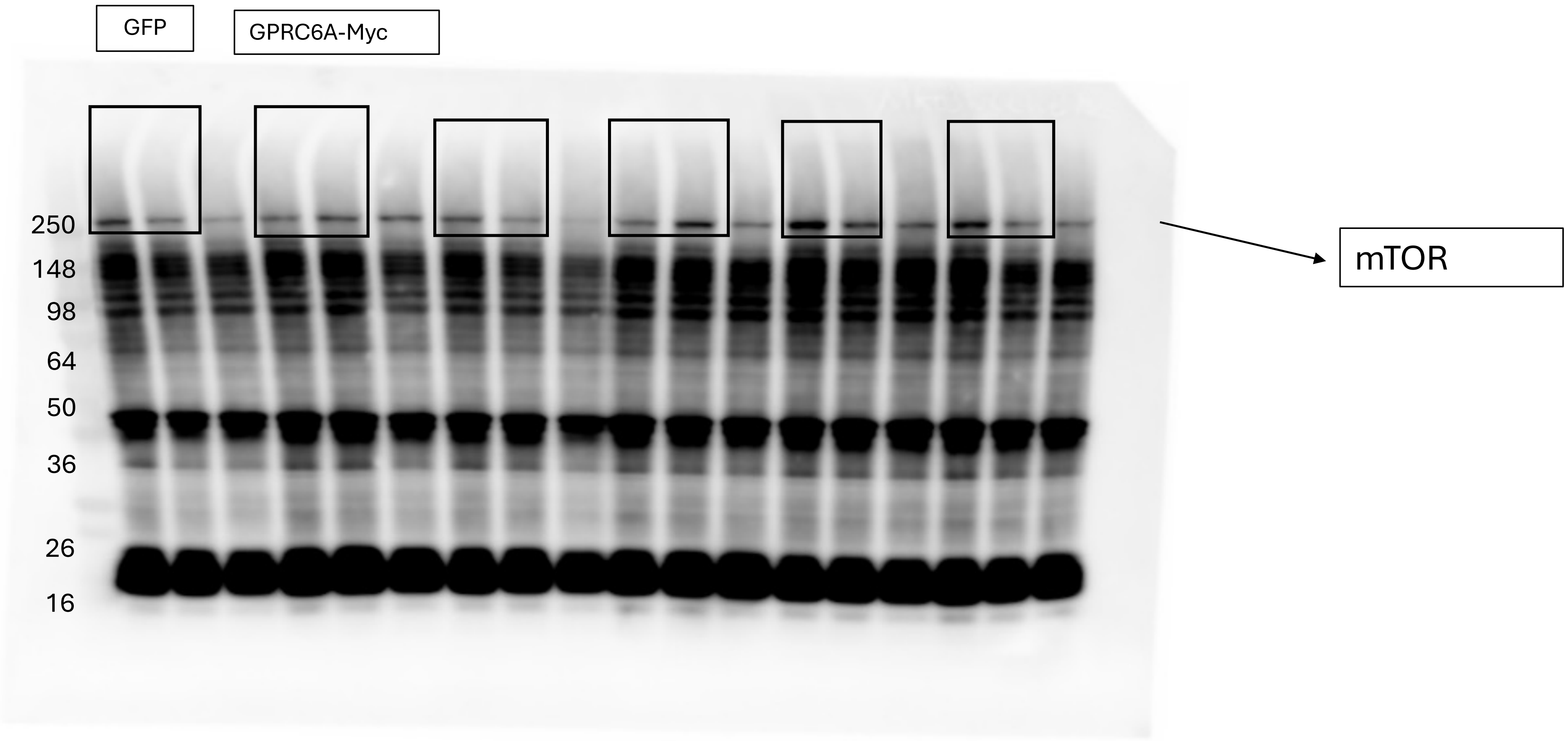

mTOR, Fig 7 E

MK03-WB9 RAW

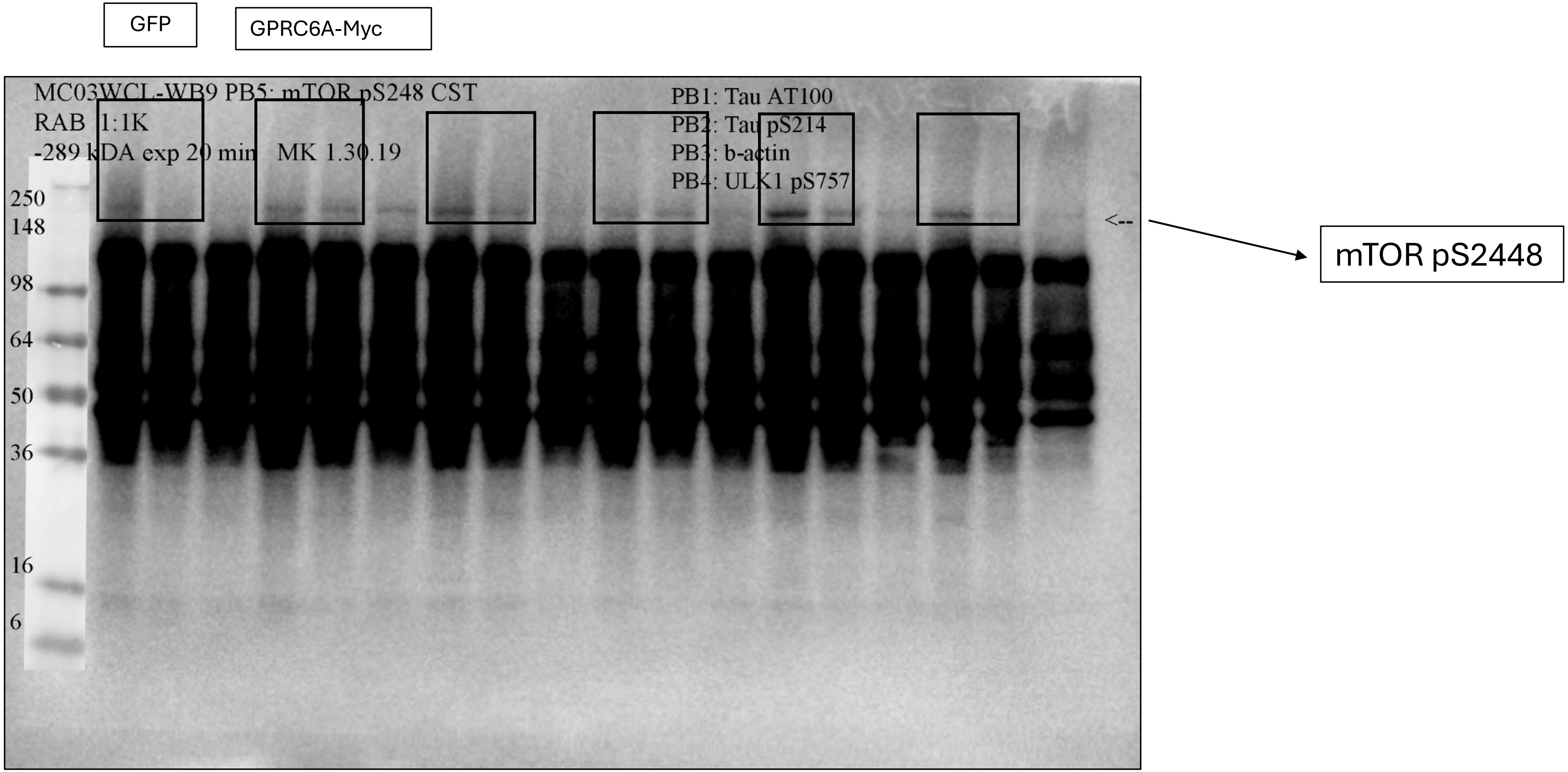

mTOR pS2448, Fig 7 E

MK03-WB11 RAW
(21.03.02_16.04.33_S3_F02_PUB_300_RAW_S6)

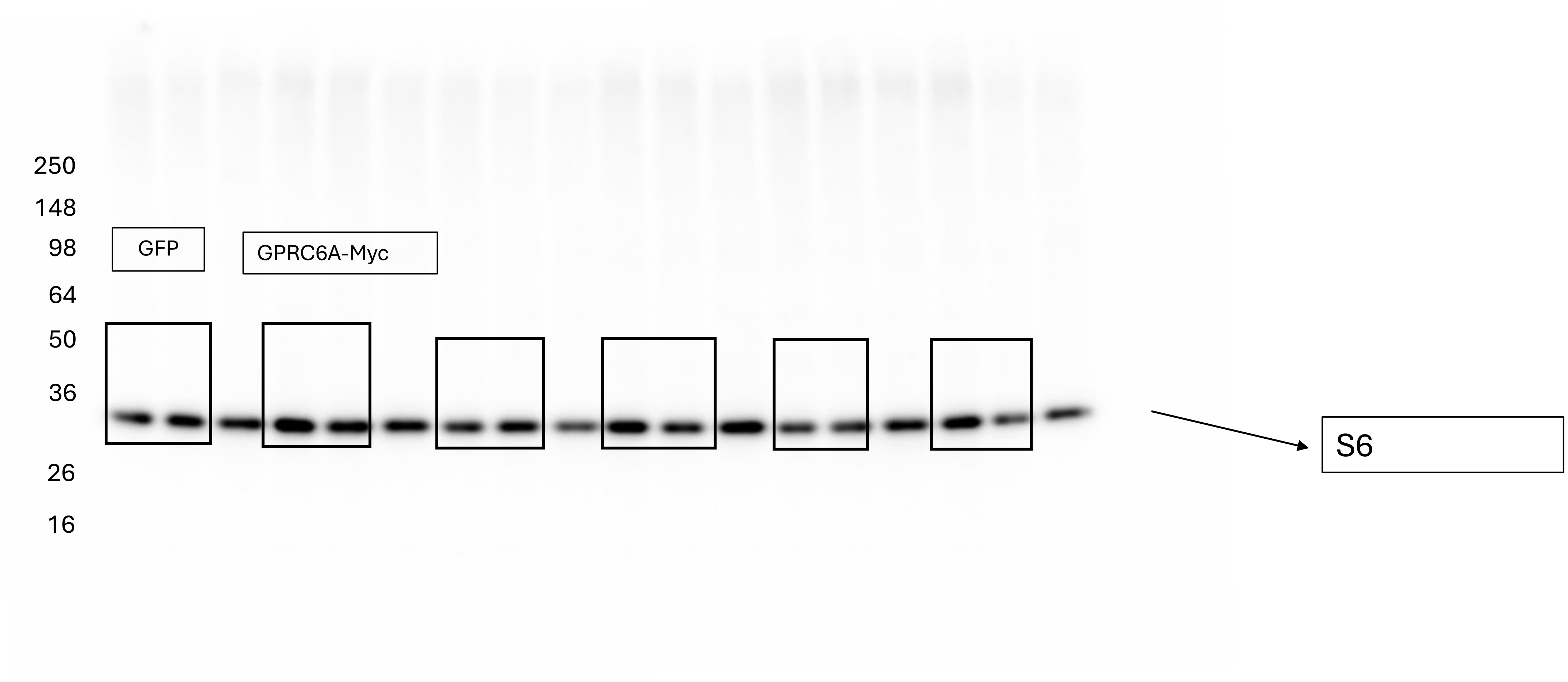

S6, Fig 7 E

MK03-WB1 RAW

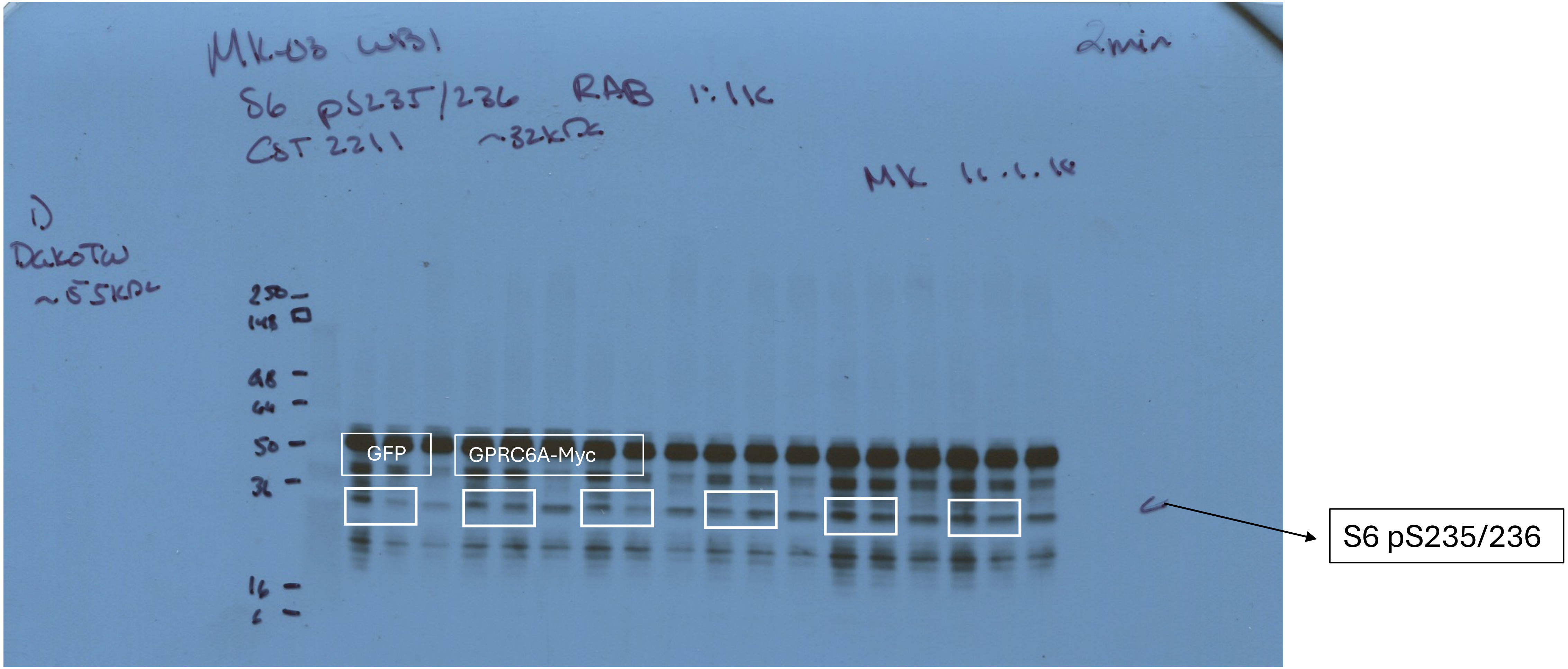

S6 pS235/236, Fig 7 E

MK03-WB2 RAW

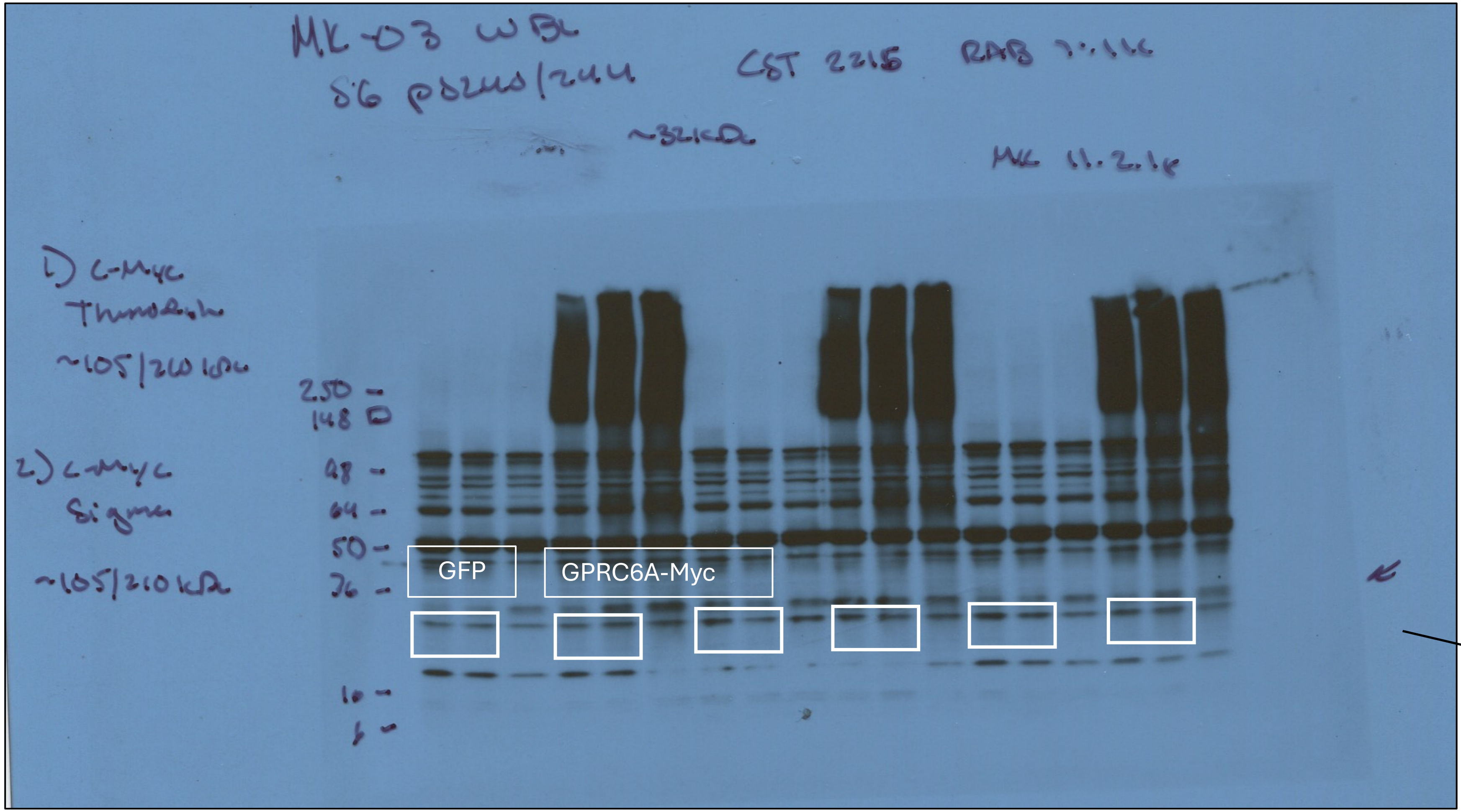

S6 pS240/244

S6 pS240/244, Fig 7 E

MK03-WB12 RAW
(21.03.02_16.04.33_S3_F02_PUB_300_RAW_4E-BP1)

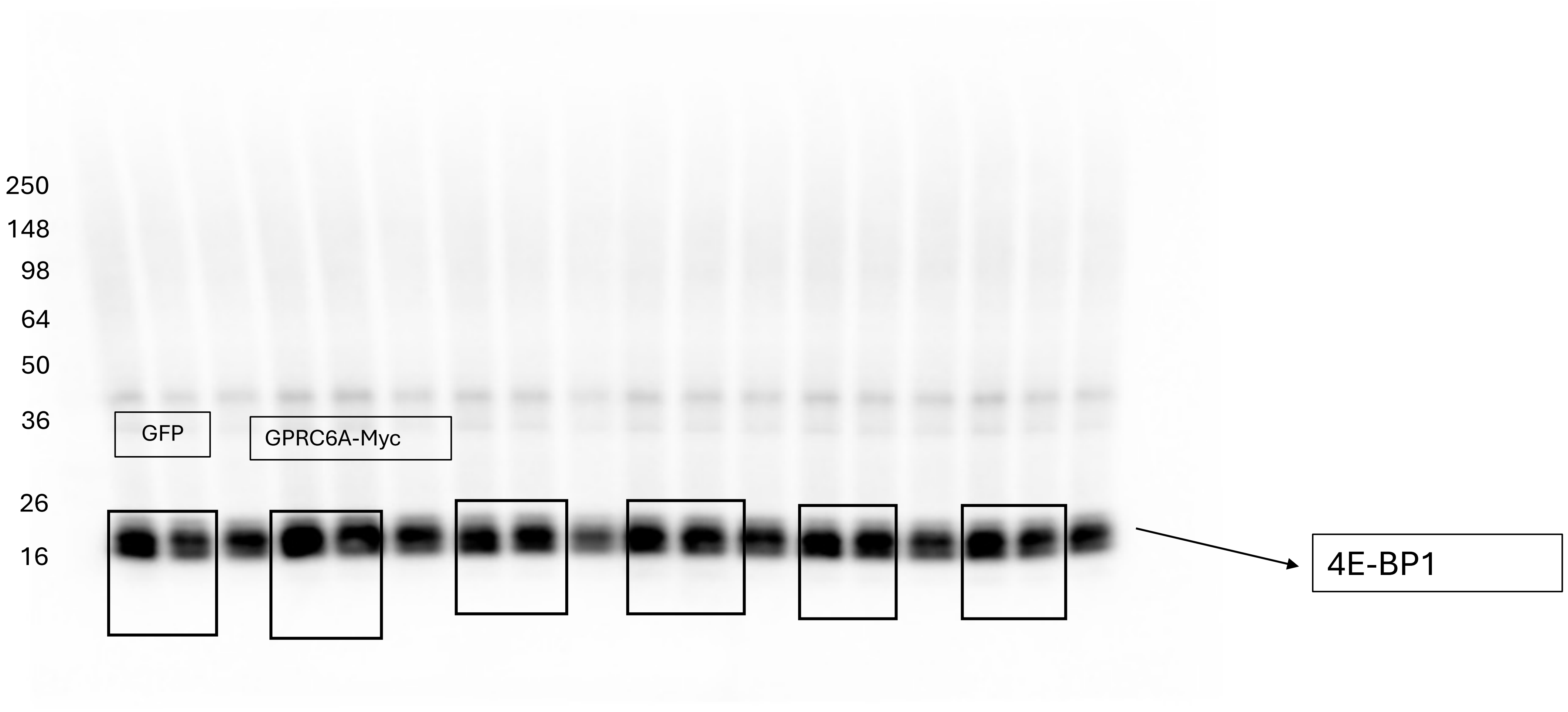

4E-BP1, Fig 7 E

MK03-WB7 RAW

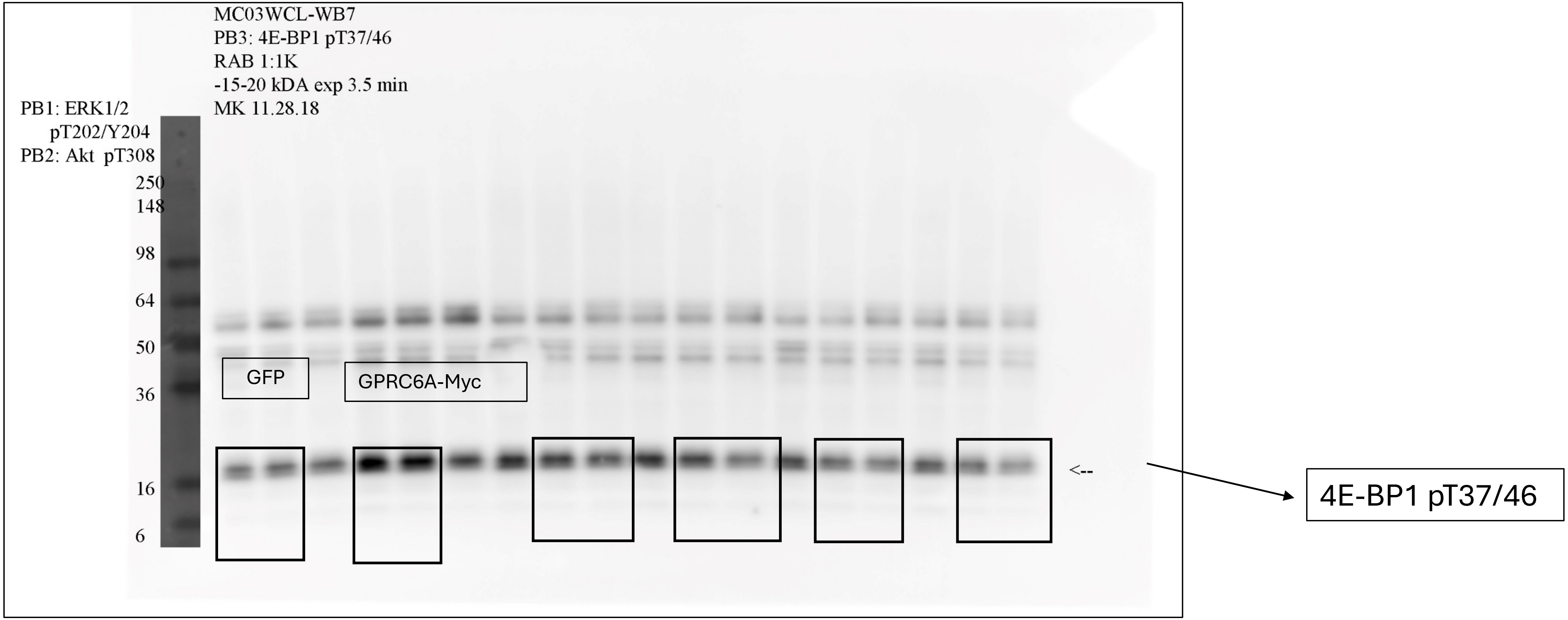

4E-BP1 pT37/46, Fig 7 E

MK03-WB6 RAW

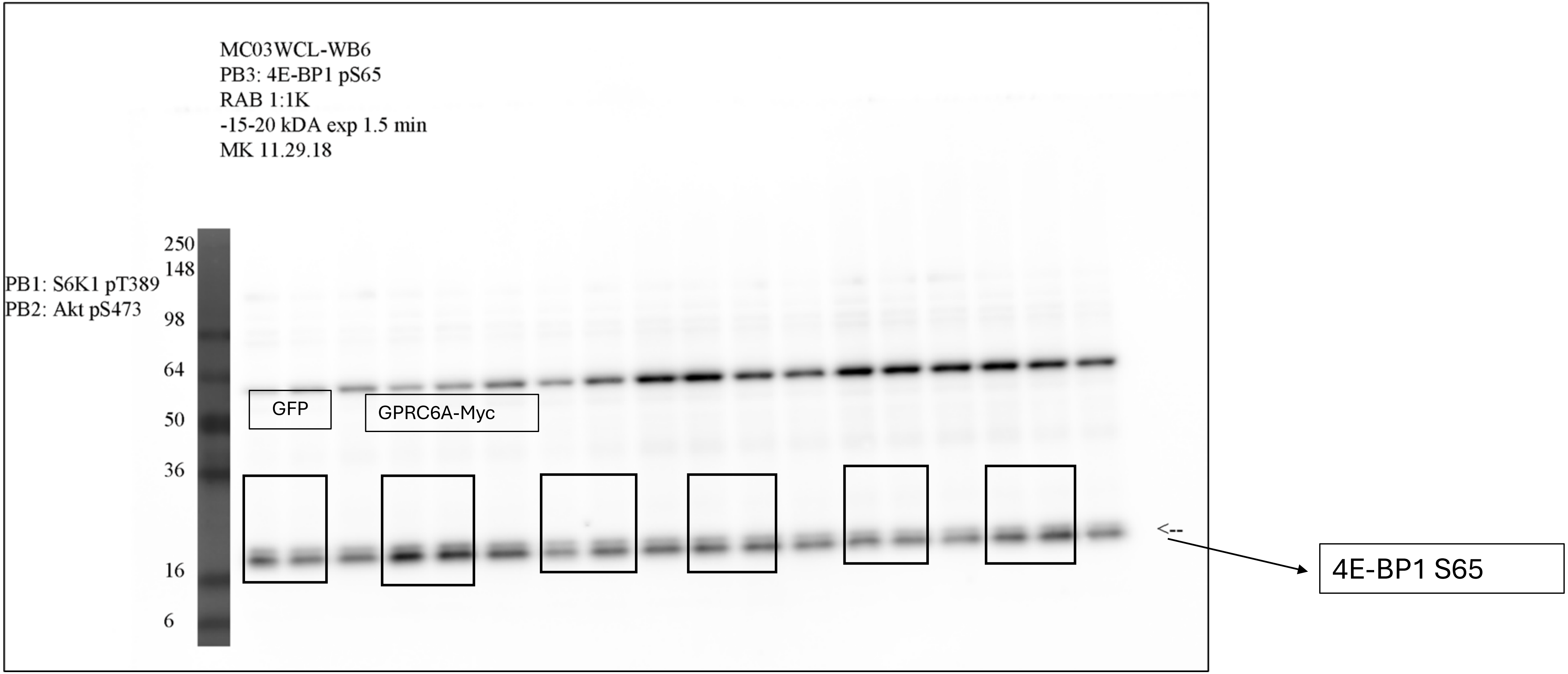

4E-BP1 S65, Fig 7 E

MK03-WB11 RAW
(21.03.04_13.42.06_S3_F02_PUB_300_RAW_ULK1)

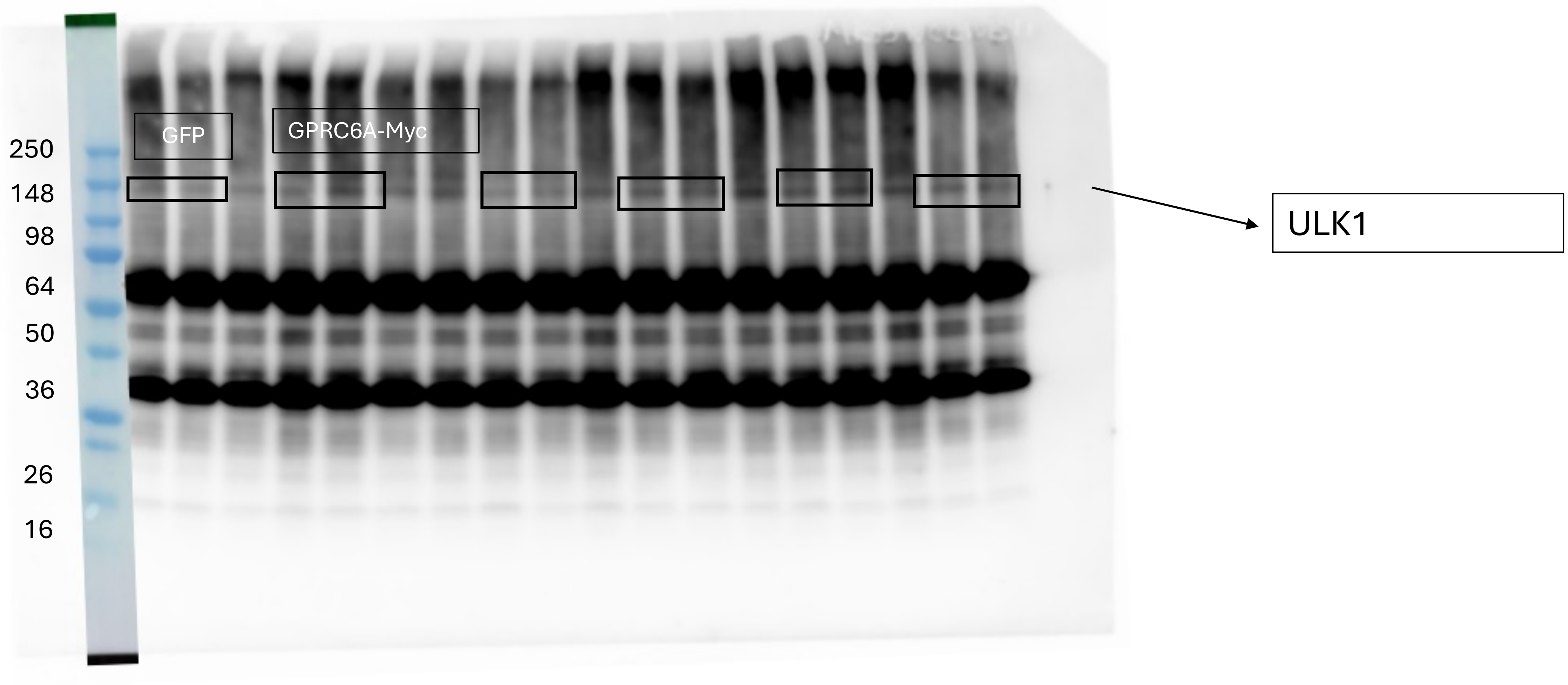

ULK1, Fig 7 I

MK03-WB9 RAW

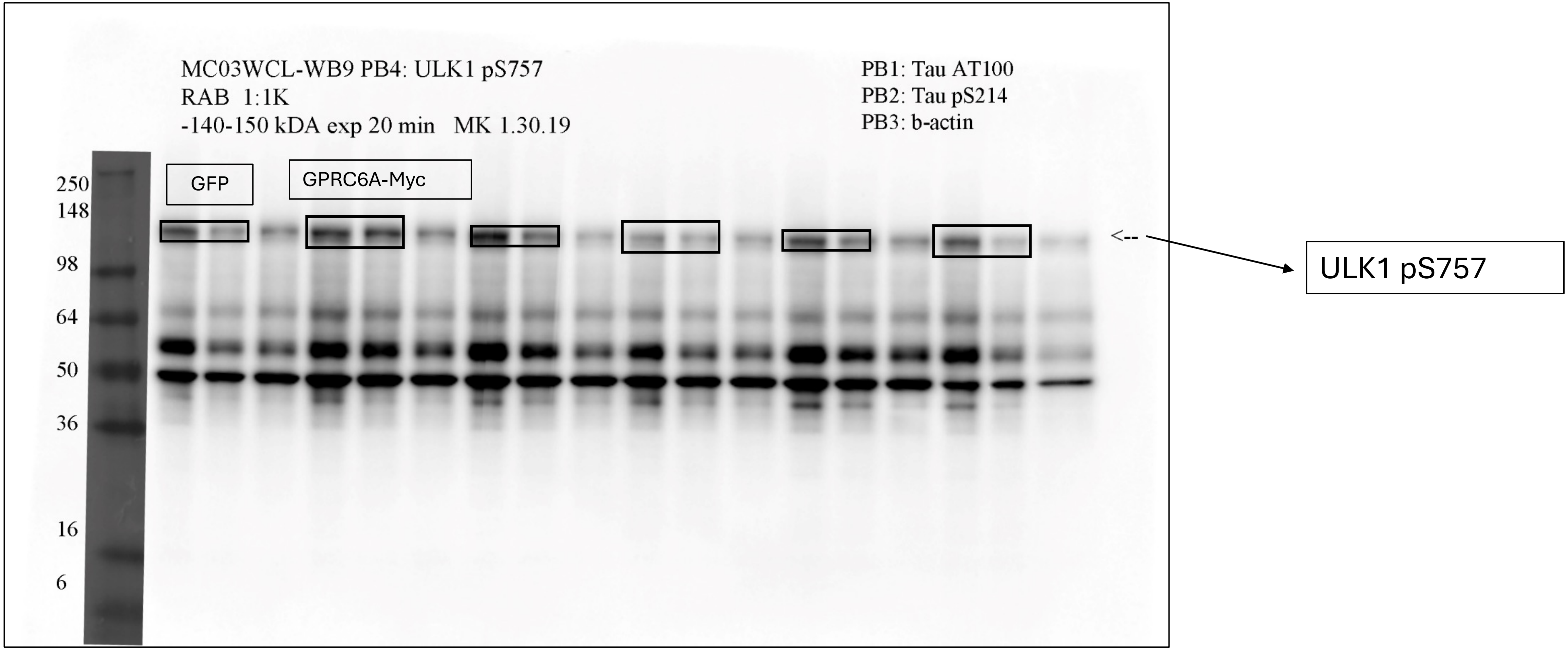

ULK1 pS757, Fig 7 I

MK03-WB6 RAW

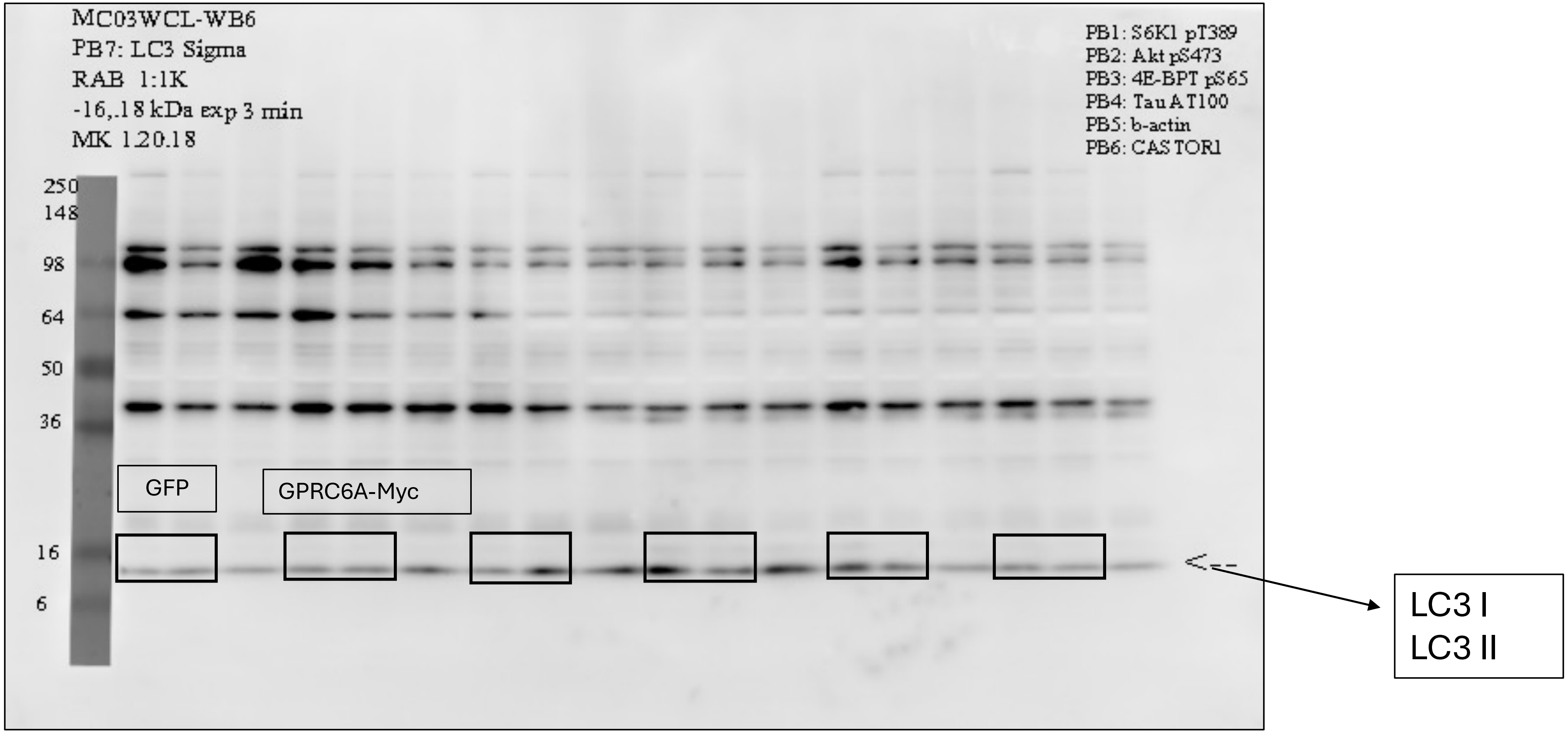

LC3 I, LC3 II, Fig 7 I

MK03-WB1 RAW

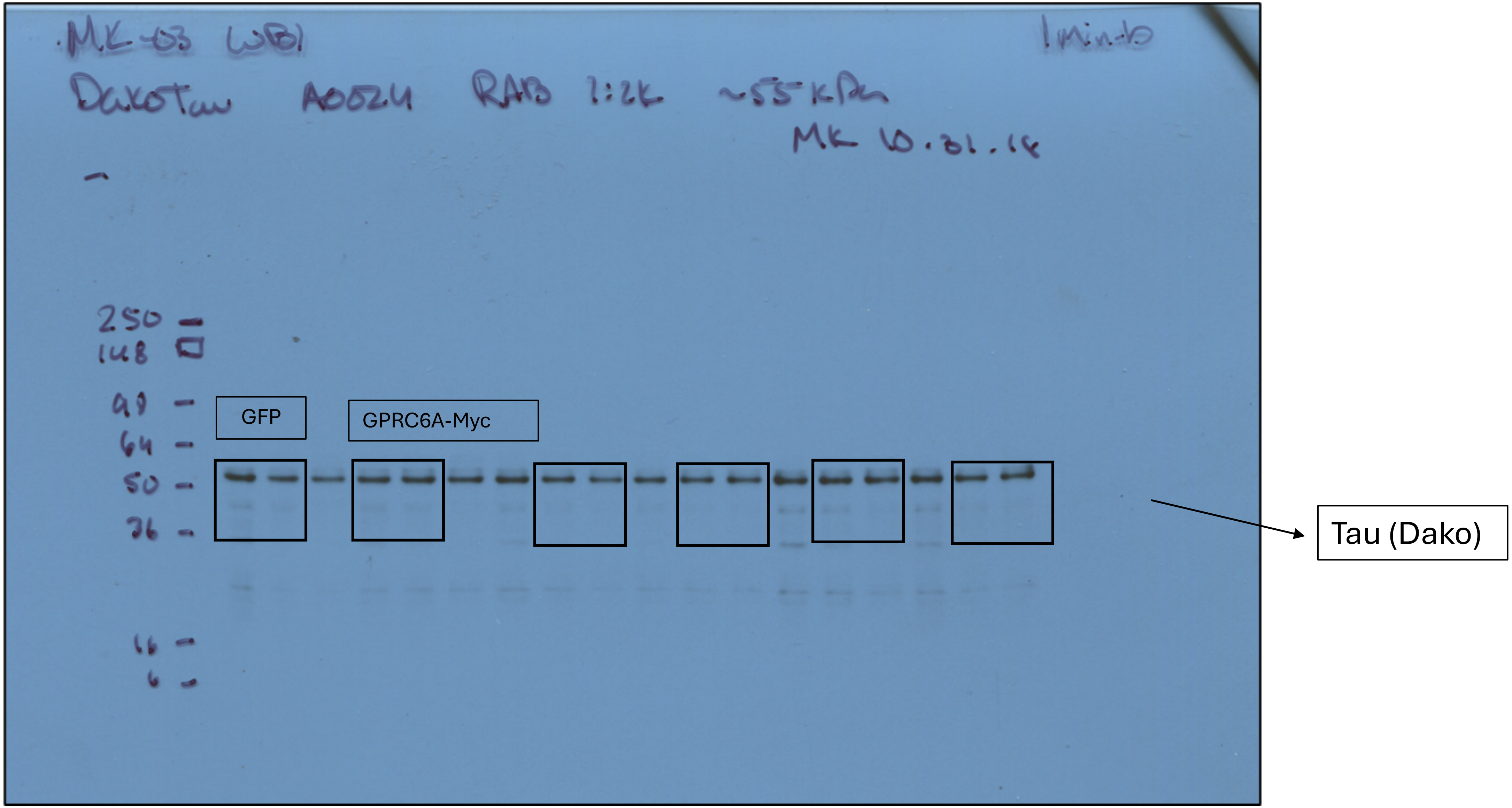

Tau (Dako), Fig 7 I

MK03-WB9 RAW

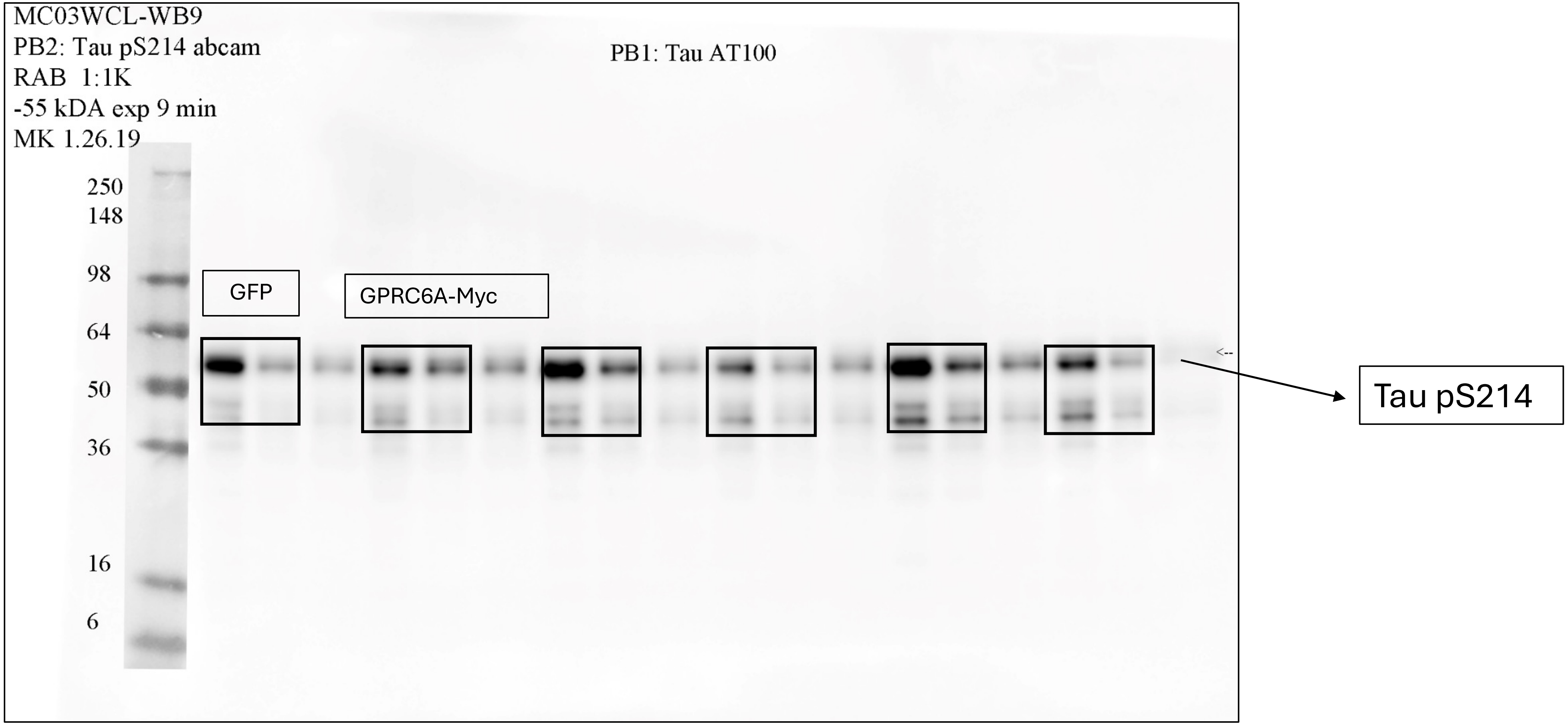

Tau pS214, Fig 7 I

Fig 7 MK-03-WB6

Added data for editor/reviewers but not included in the paper

Anti-GSK3 (alpha + beta) (phospho Y216 + Y279) antibody

KDa

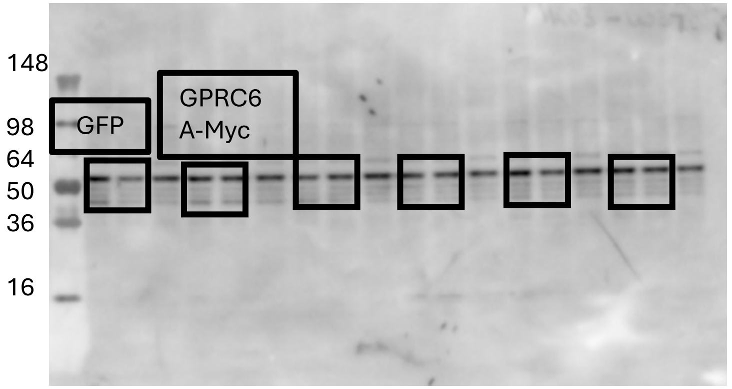

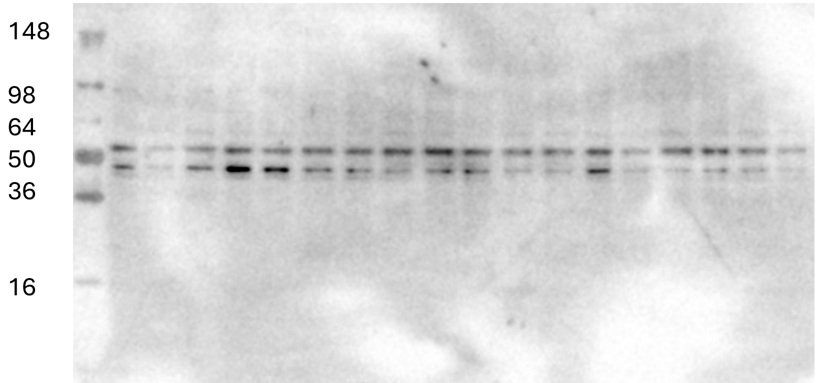

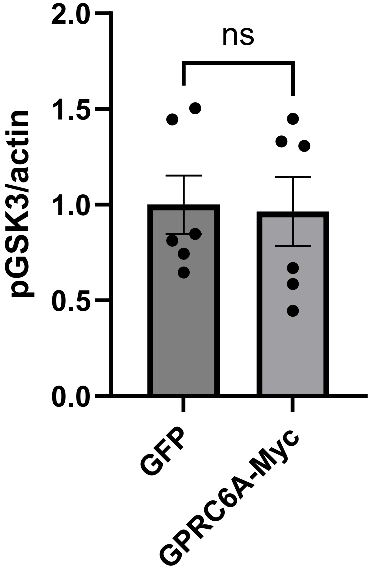

P-GSK3α+β
Rabbit 1:1000
Abcam ab4797
47Kd & 51Kd

B-actin
Mouse 1:50K
Sigma
42KD

One-way Anova, Fischer's LSD as post hoc analysis

Figure 8 Raw Blots

JC04c-WB1 RAW

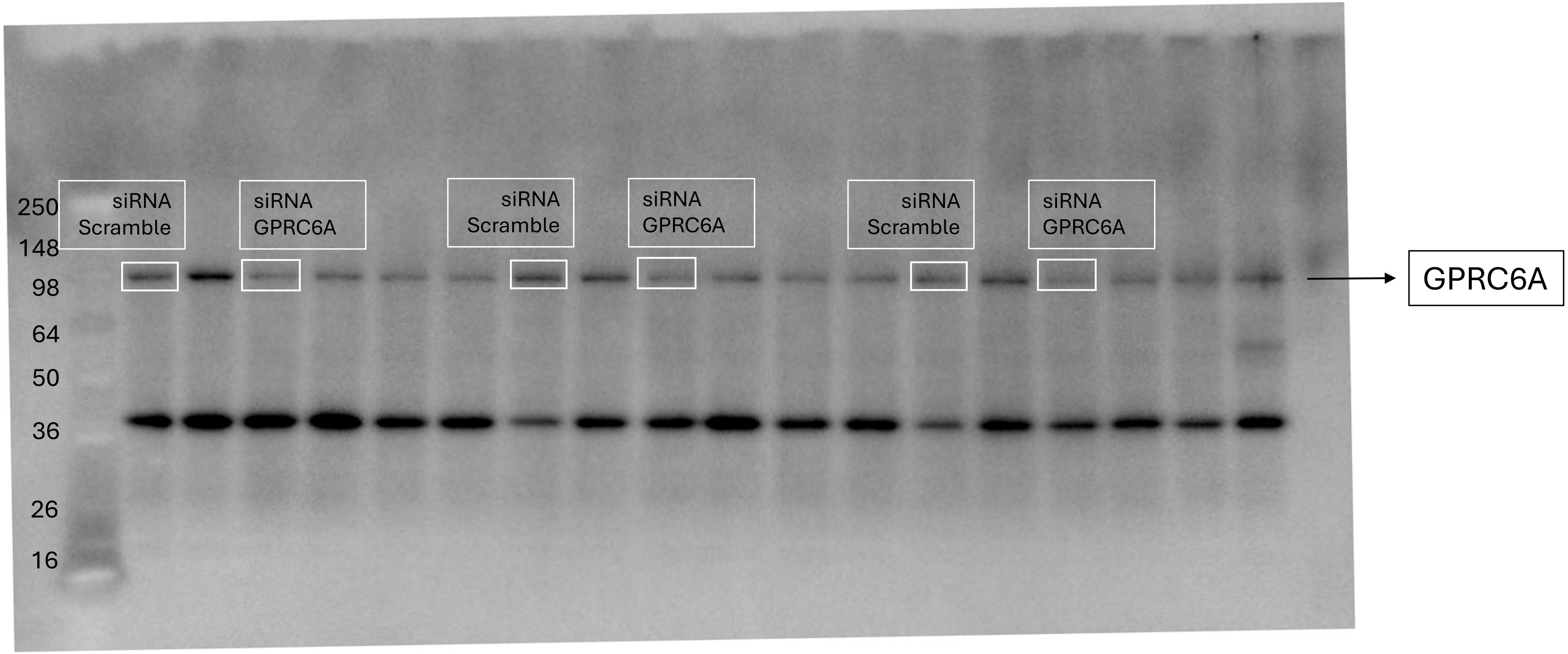

GPRC6A, Fig 8 A

JC04c-WB5 RAW

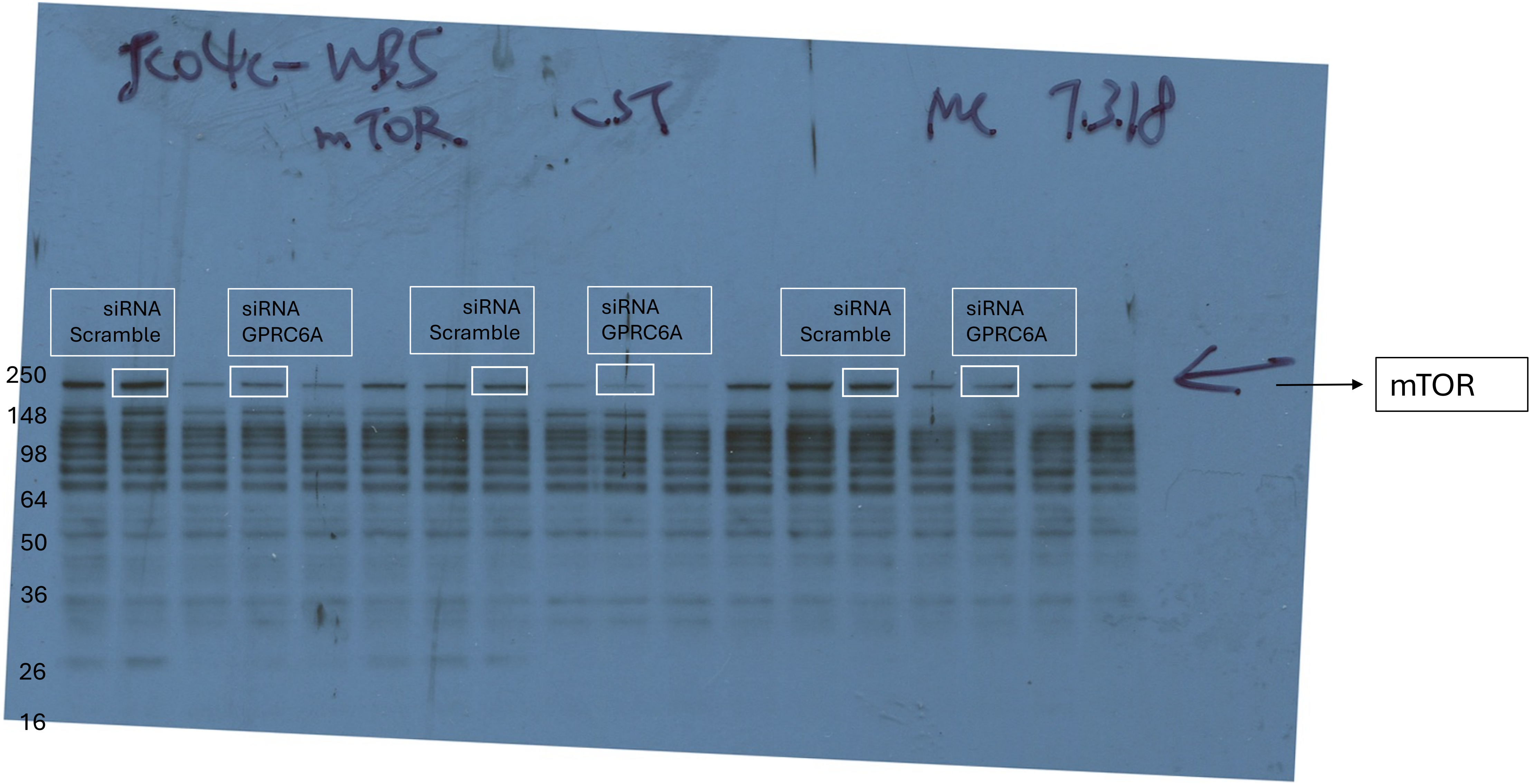

mTOR, Fig 8 A

JC04c-WB7 RAW

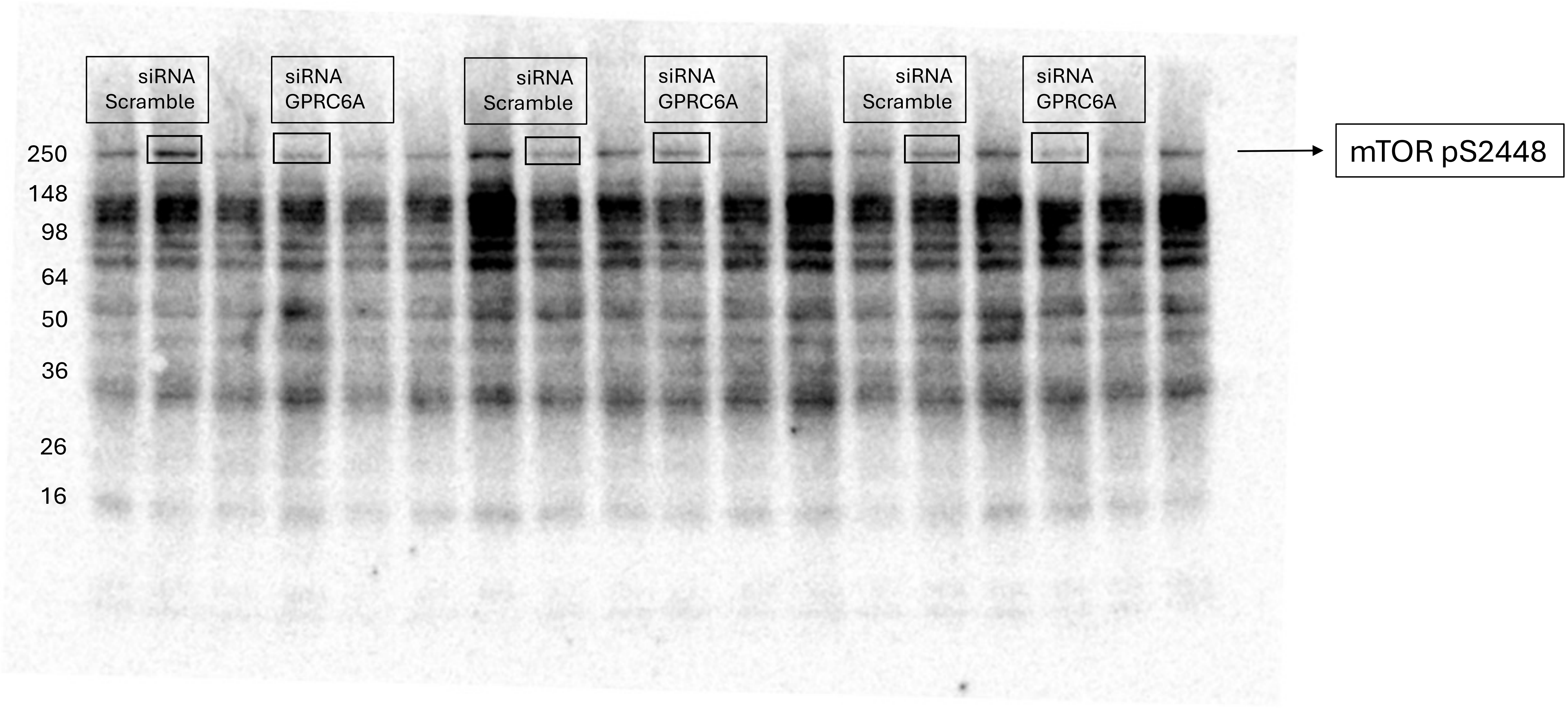

mTOR pS2448, Fig 8 A

JC04c-WB2 RAW

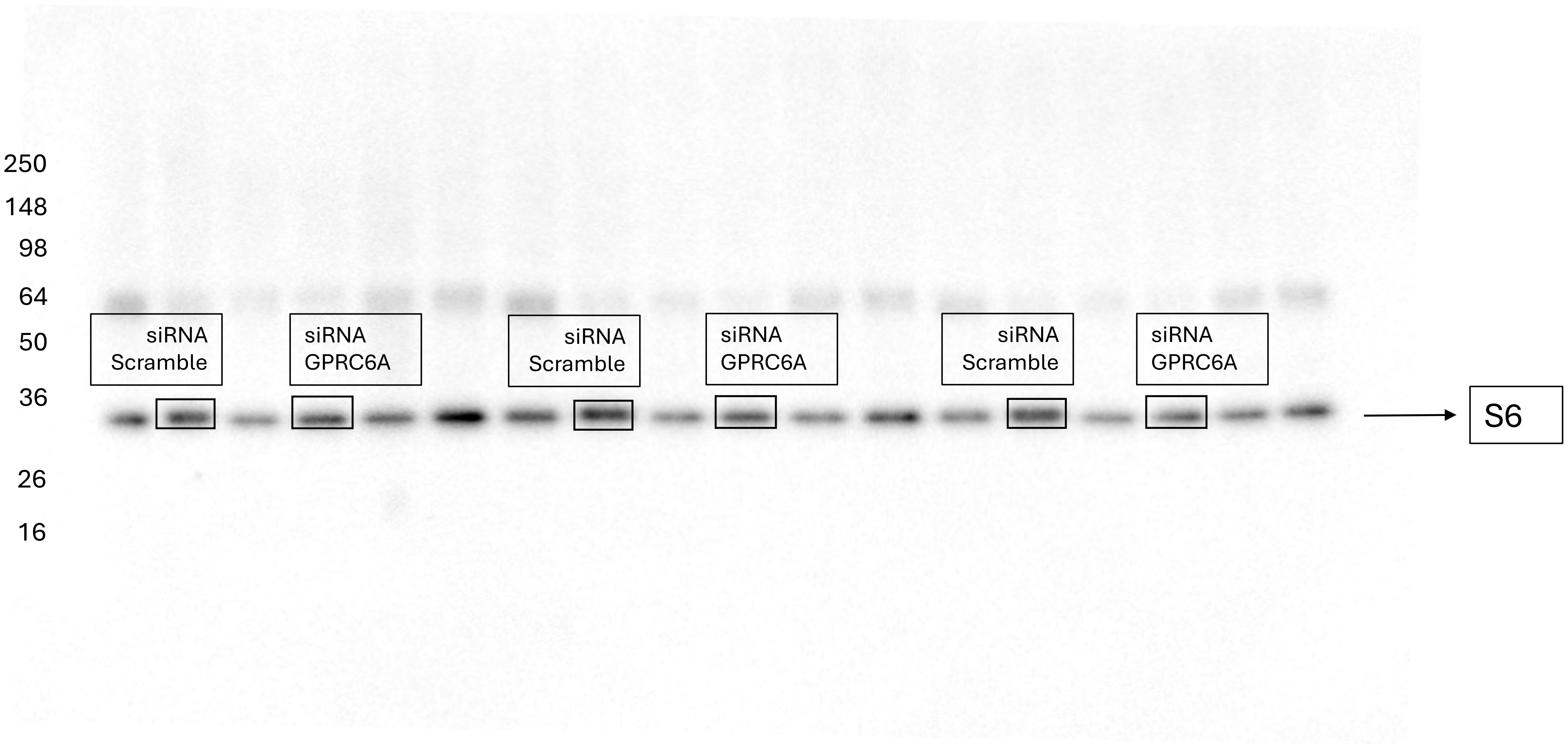

S6, Fig 8 A

JC04c-WB5 RAW

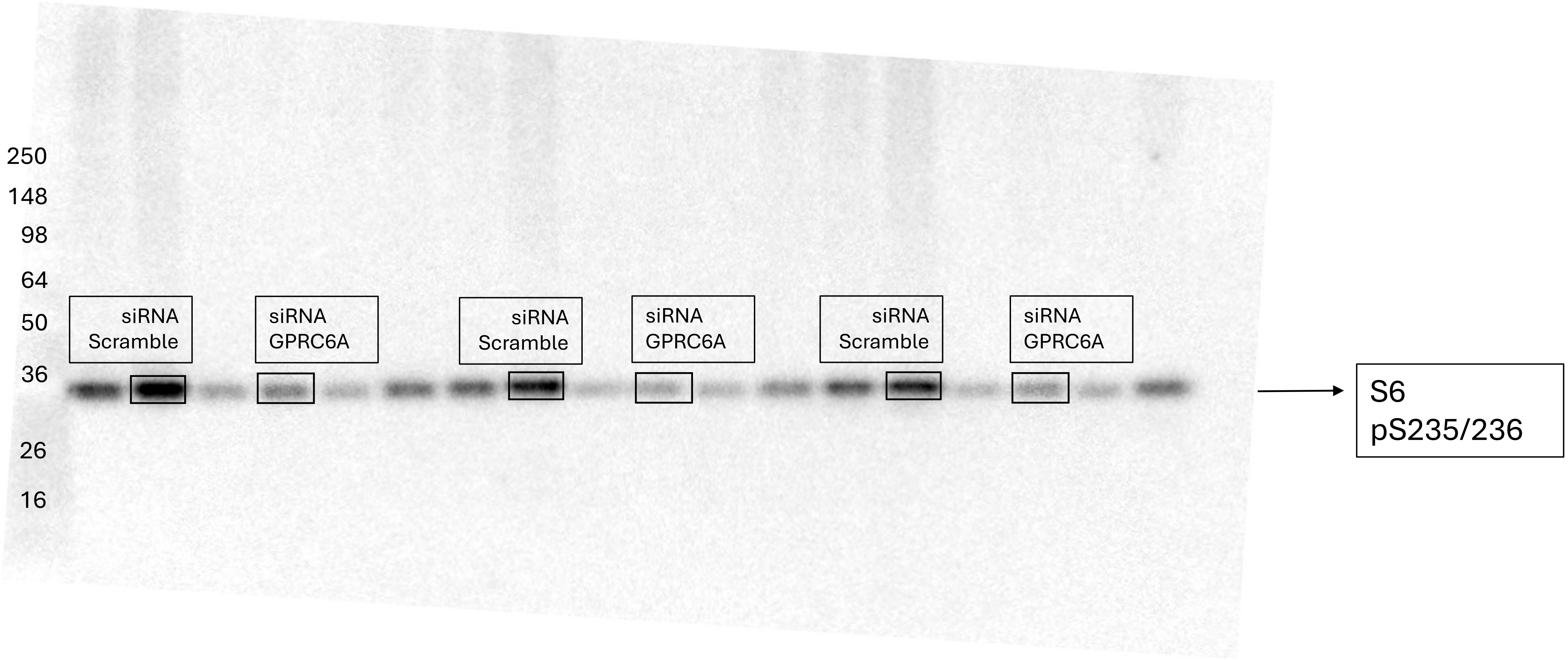

S6 pS235/236, Fig 8 A

JC04c-WB1 RAW

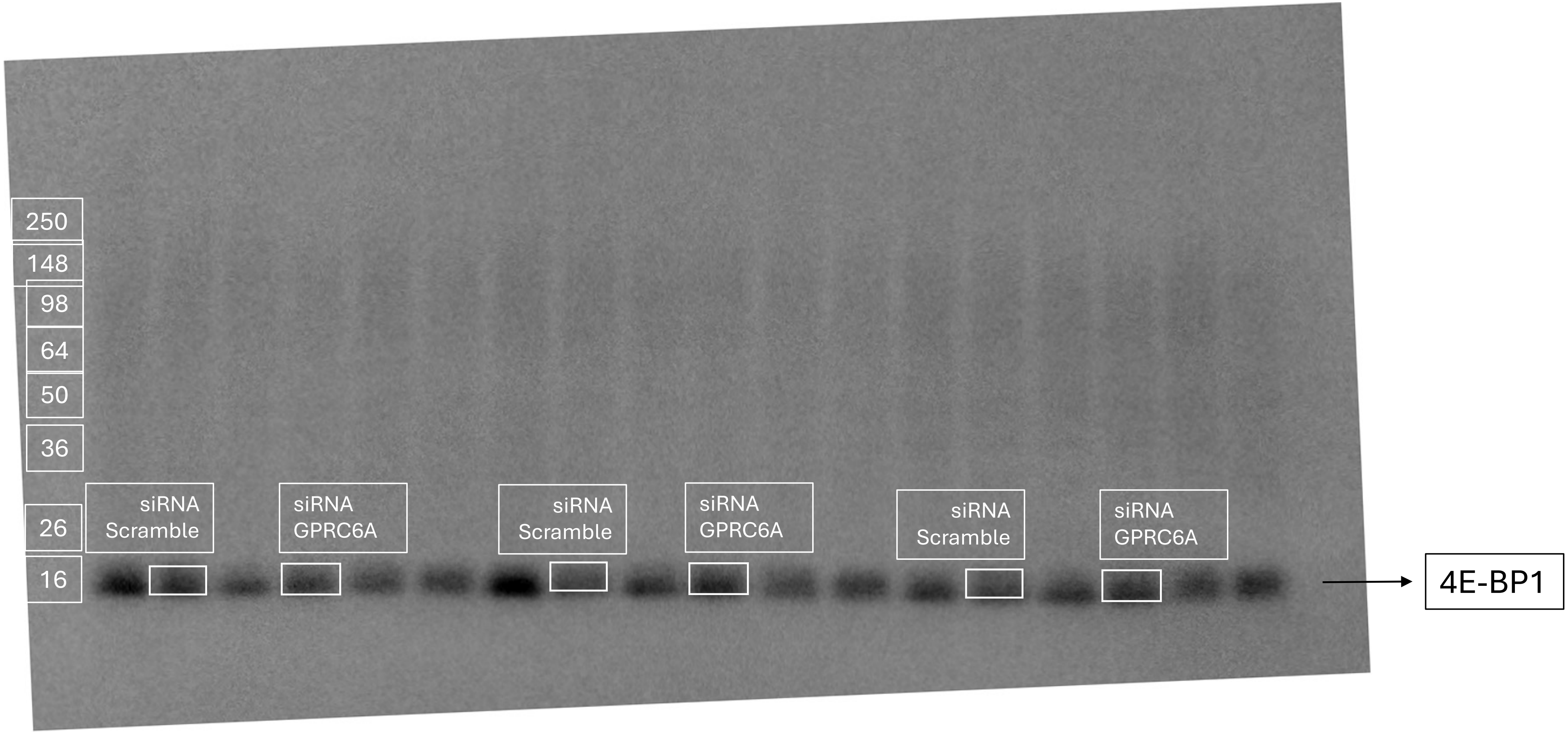

4E-BP1, Fig 8 A

JC04c-WB5 RAW

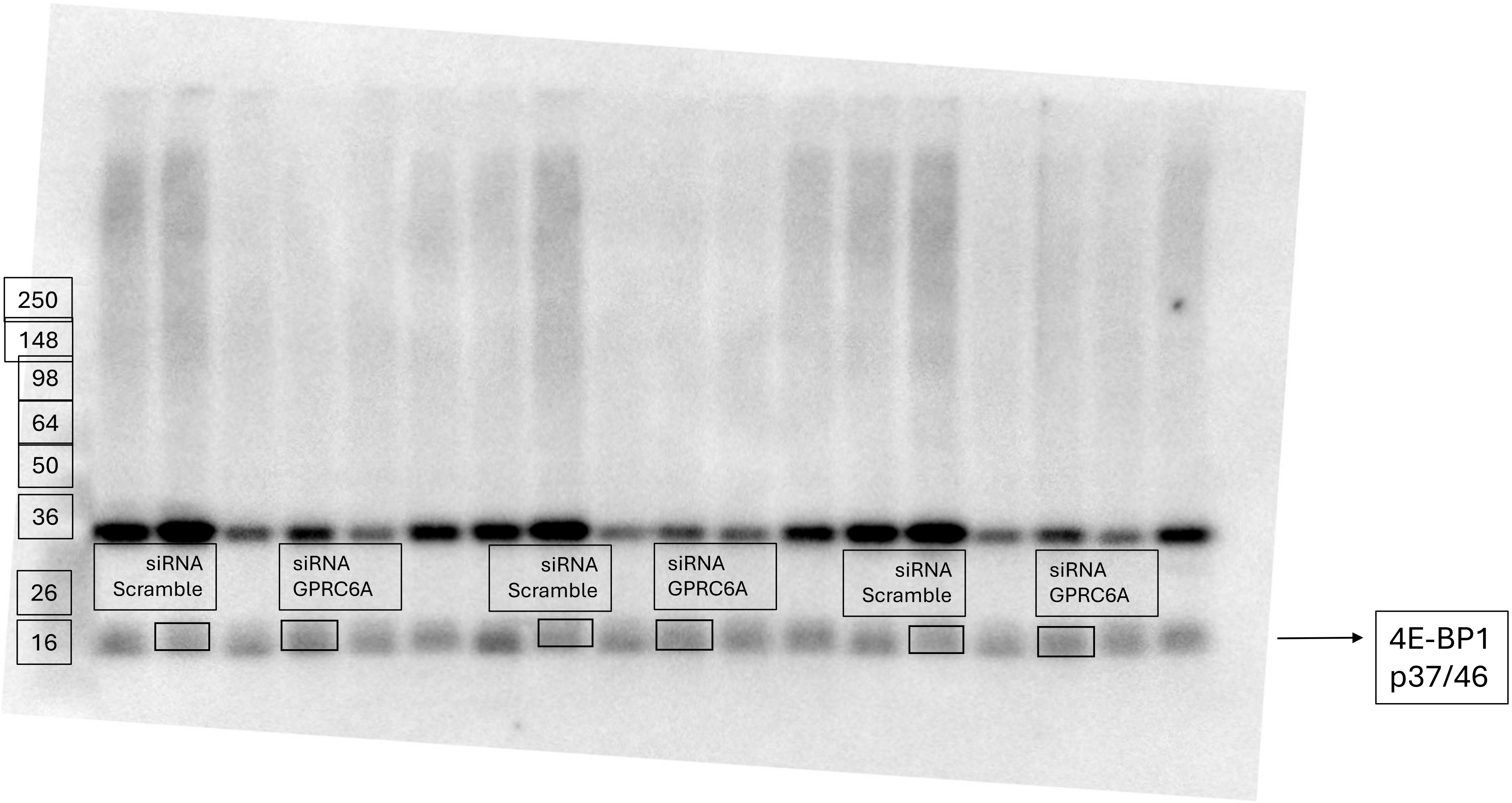

4E-BP1 pT37/46, Fig 8 A

JC04c-WB1 RAW

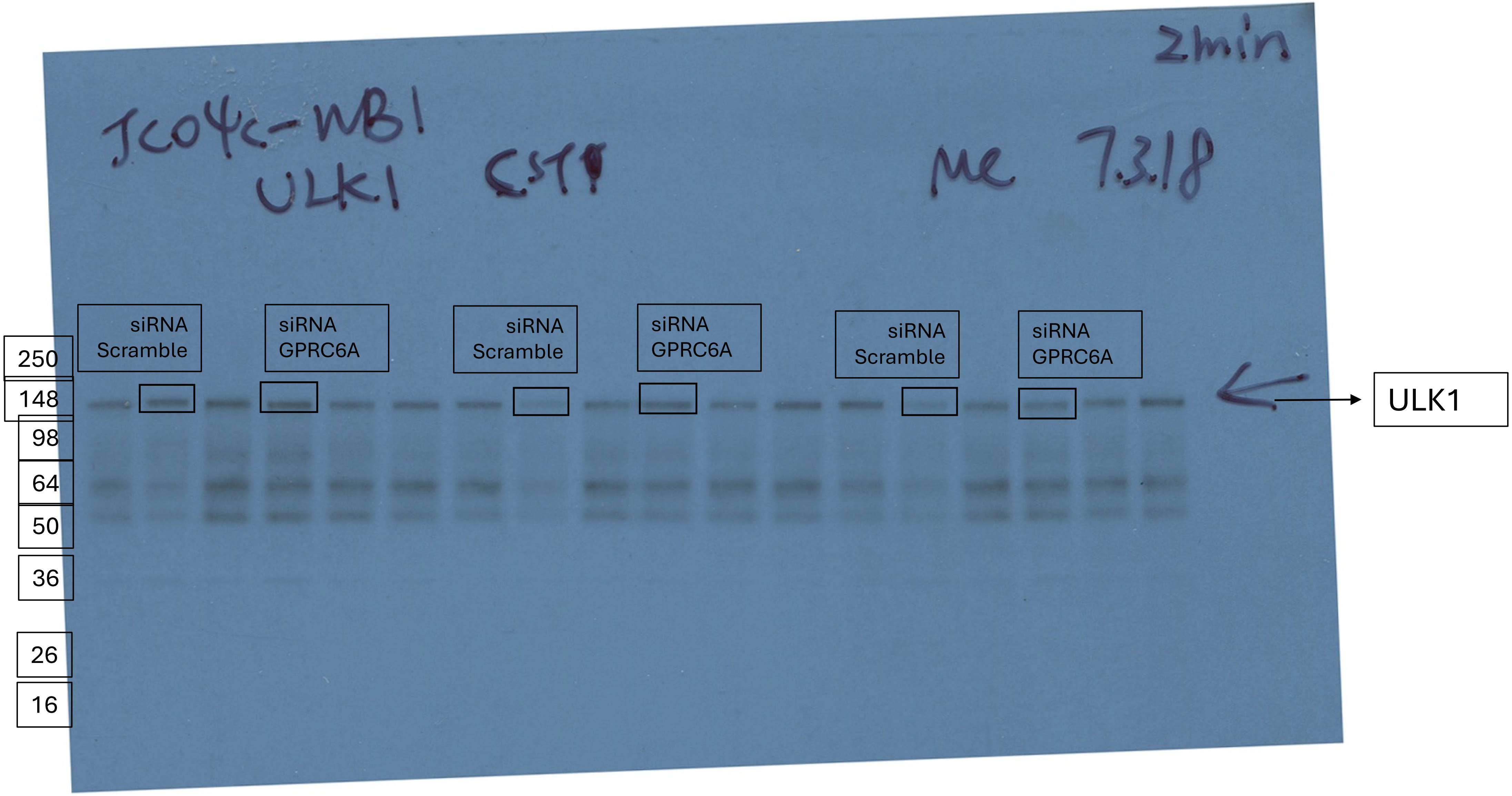

ULK1, Fig 8 A

JC04c-WB5 RAW

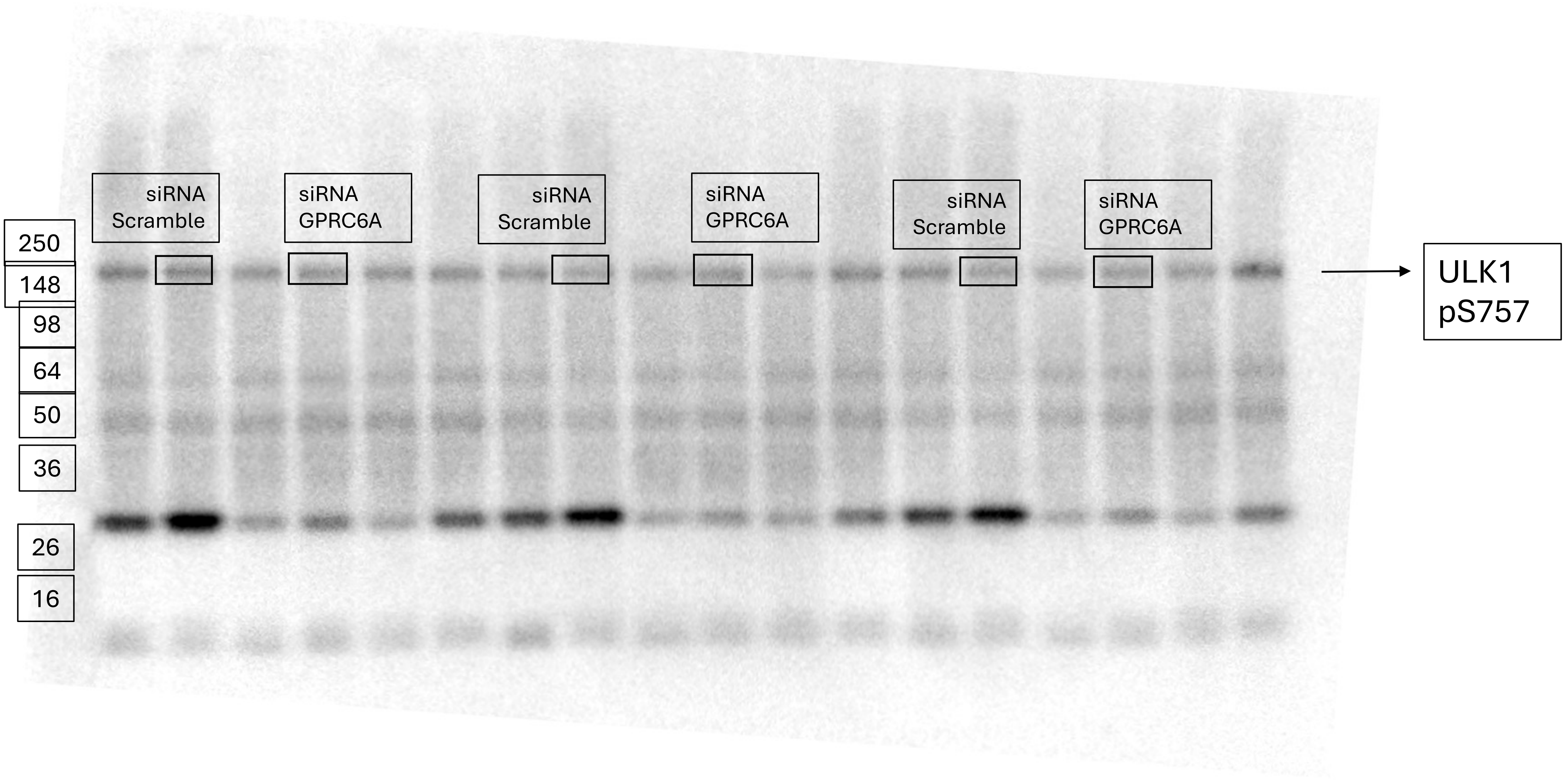

ULK1 pS757, Fig 8 A

JC04c-WB7 RAW

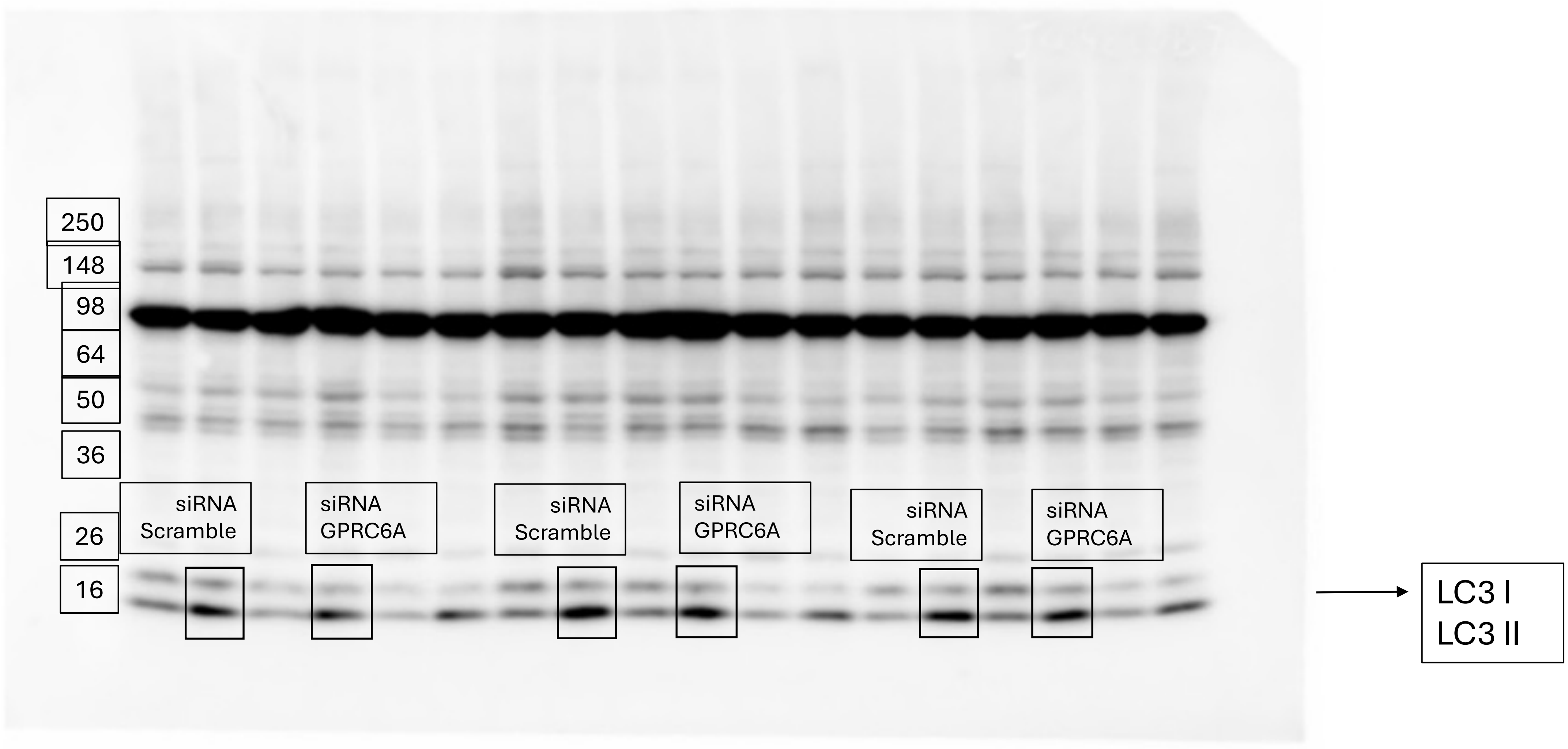

LC3, Fig 8 A

JC04c-WB2 RAW

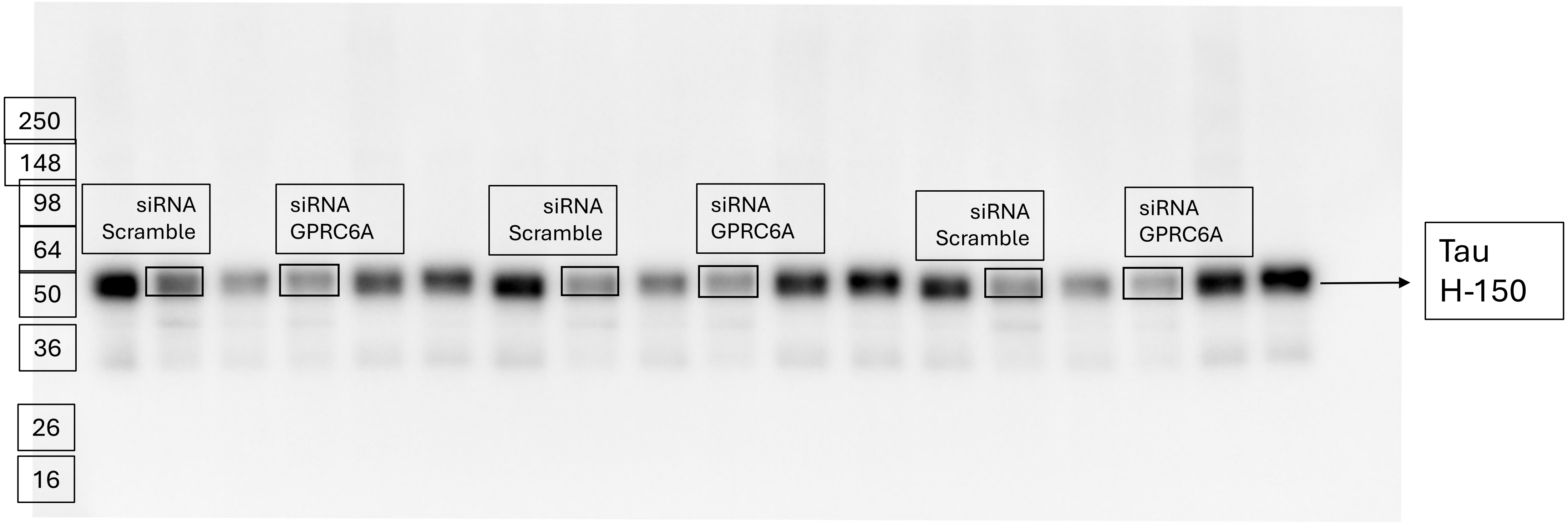

LC3, Fig 8 A

JC04c-WB8 RAW

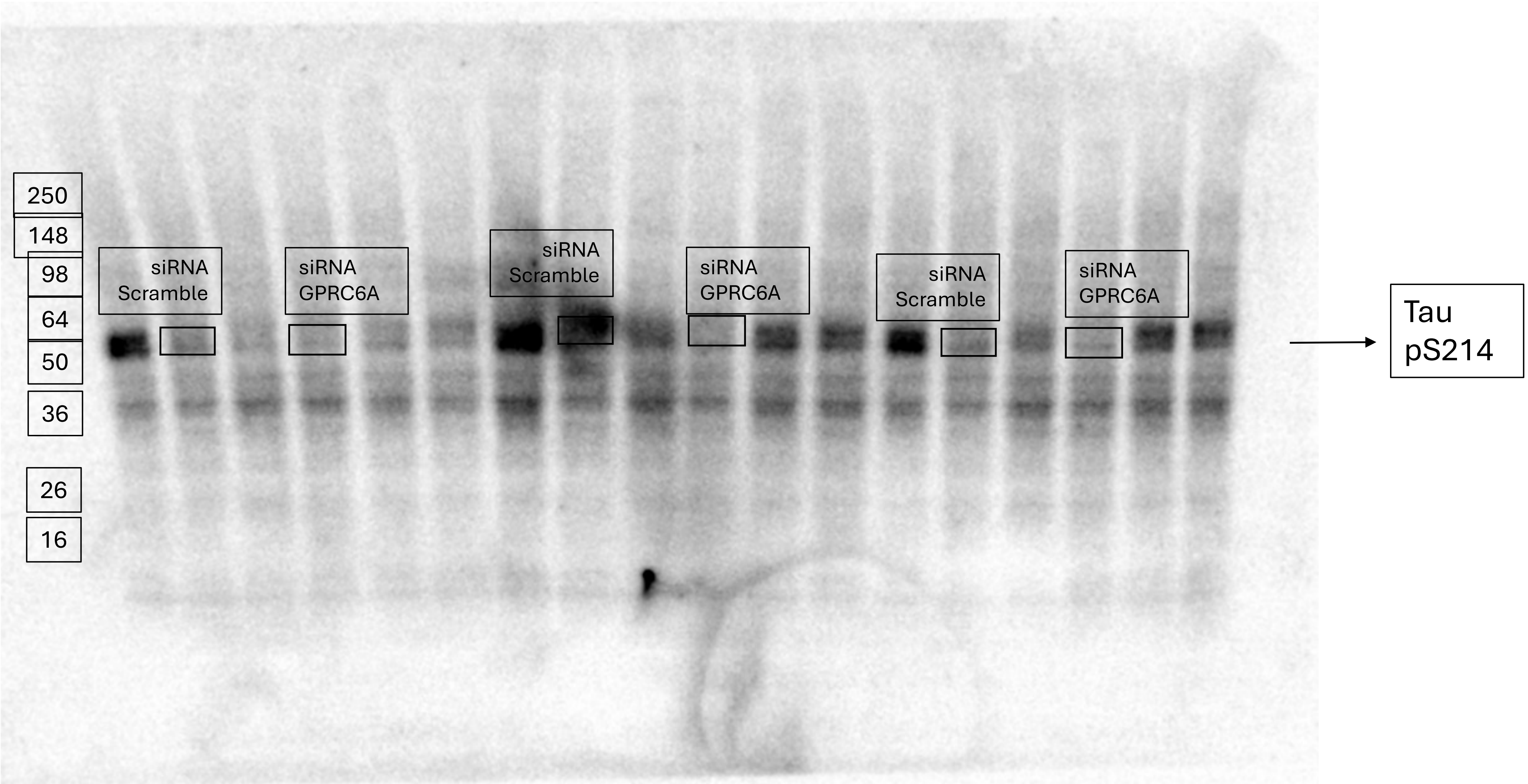

Tau pS214, Fig 8 A

MC053-WB1 RAW

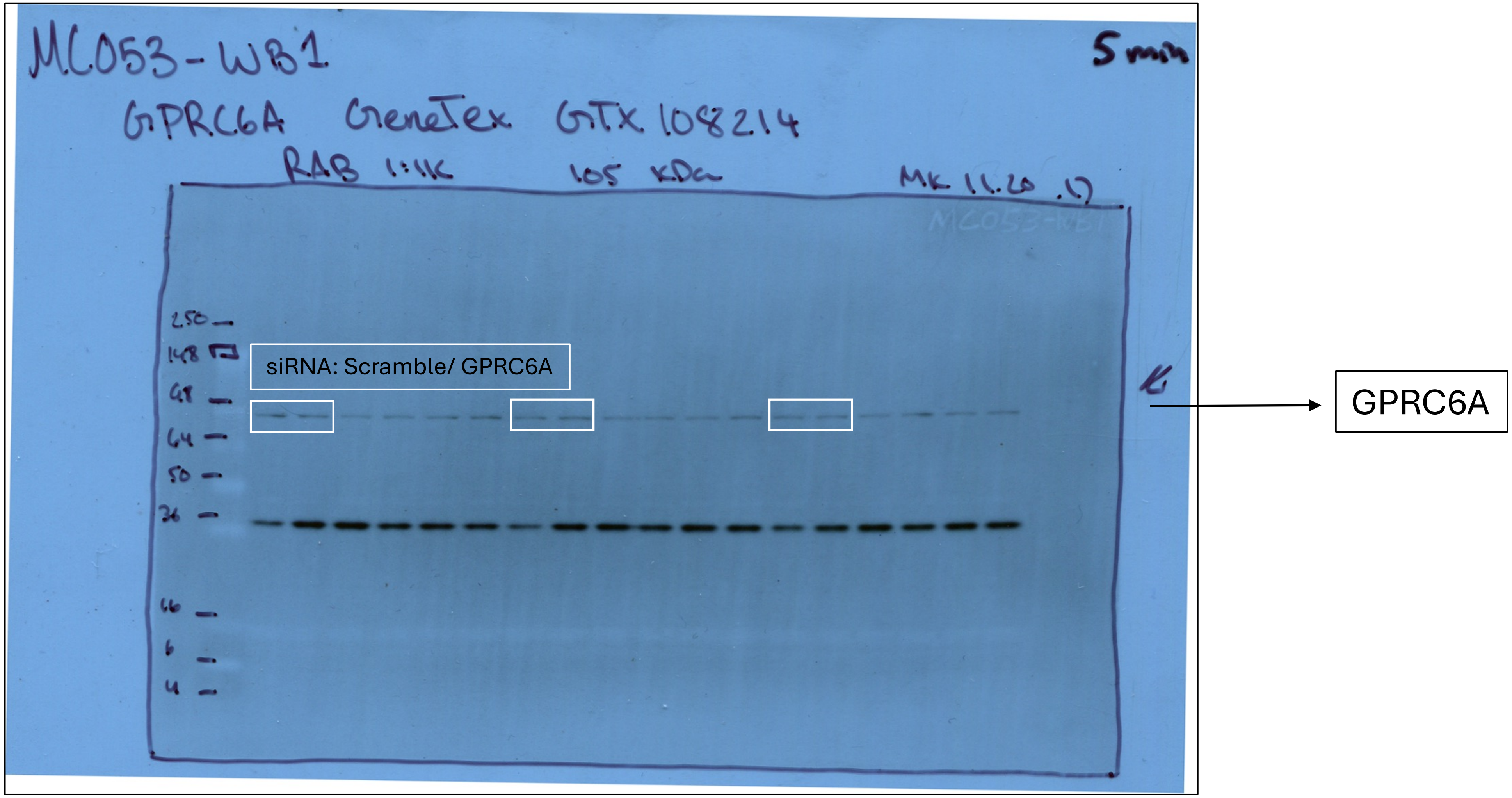

GPRC6A

GPRC6A, Fig 8 I

MC053 WB1 RAW

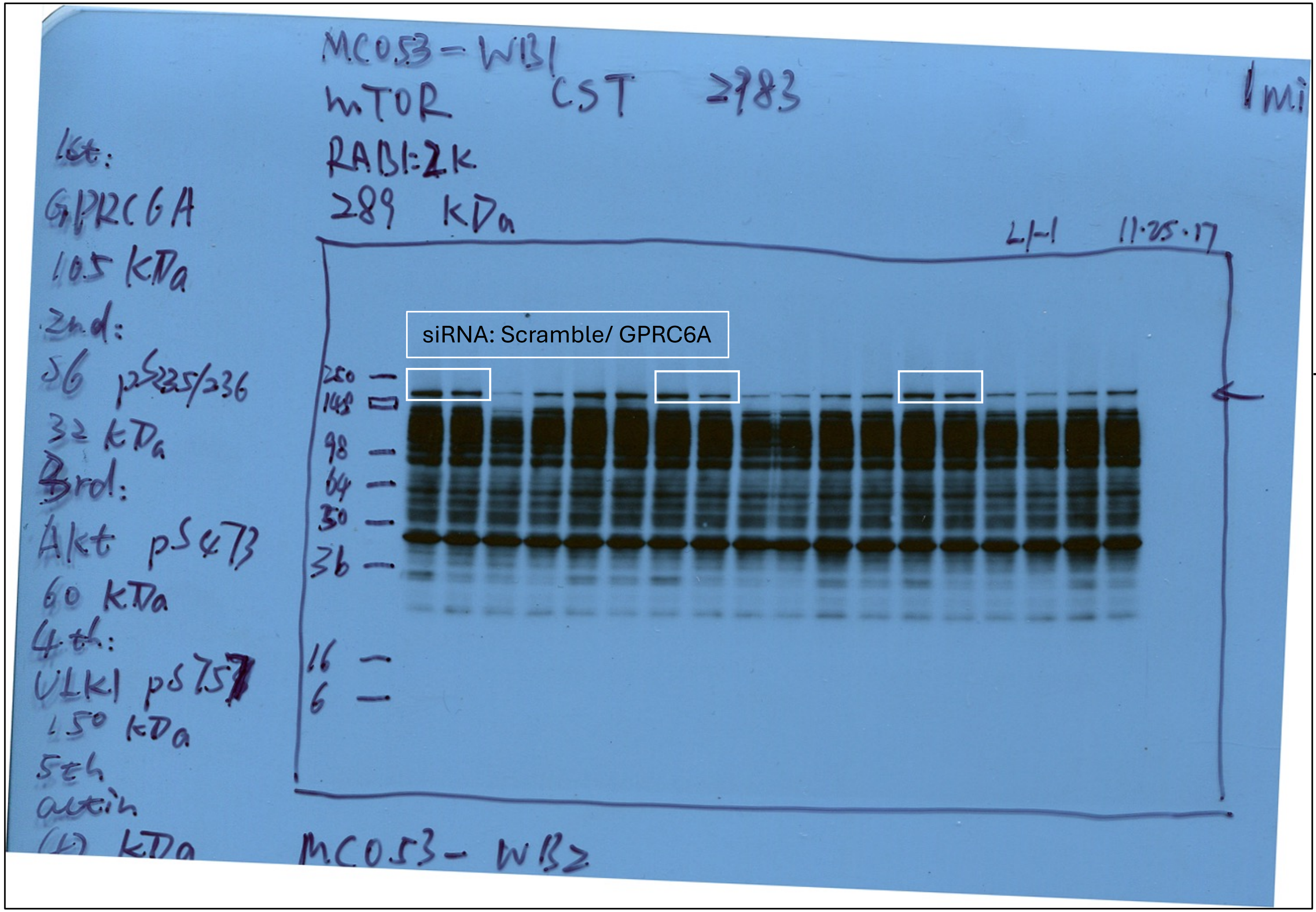

mTOR

mTOR, Fig 8 I

MC053 WB2 RAW

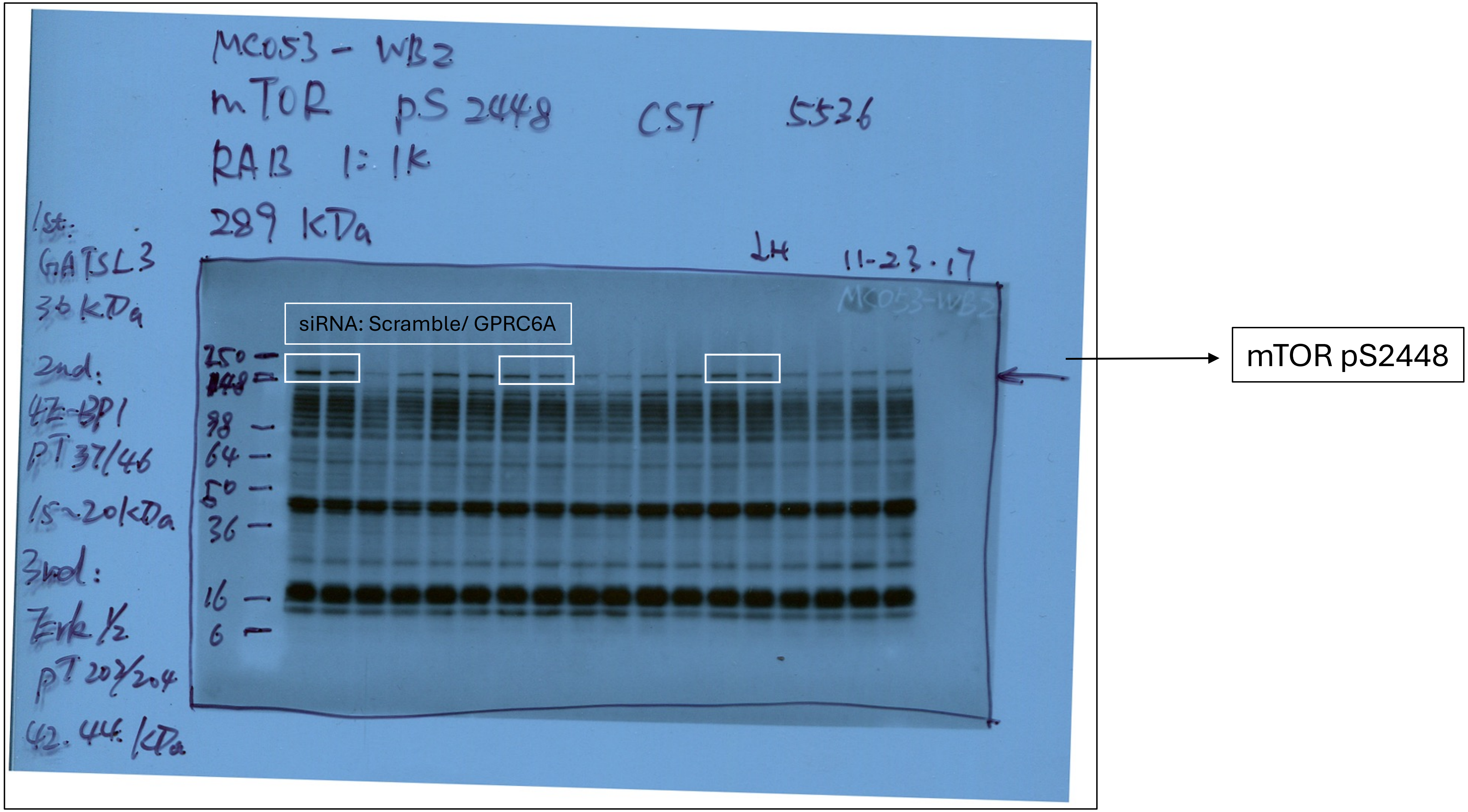

mTOR pS2448, Fig 8 I

MC053 WB5 RAW

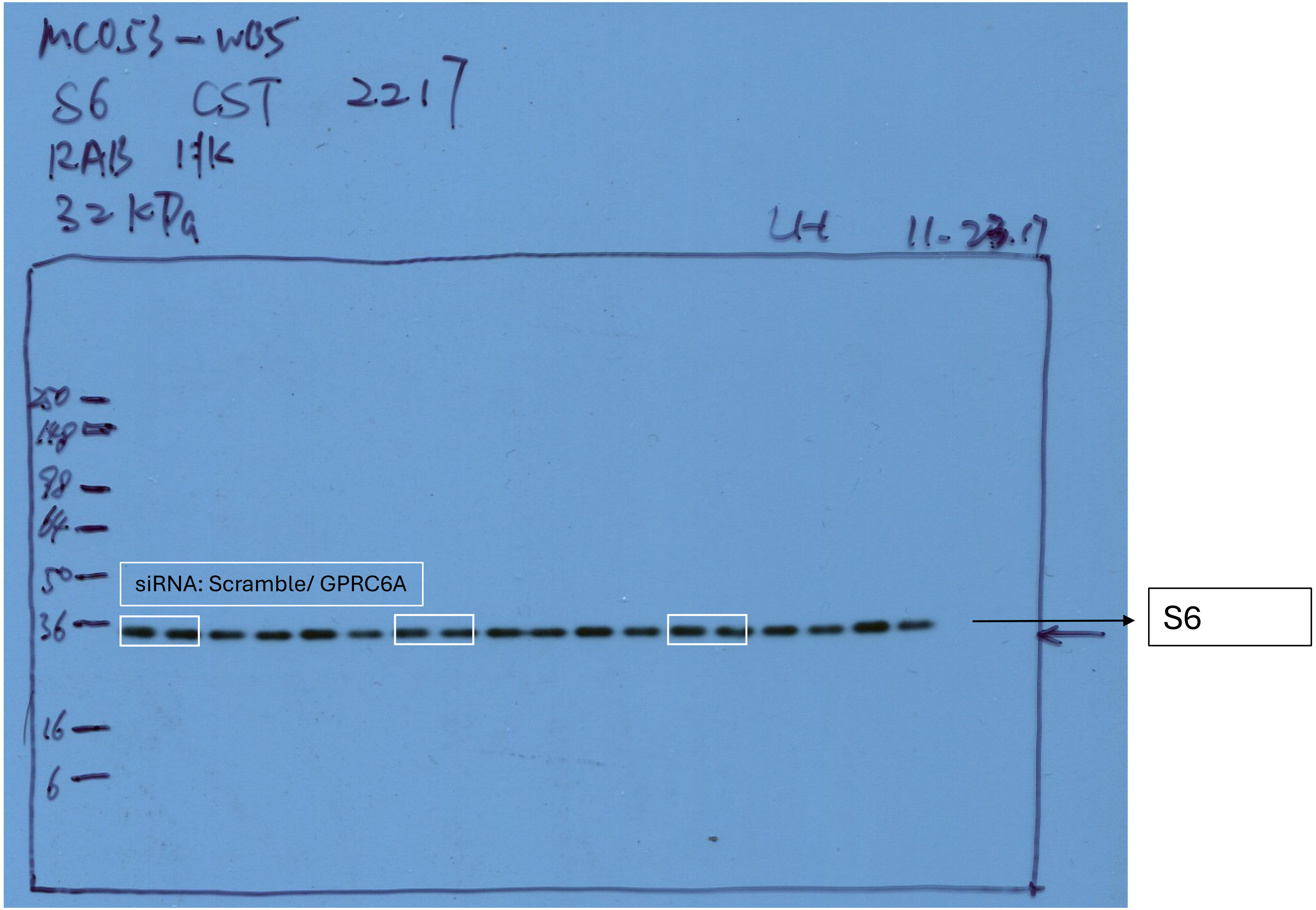

S6, Fig 8 I

MC053 WB1 RAW

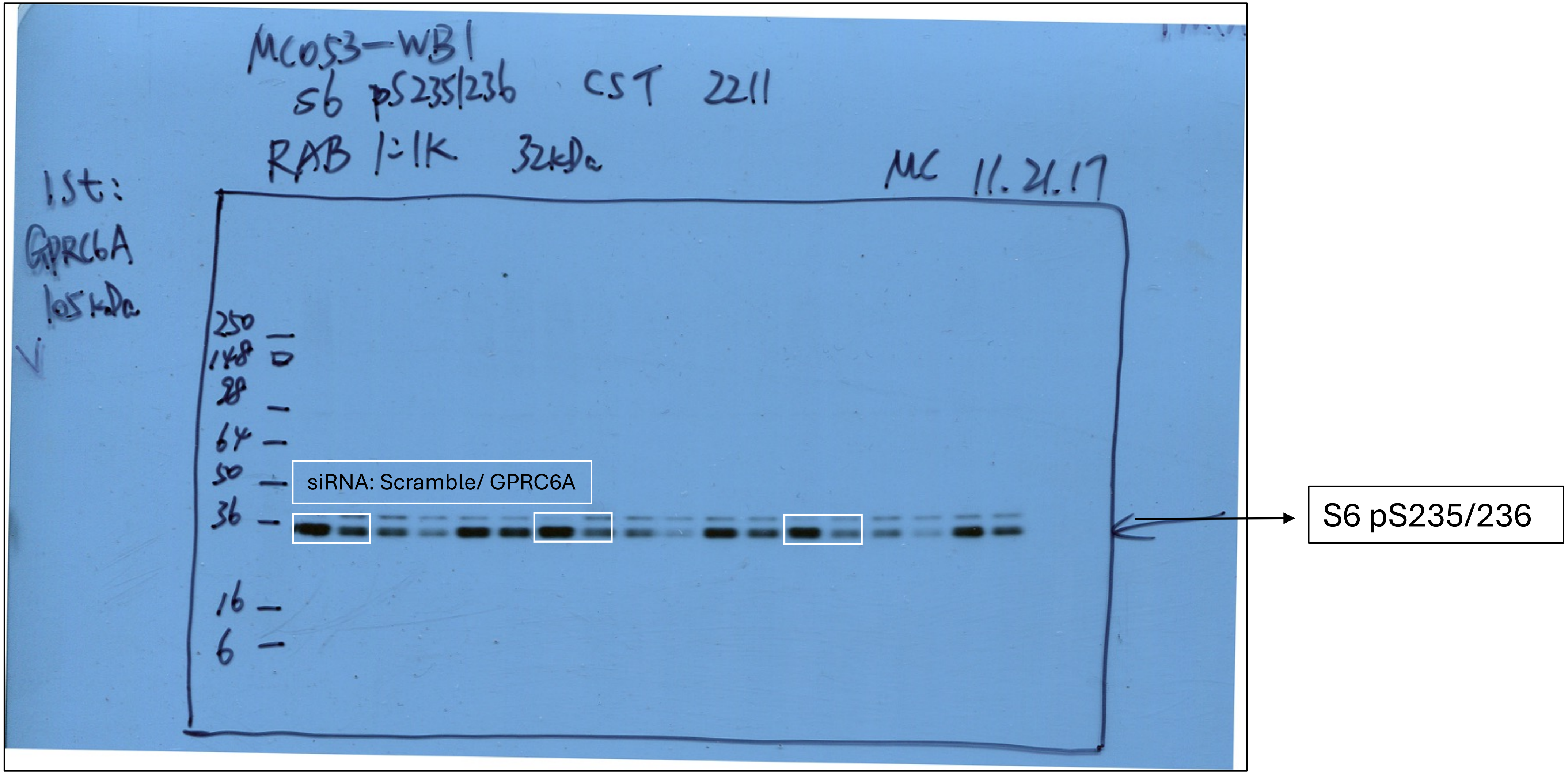

S6 pS235/236, Fig 8 I

MC054 WB4 RAW

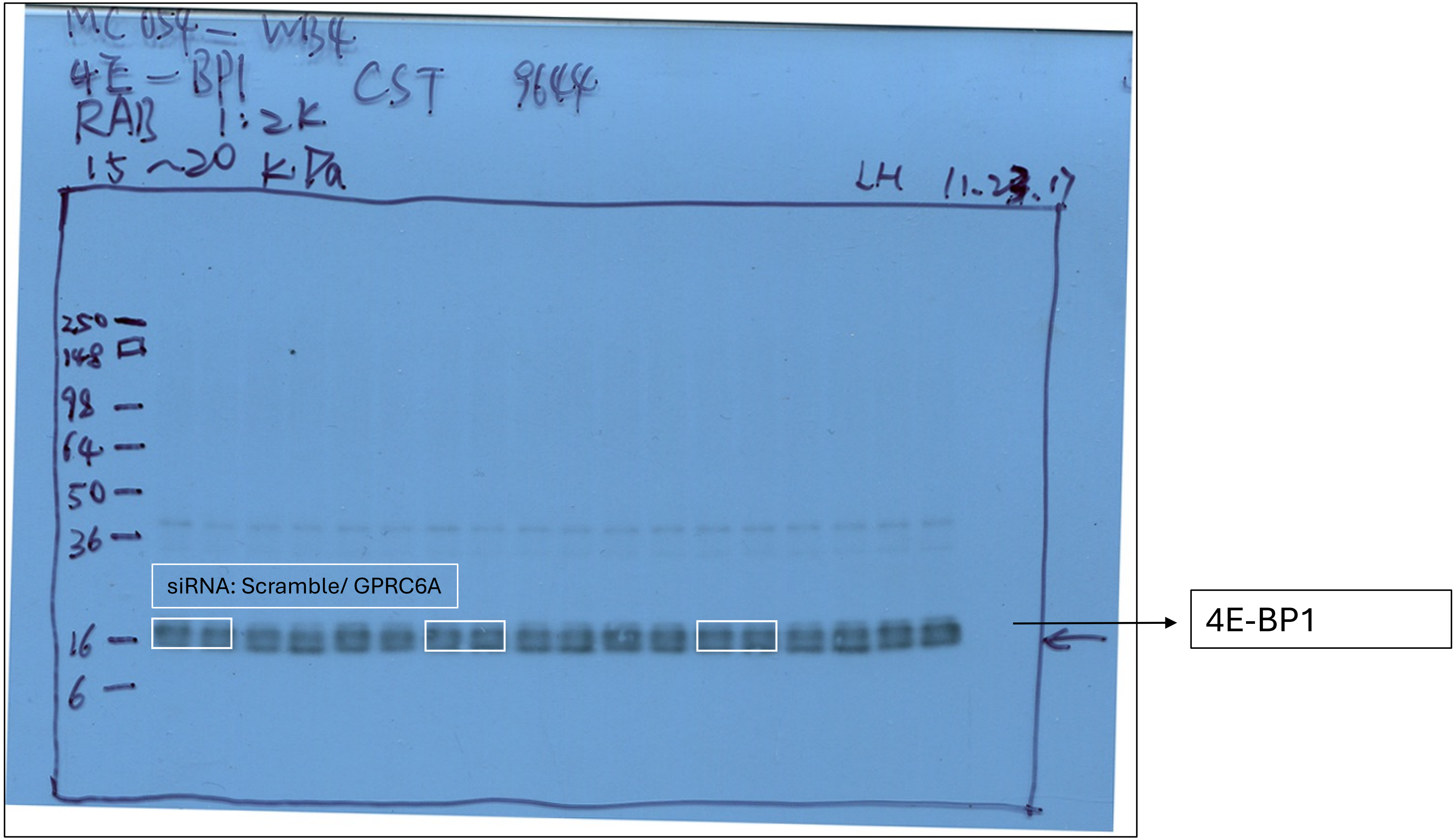

4E-BP1, Fig 8 I

MC053 WB2 RAW

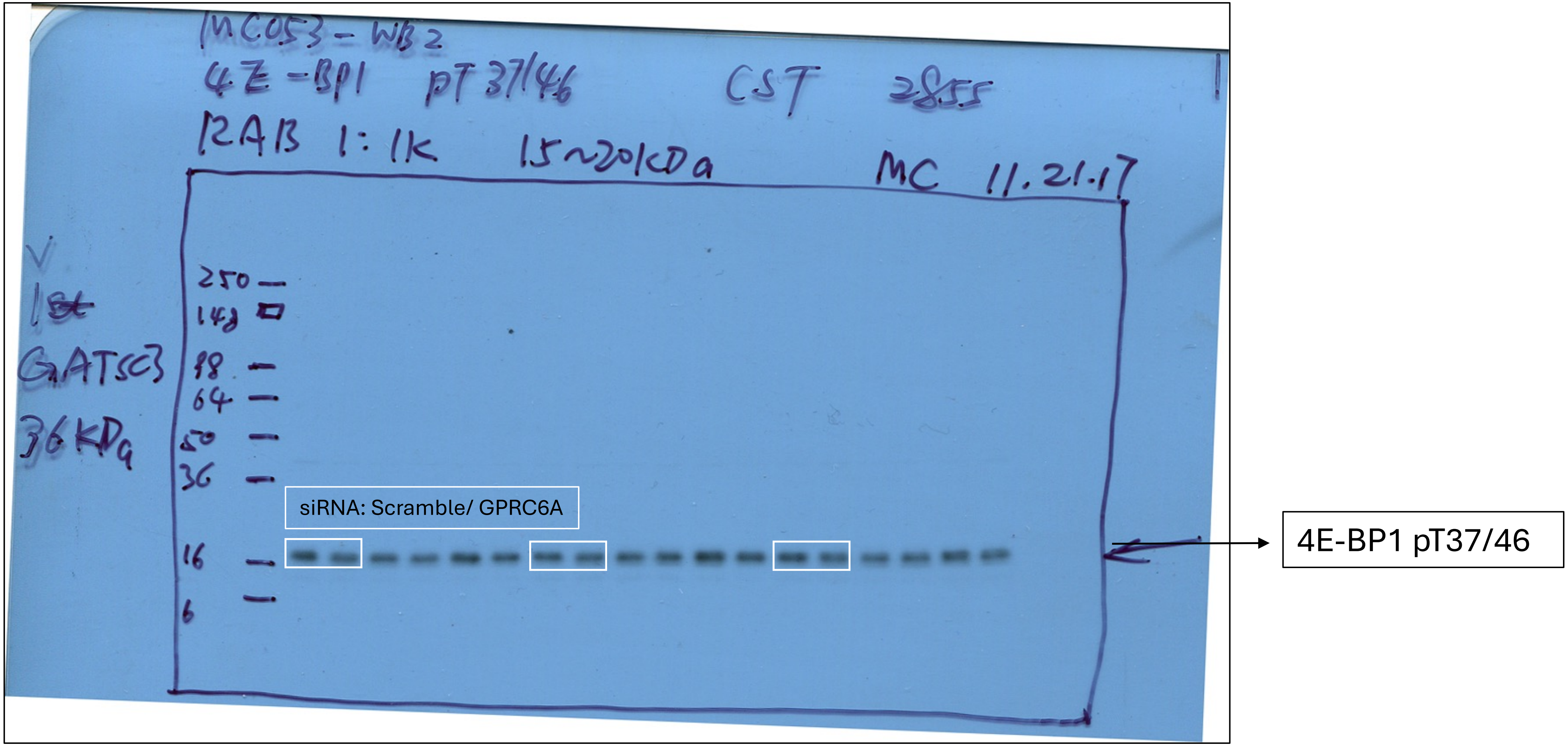

4E-BP1 pT37/46, Fig 8 I

MC053 WB2 RAW

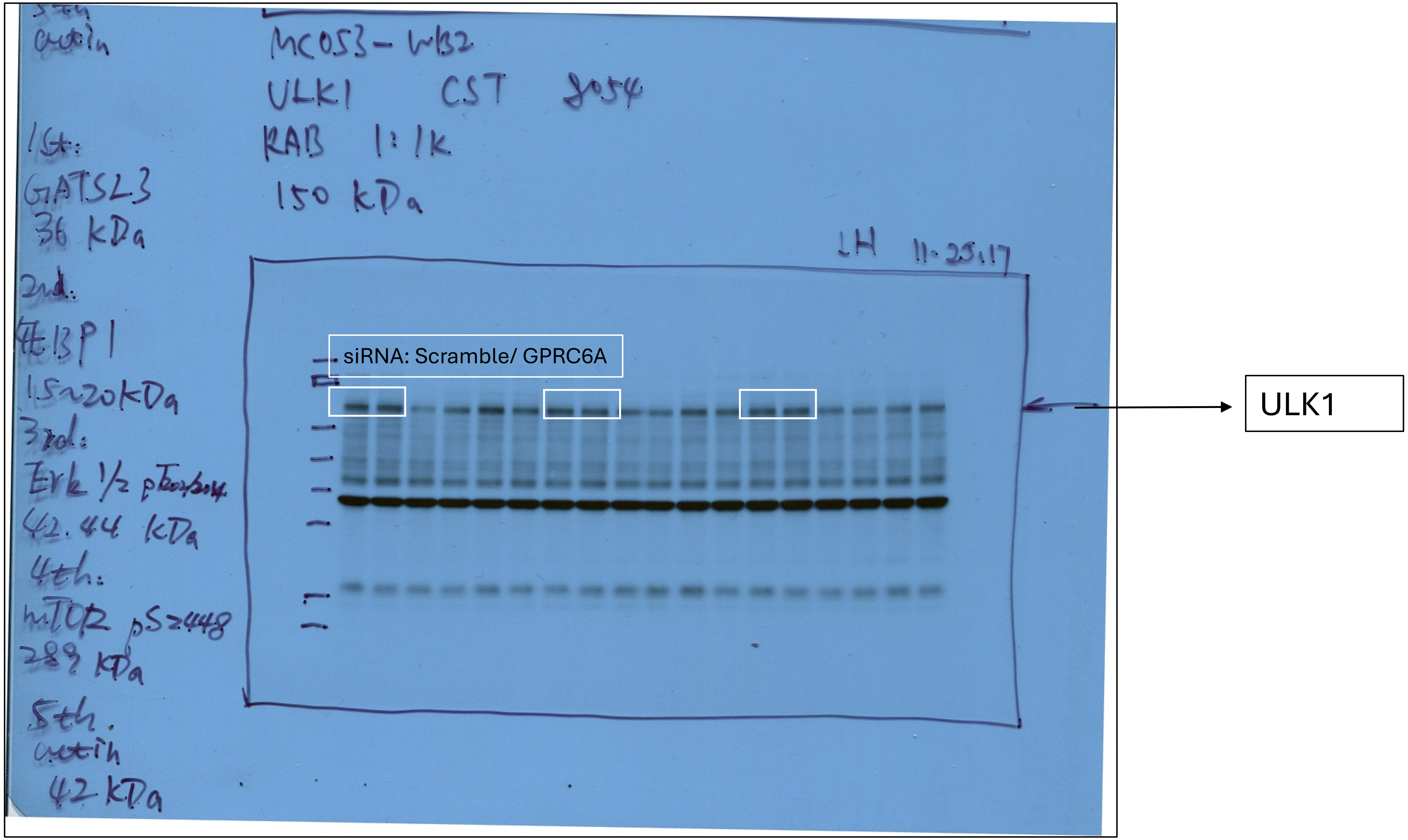

ULK1, Fig 8 I

MC053 WB1 RAW

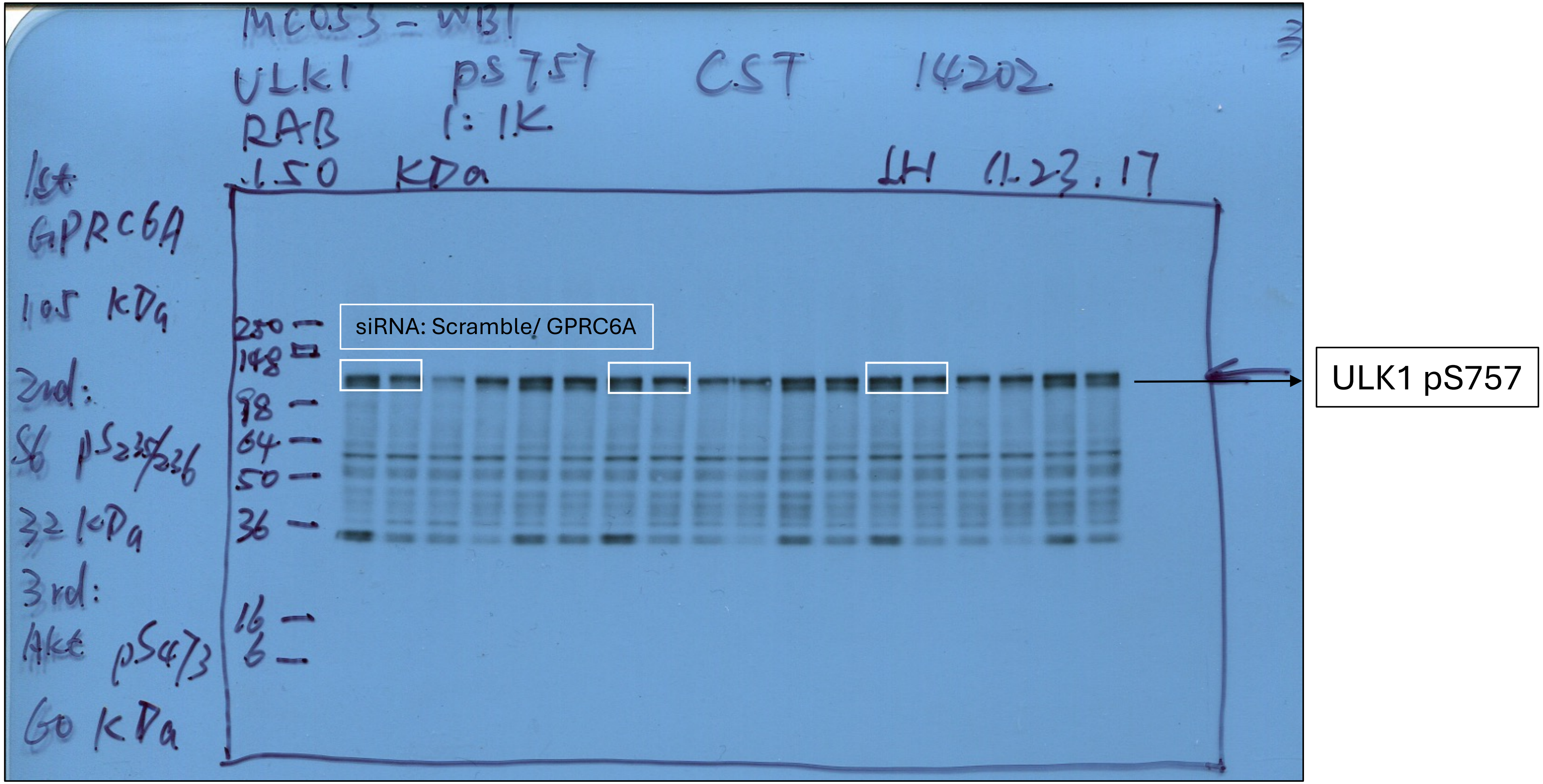

ULK1 pS757, Fig 8 I

MC053-WB6 RAW

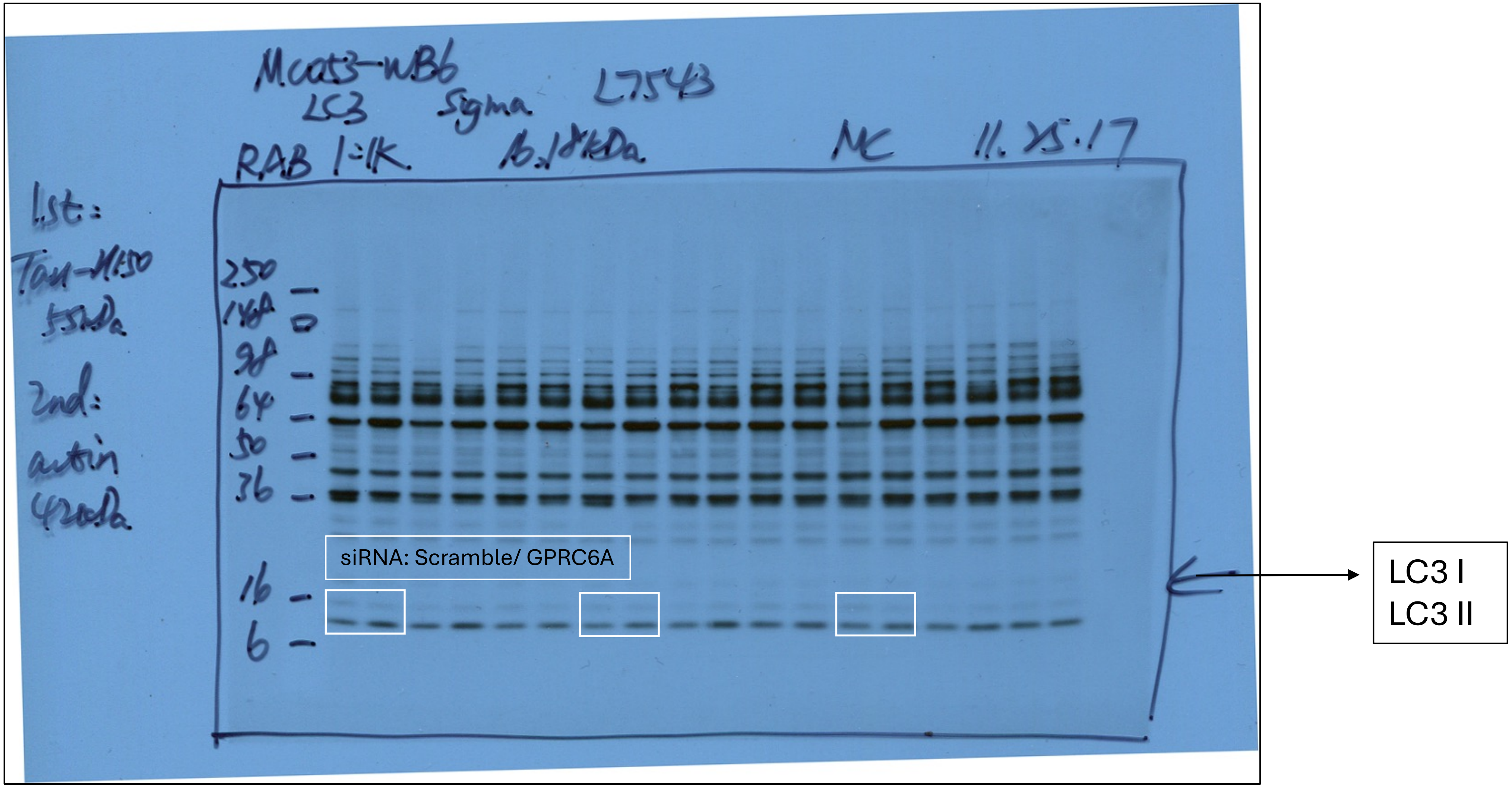

LC3 I
LC3 II

LC3, Fig 8 I

MC053 WB6 RAW

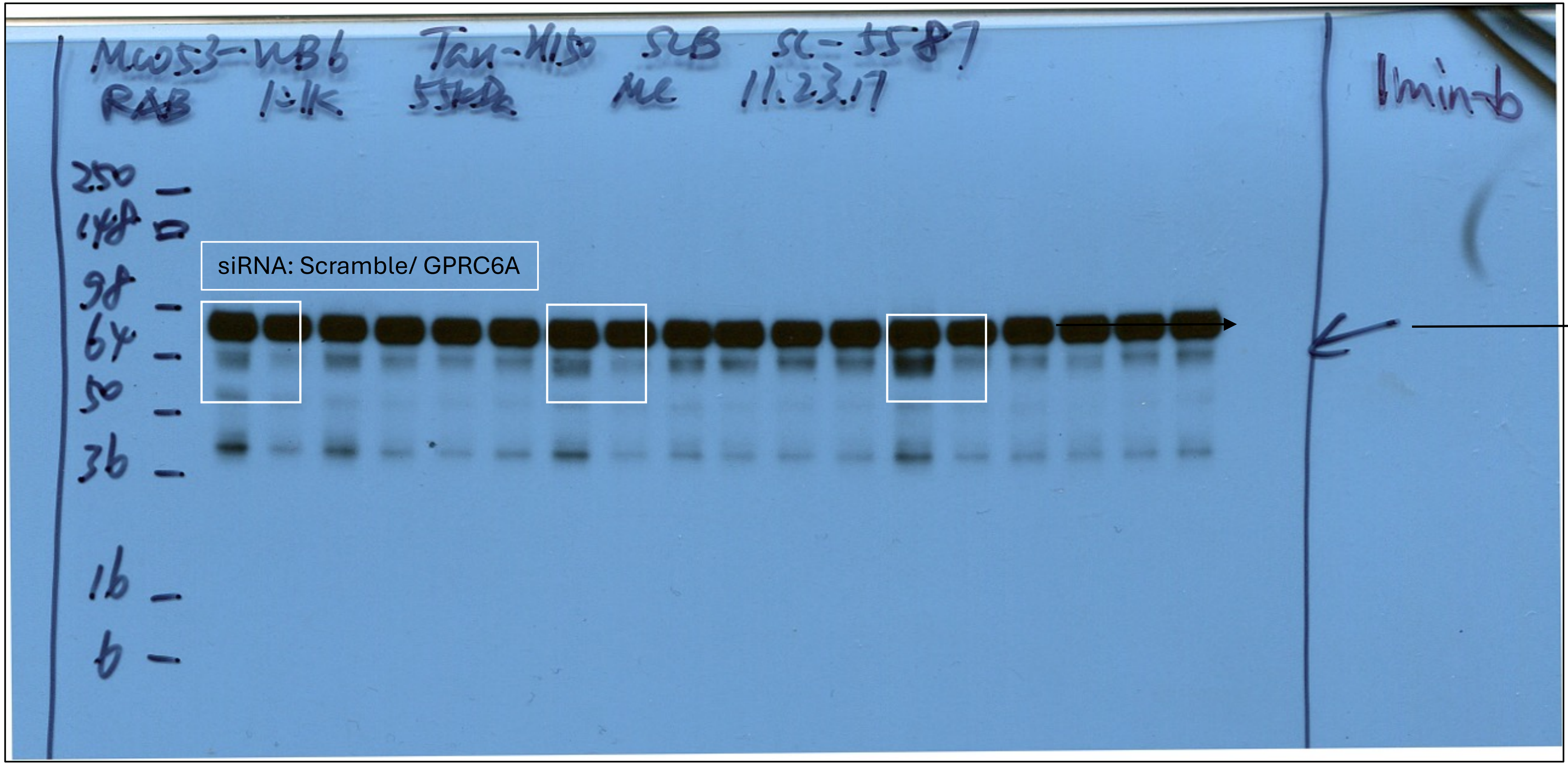

Tau (total, H150)

Tau (total, H150), Fig 8 I

MC053-WB5 RAW

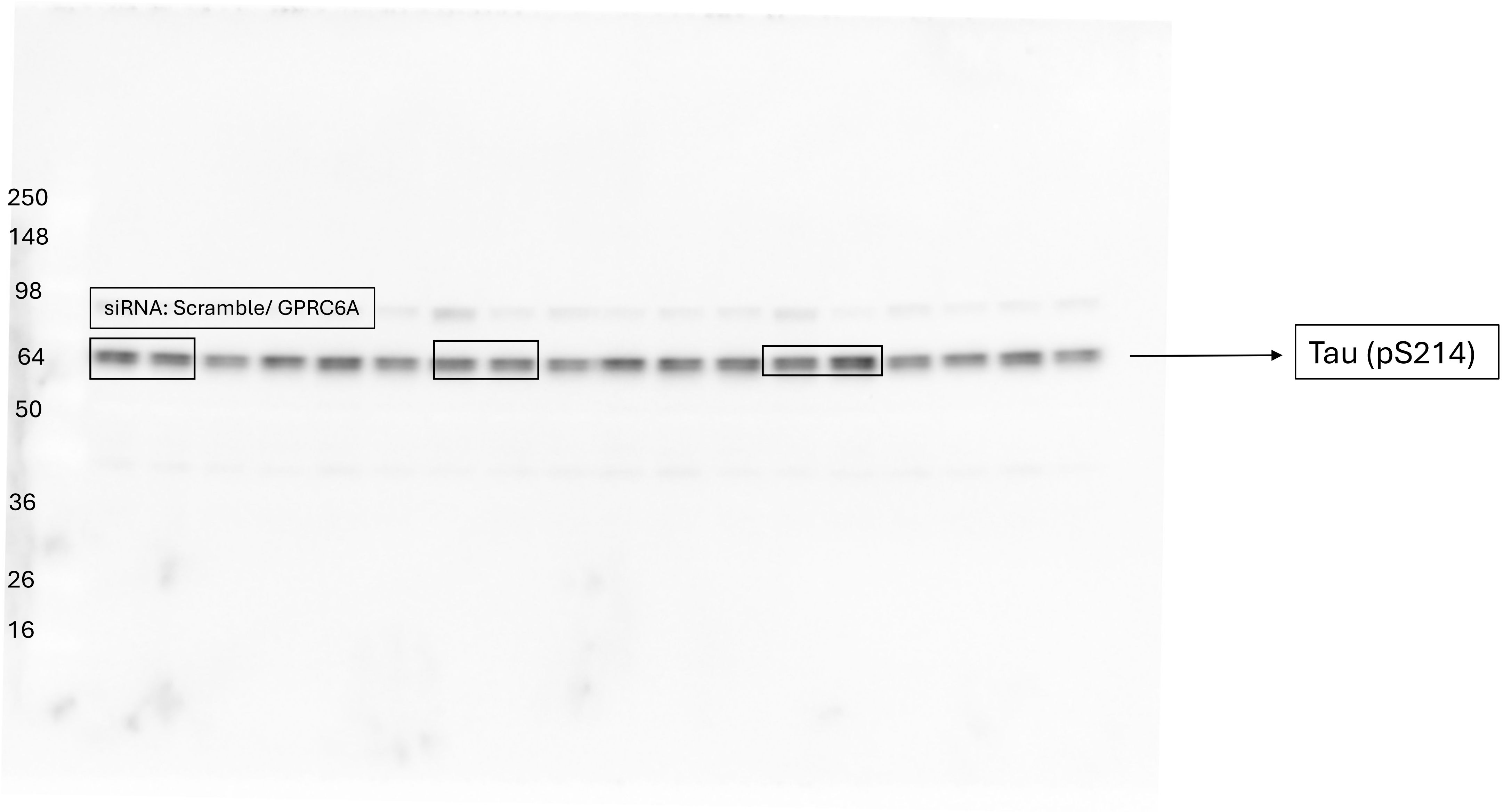

Tau (pS214), Fig 8 I

Fig 8I MC053-WB2

Anti-GSK3 (alpha + beta) (phospho Y216 + Y279) antibody

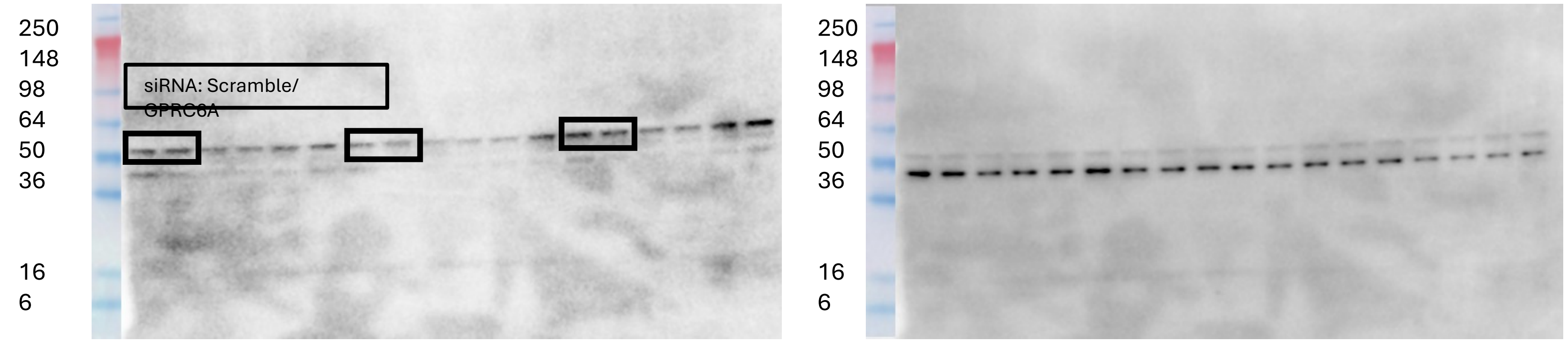

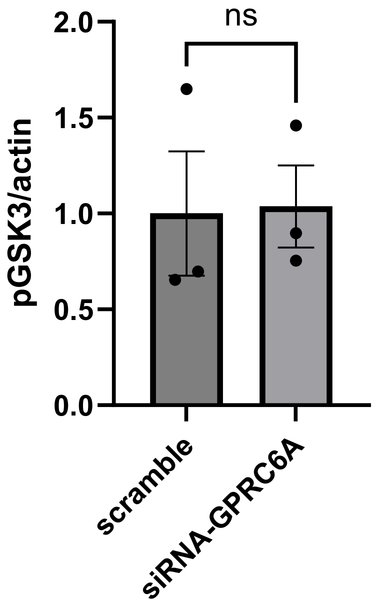

P-GSK3α+β
Rabbit 1:1000
Abcam ab4797
47Kd & 51Kd

B-actin
Mouse 1:50K
Sigma
42KD

One-way Anova, Fischer's LSD as post hoc analysis

Figure 9 Raw Blots

JCMC037e WB1 RAW

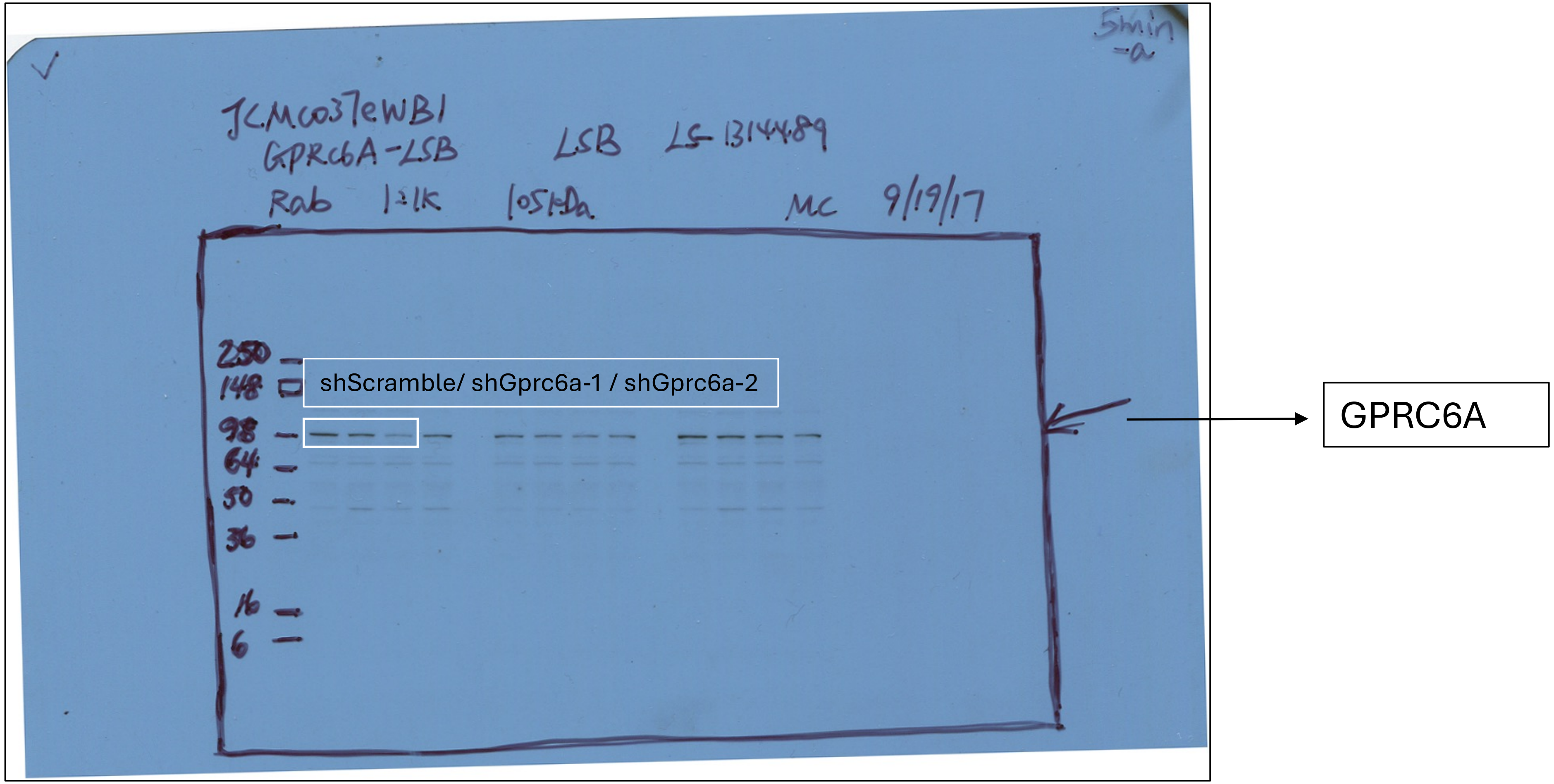

GPRC6A, Fig 9 A

JCMC037e WB1 RAW

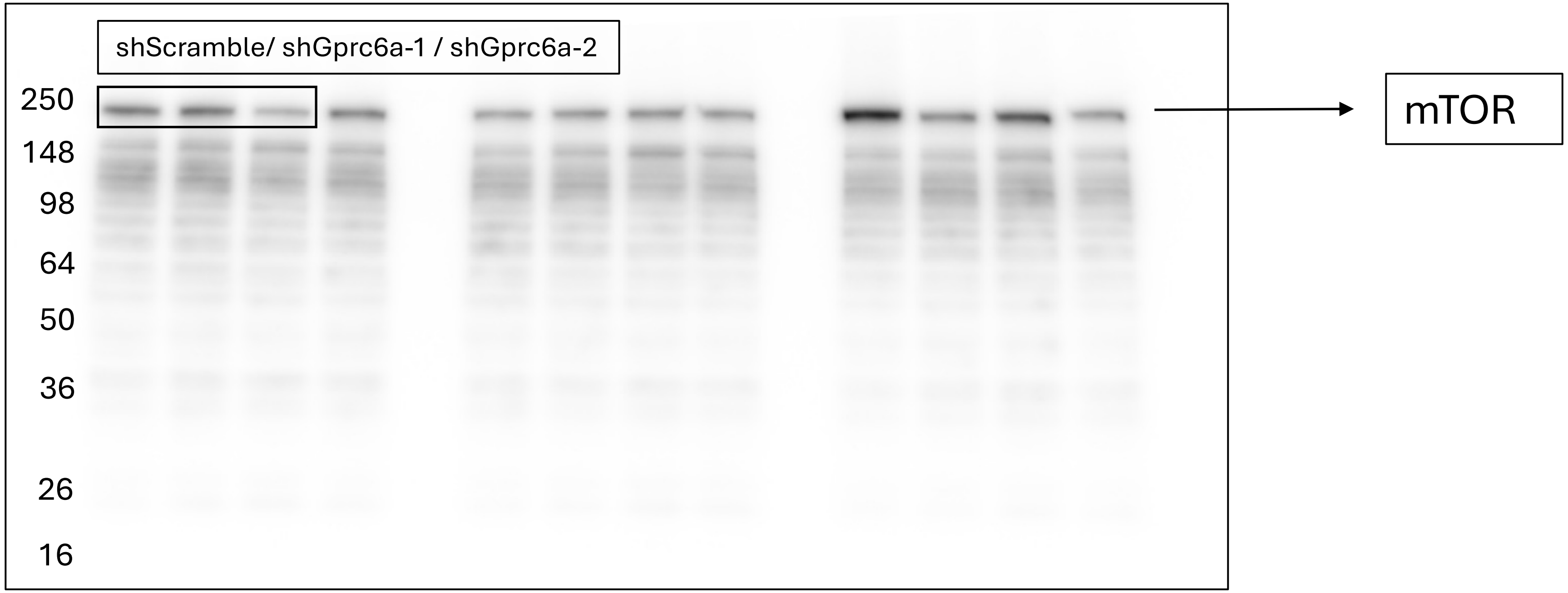

mTOR, Fig 9A

JCMC037e-WB2 RAW

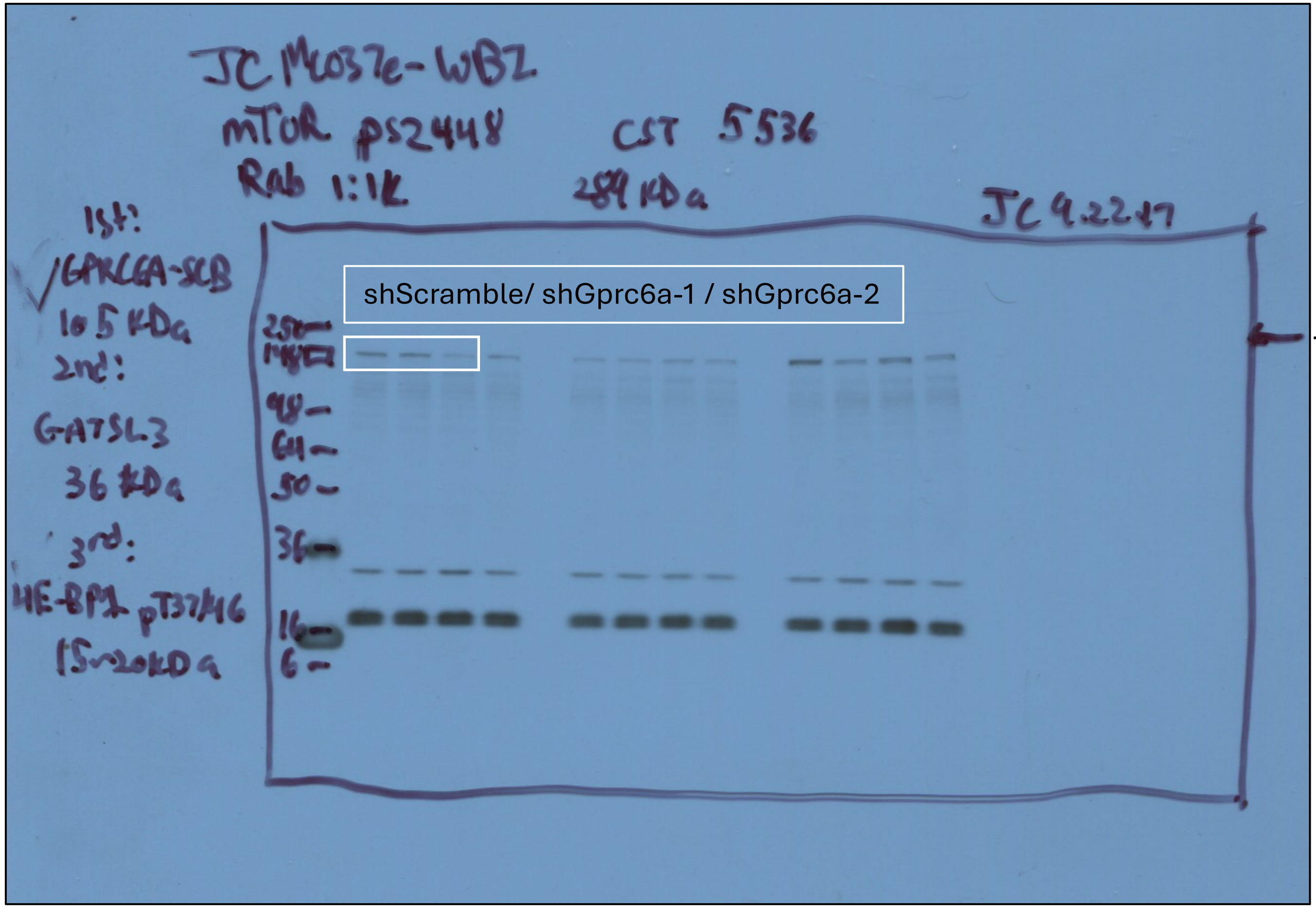

mTOR pS2448

mTOR pS2448, Fig 9 A

JCMC037e WB3 RAW

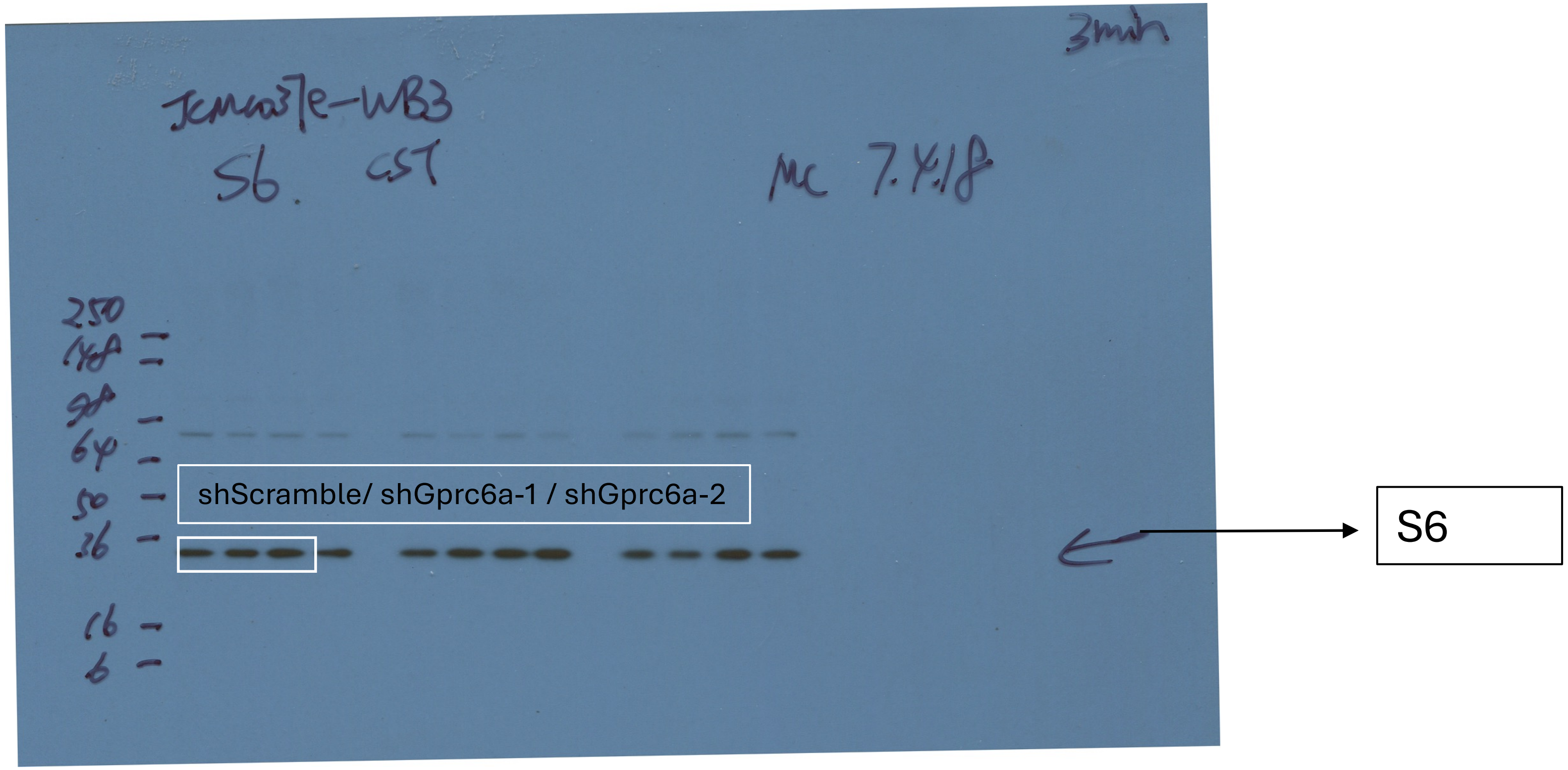

S6, Fig 9A

JCMC037e WB7 RAW

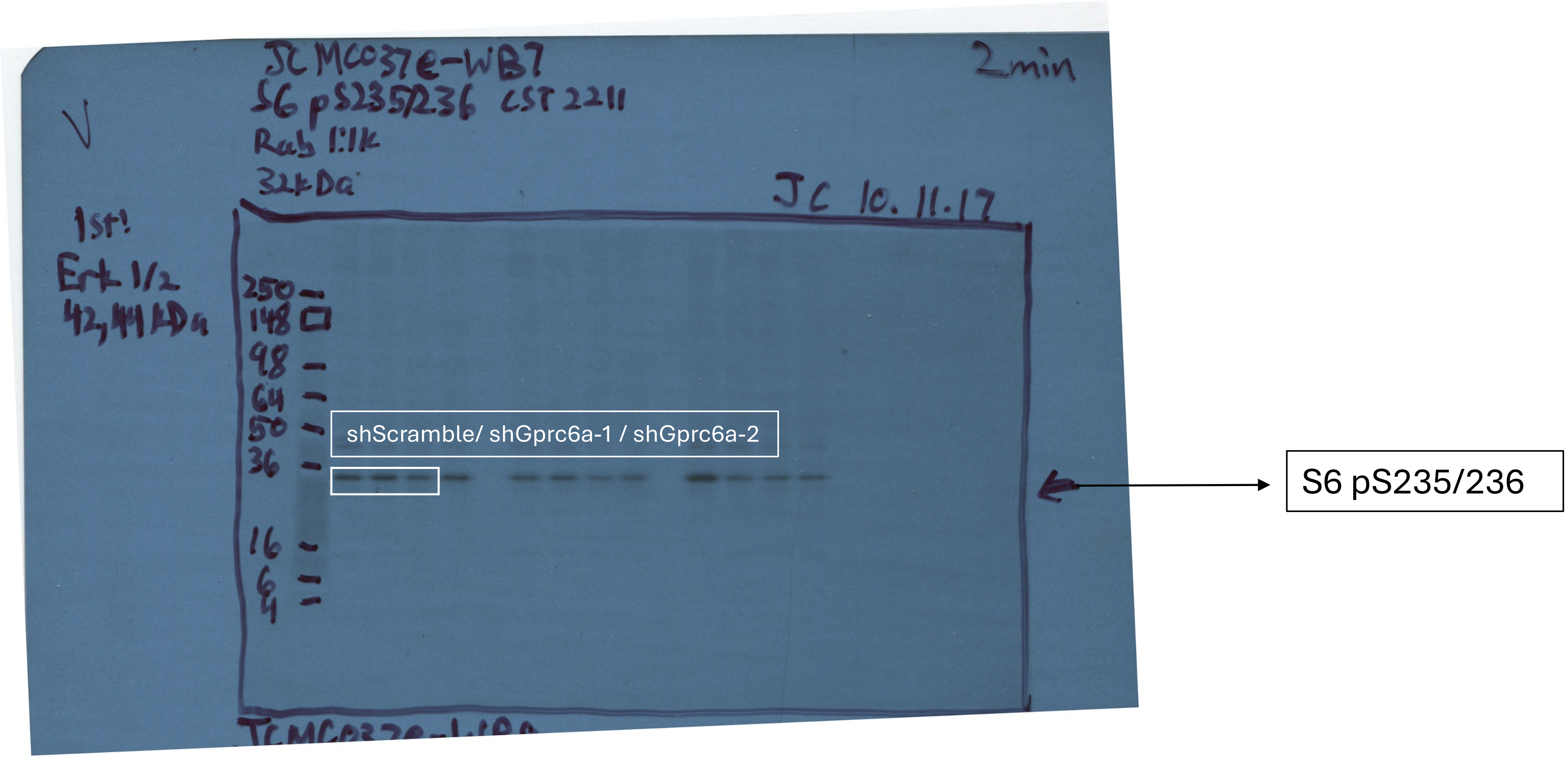

S6 pS235/236, Fig 9A

JCMC037e WB8 RAW

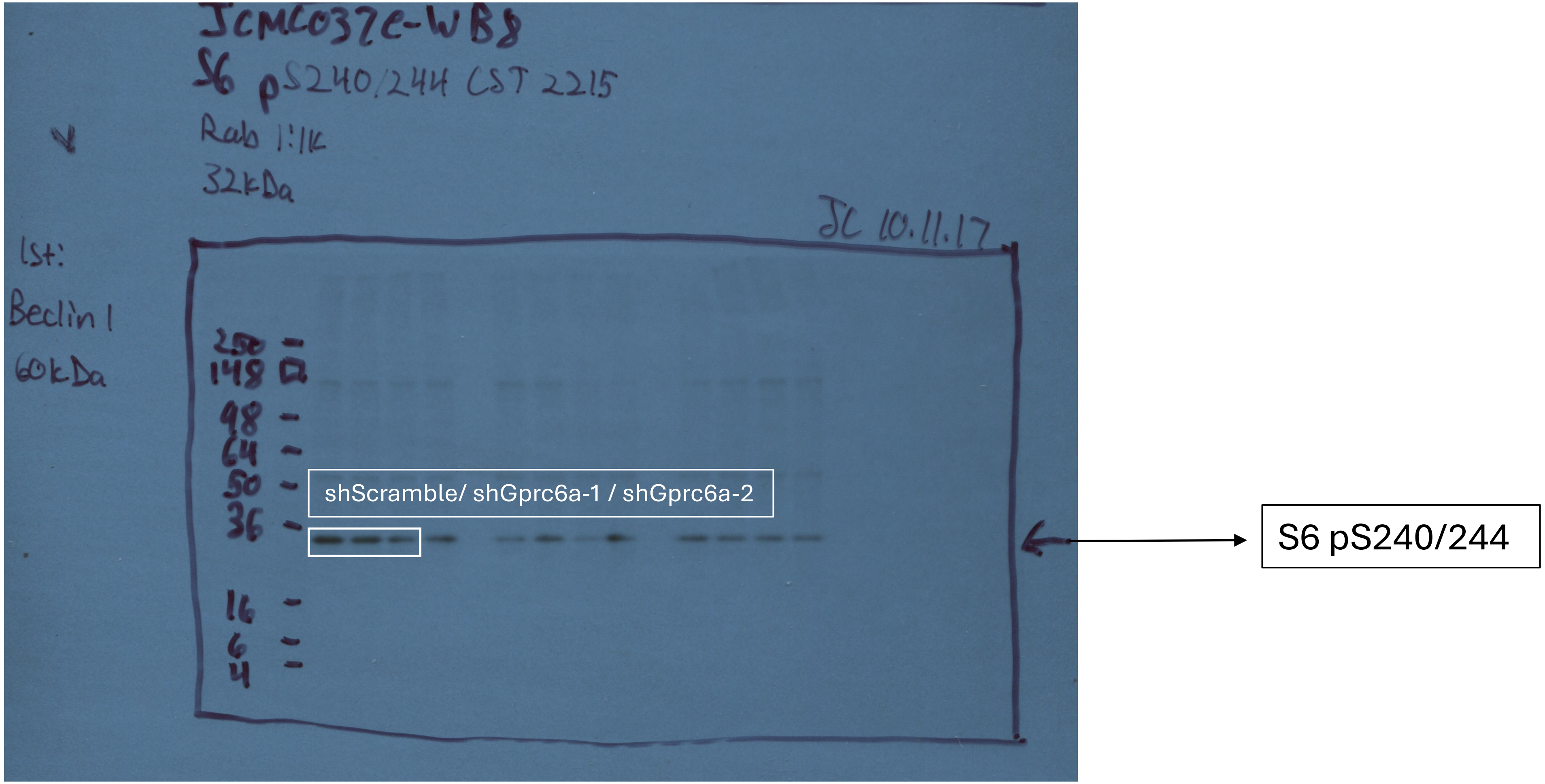

S6 pS240/244, Fig 9A

Fig 9 JCMC-037e-WB4

Added data for editor/reviewers but not included in the manuscript

Anti-GSK3 (alpha + beta) (phospho Y216 + Y279) antibody

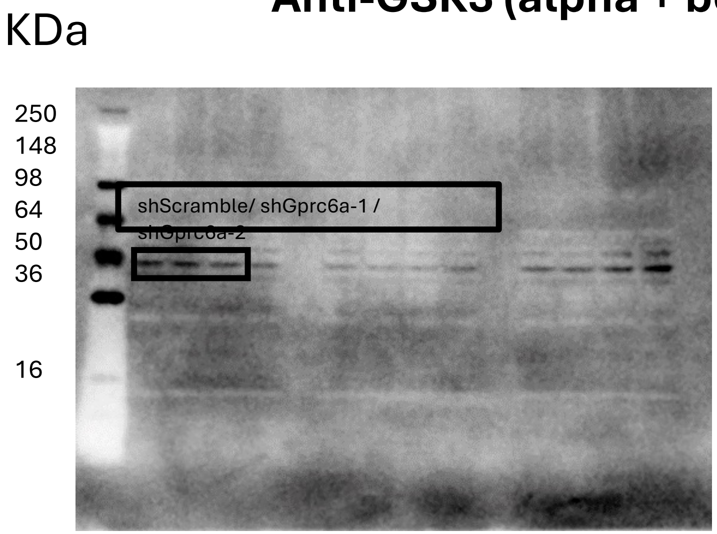

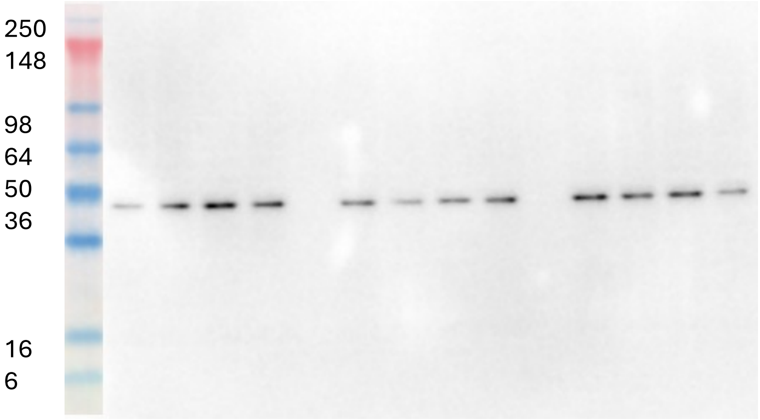

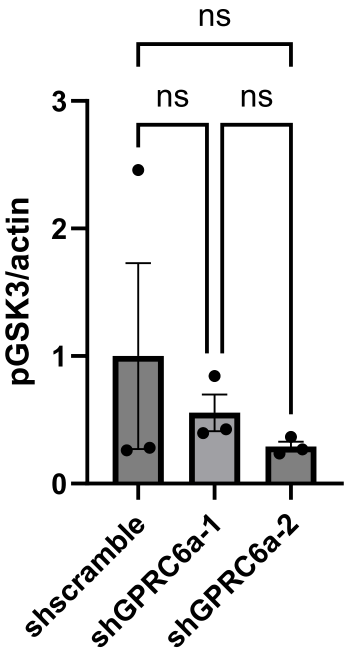

P-GSK3α+β
Rabbit 1:1000
Abcam ab4797
47Kd & 51Kd

B-actin
Mouse 1:50K
Sigma
42KD

One-way Anova, Fischer's LSD as post hoc analysis

G6AtGFP_S1_WB1

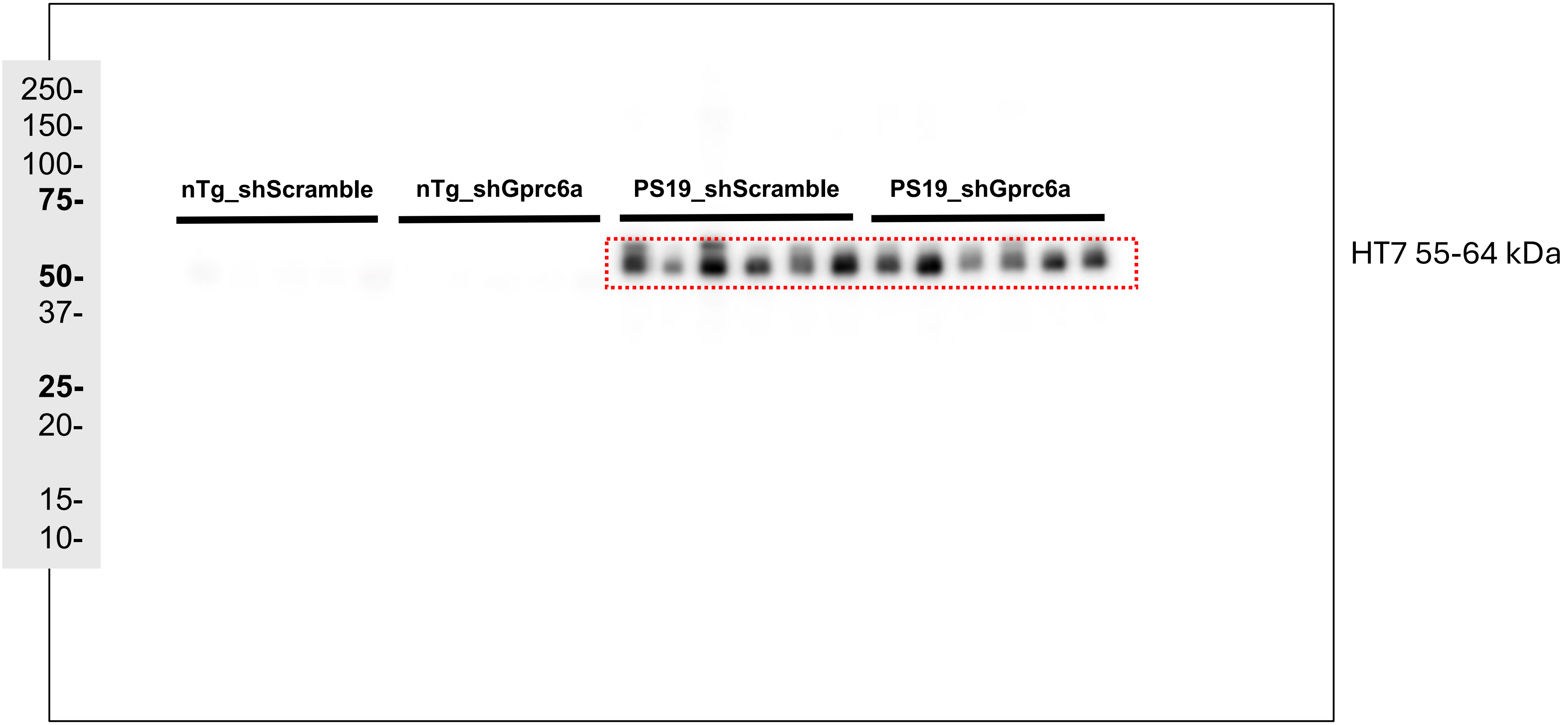

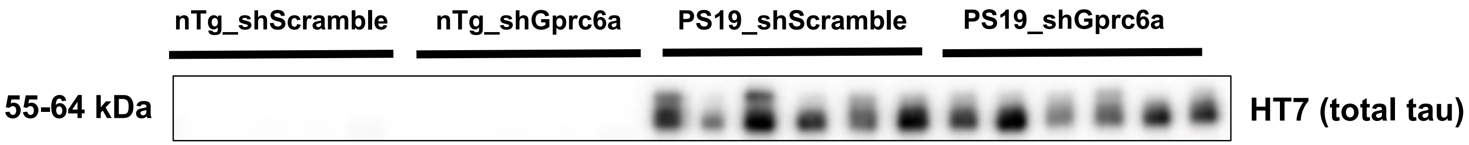

Soluble Tau (S1)

S1: Tau (total, HT-7) Fig 9F

G6AtGFP_S1_WB2

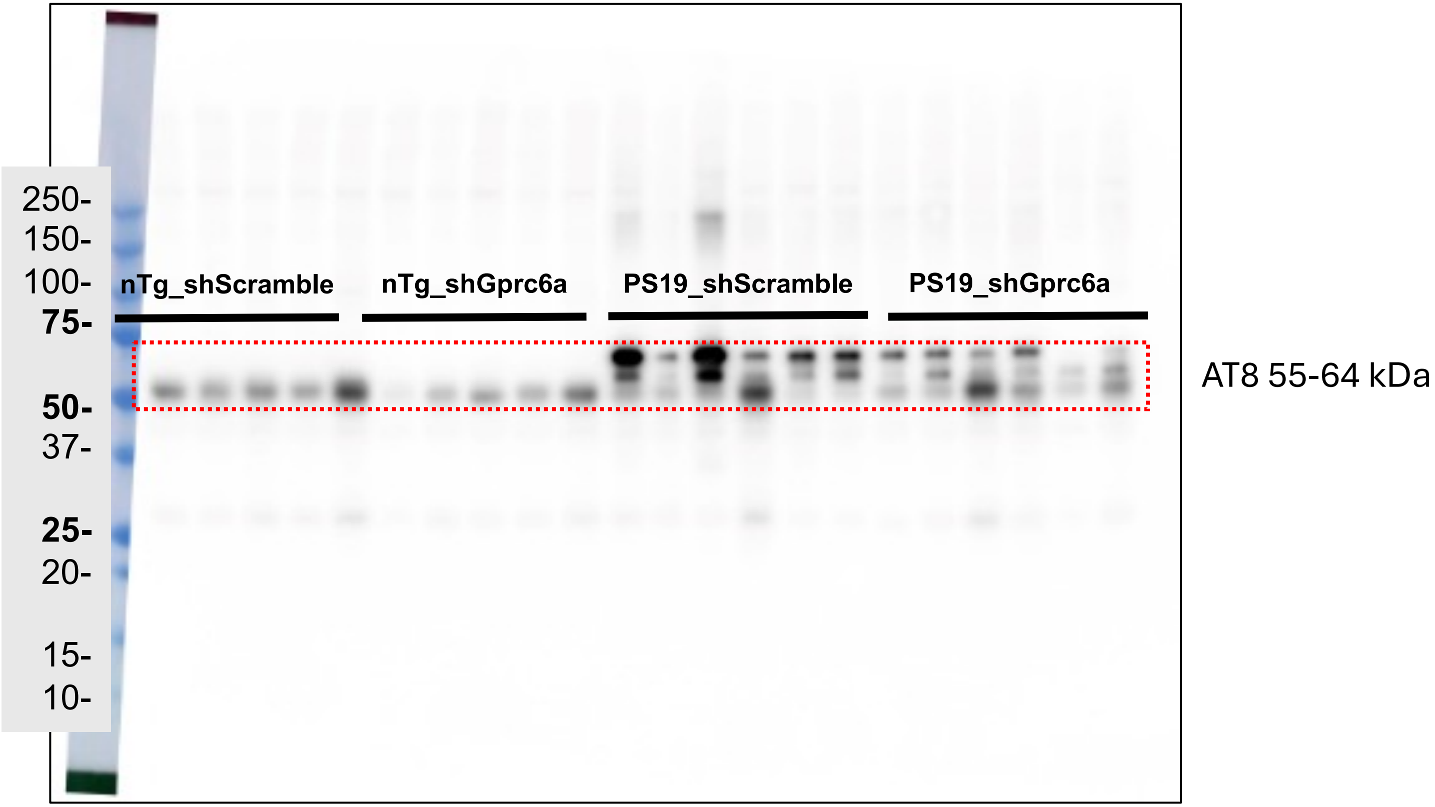

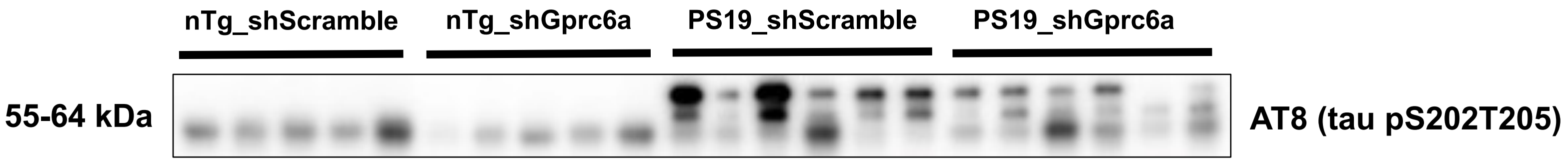

Soluble Tau (S1)

S1: Tau (AT8, pS202/T205) Fig 9F

G6AtGFP_S1_WB3

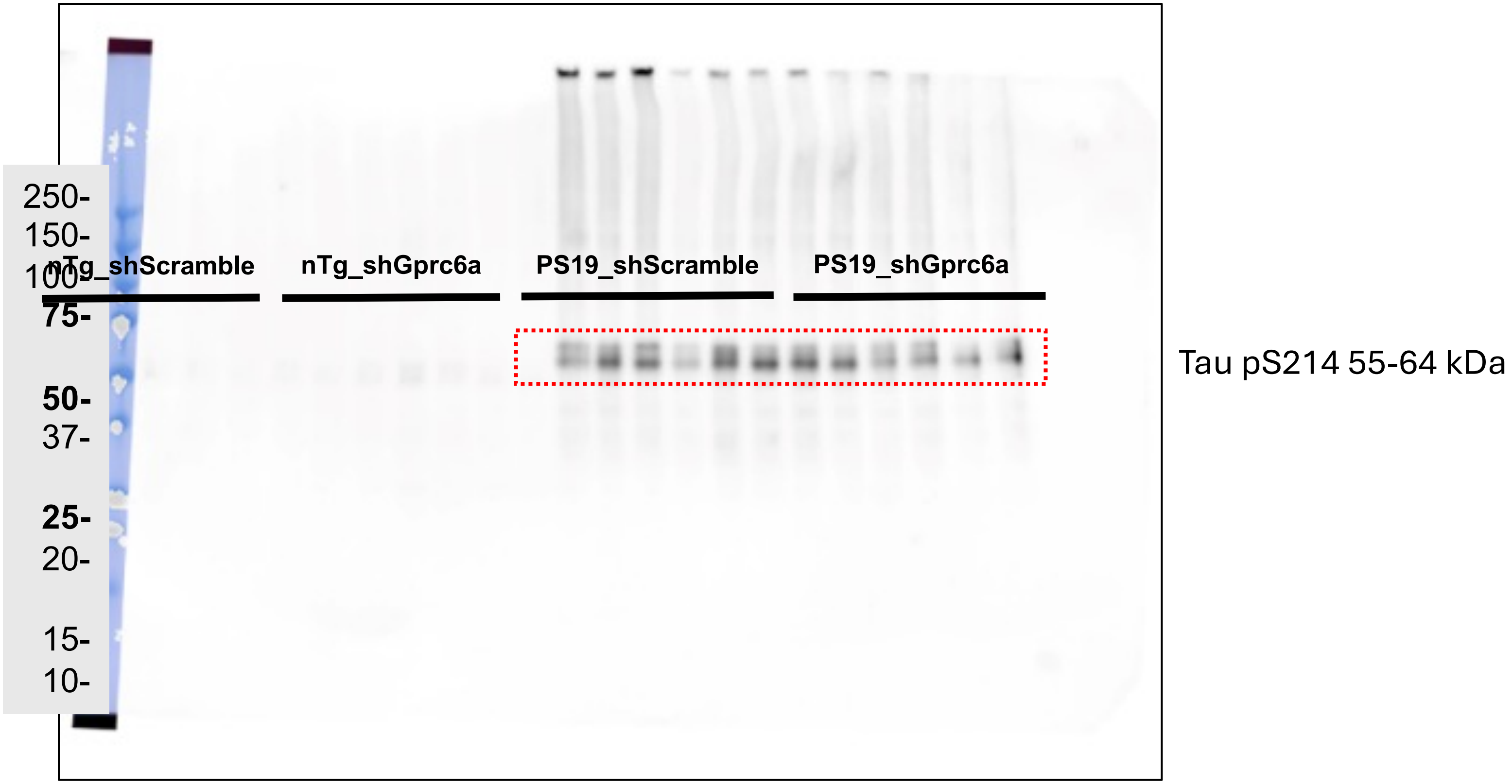

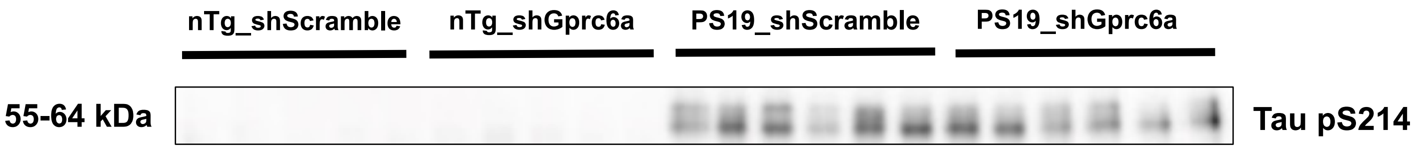

Soluble Tau (S1)

S1: Tau (pS214) Fig 9F

G6AtGFP_S2_WB3

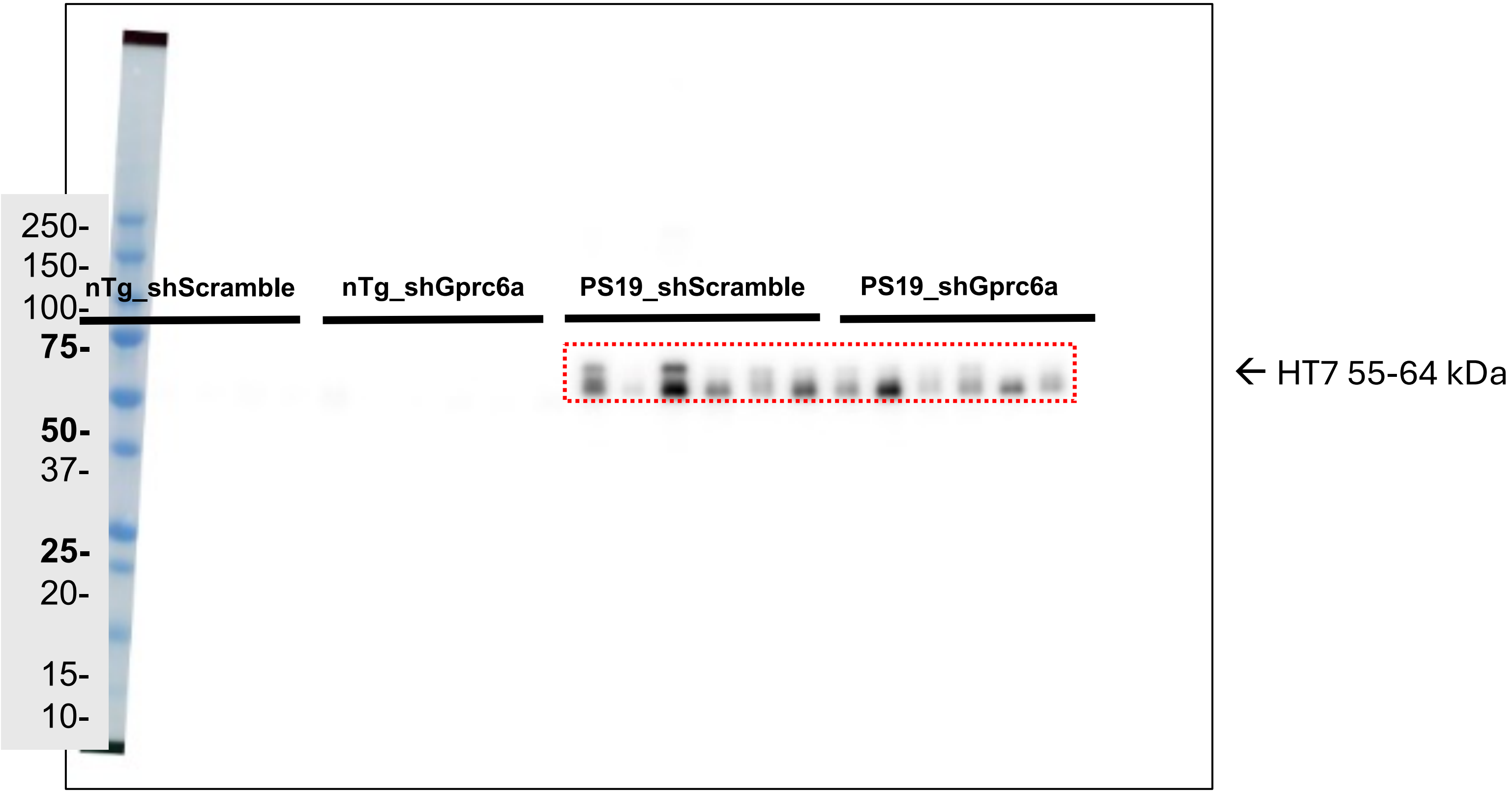

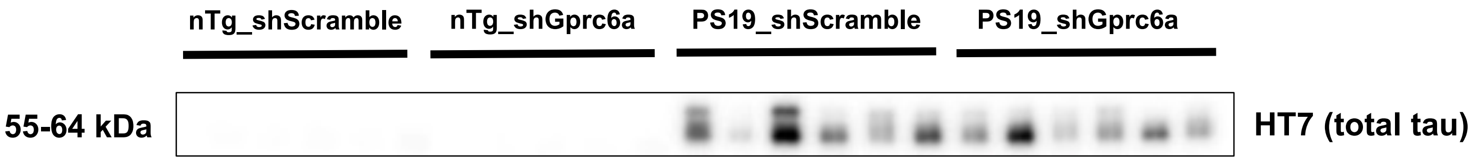

Sarkosyl Soluble Tau (S2)

S2: Tau (total, HT-7) Fig 9F

G6AtGFP_S2_WB1

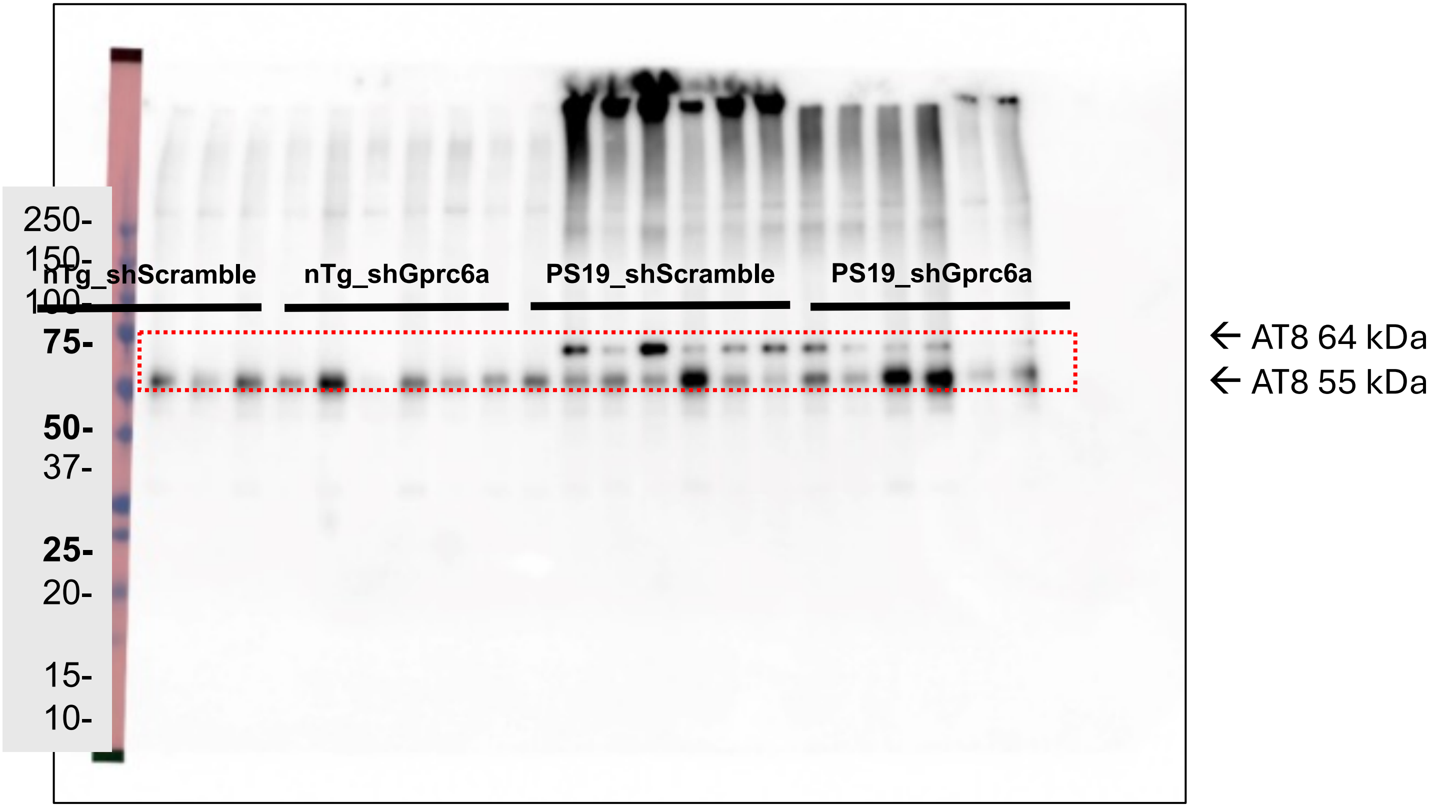

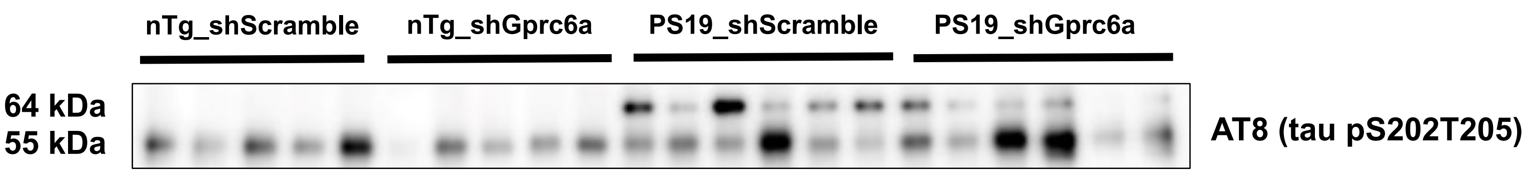

Sarkosyl Soluble Tau (S2)

S2: Tau (AT8, pS202/T205) Fig 9F

G6AtGFP_S2_WB2

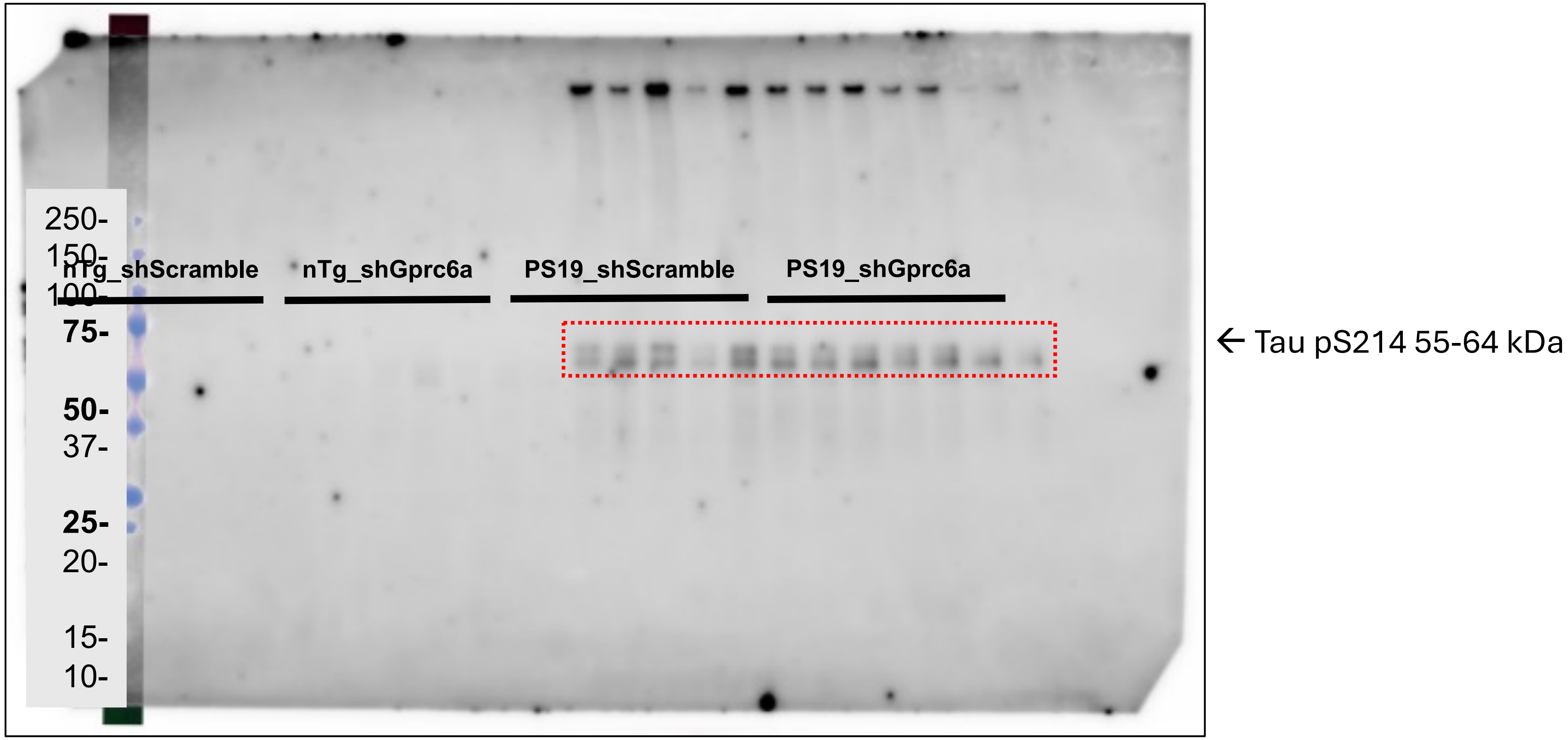

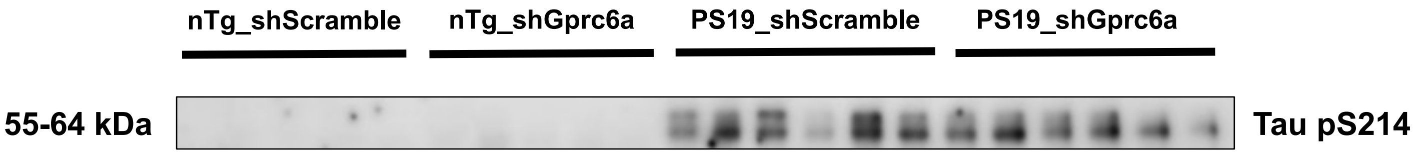

Sarkosyl Soluble Tau (S2)

S2: Tau (pS214) Fig 9F

G6AtGFP_P3_WB3

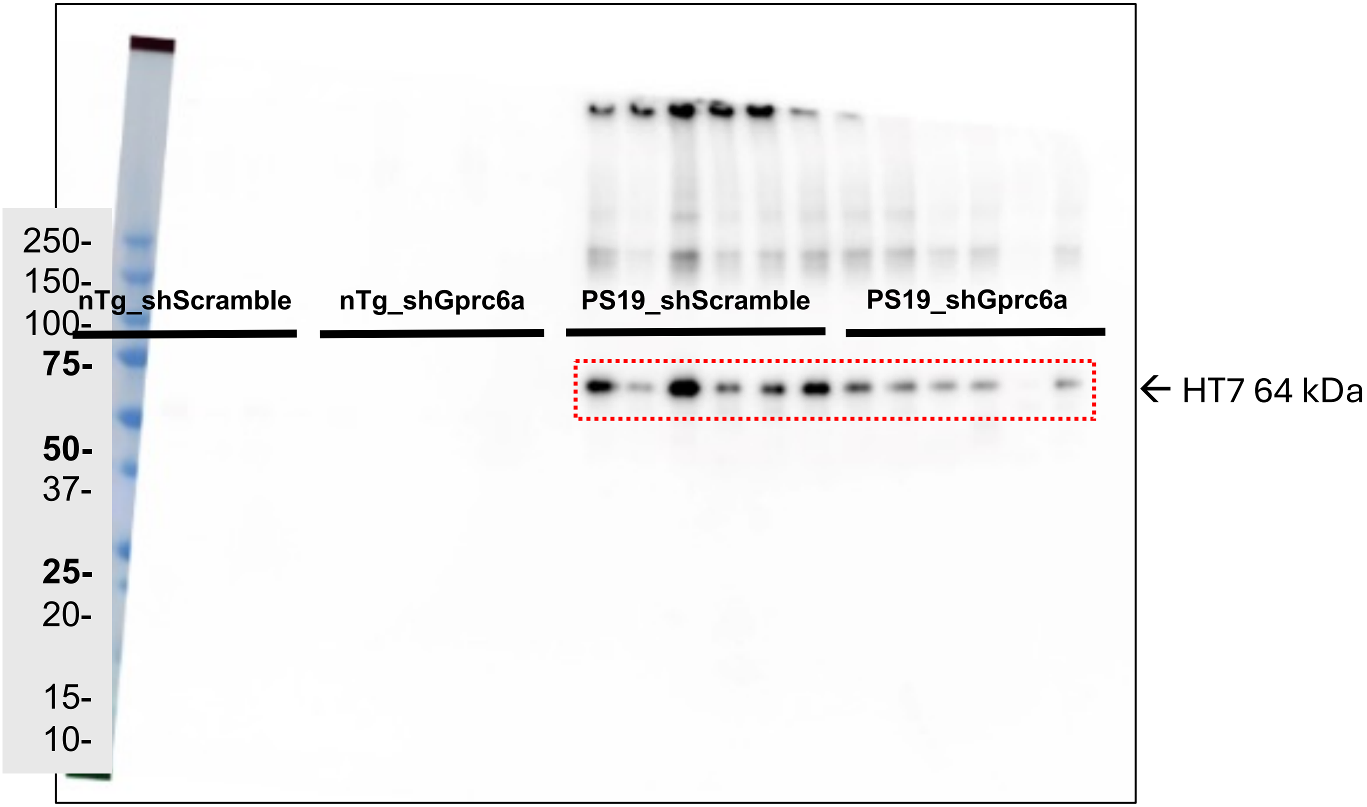

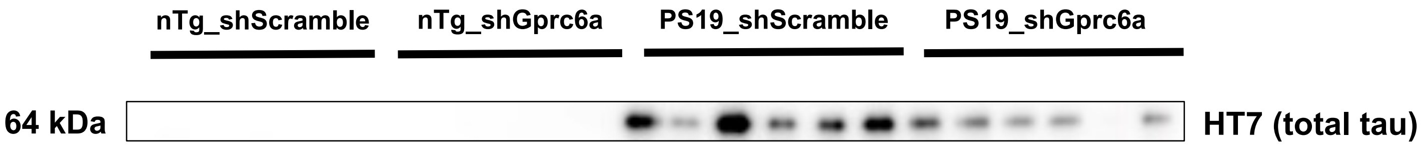

Sarkosyl Insoluble Tau (P3)

P3: Tau (total, HT-7) Fig 9F

G6AtGFP_P3_WB1

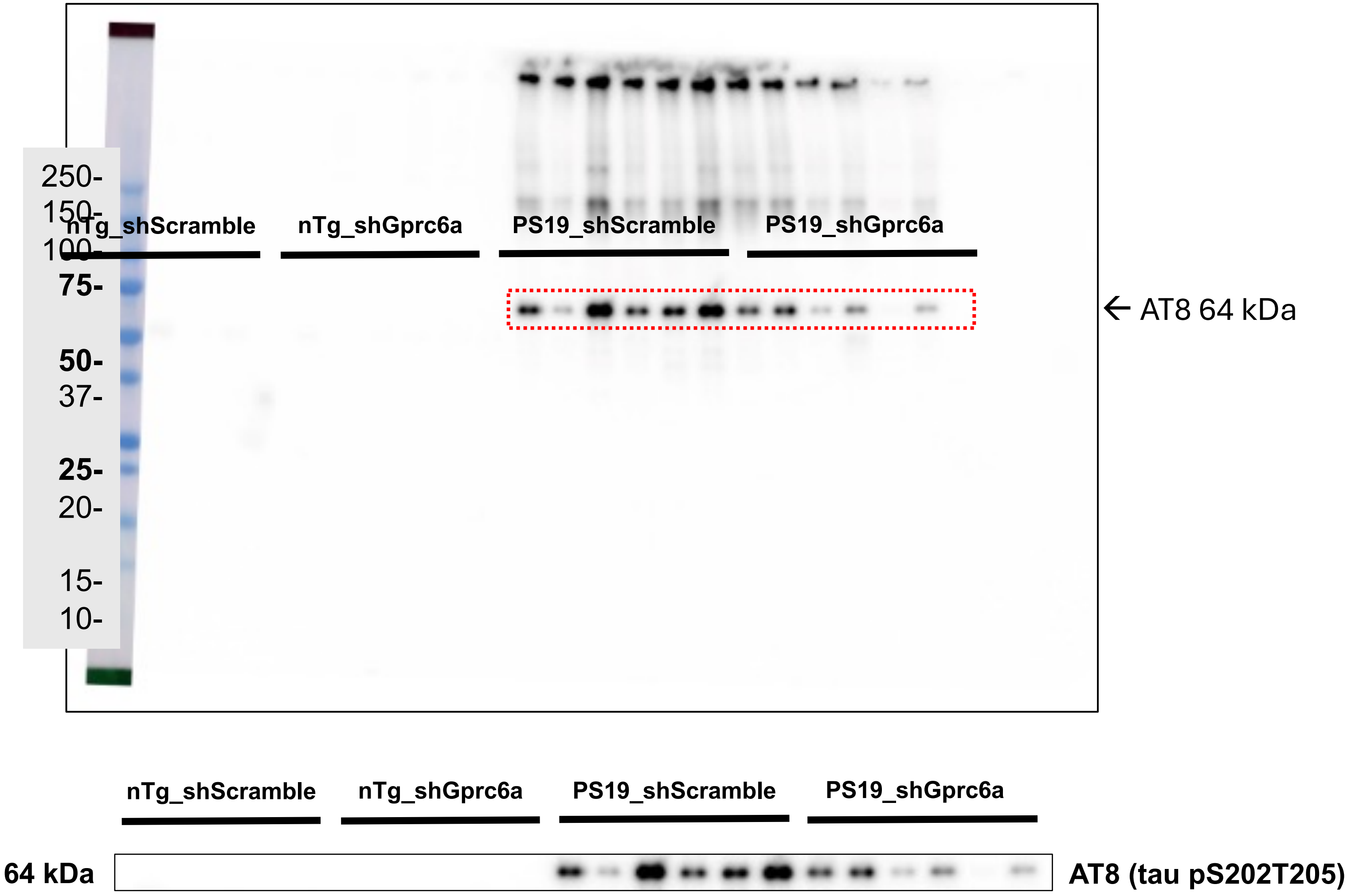

Sarkosyl Insoluble Tau (P3)

P3: Tau (AT8, pS202/T205) Fig 9F

G6AtGFP_P3_WB2

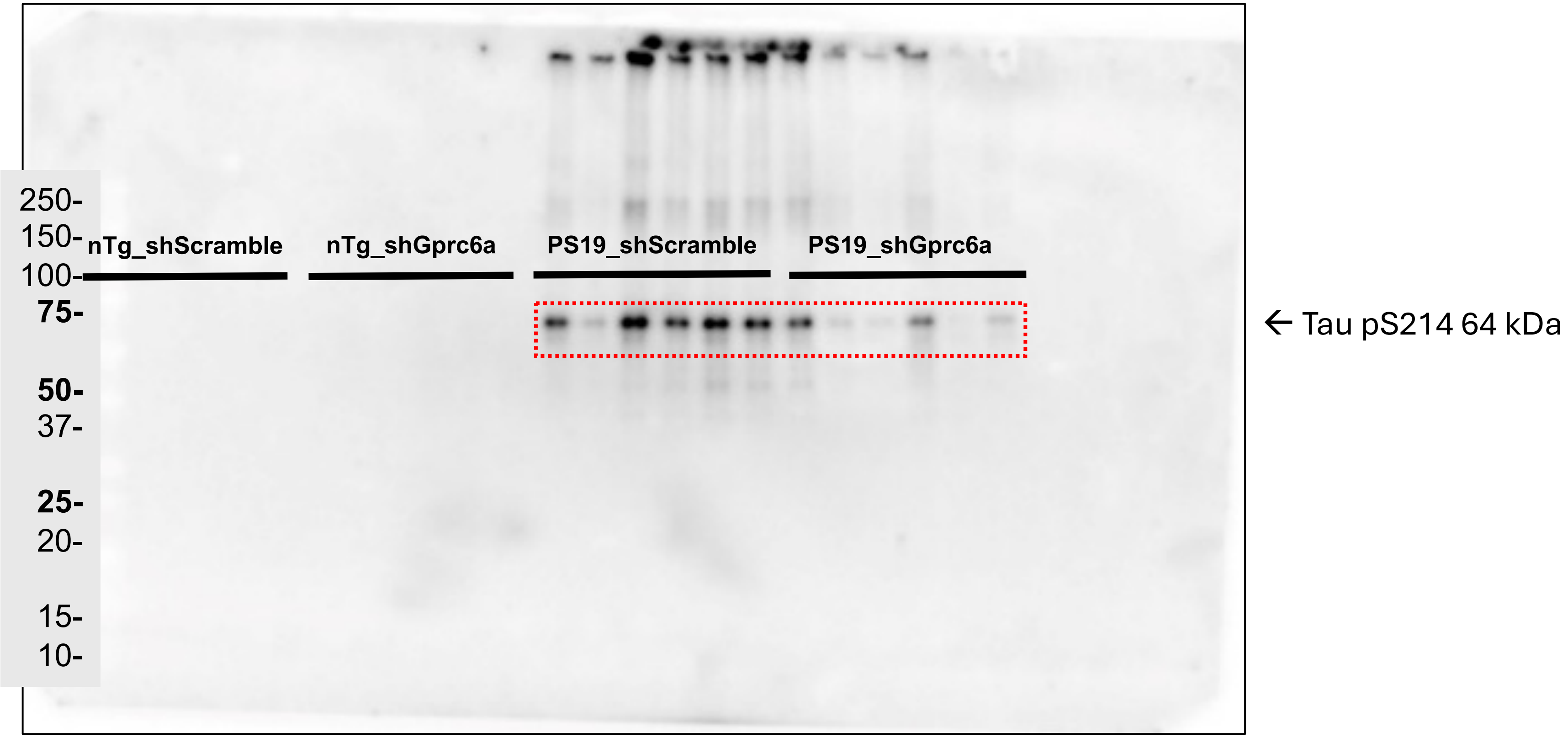

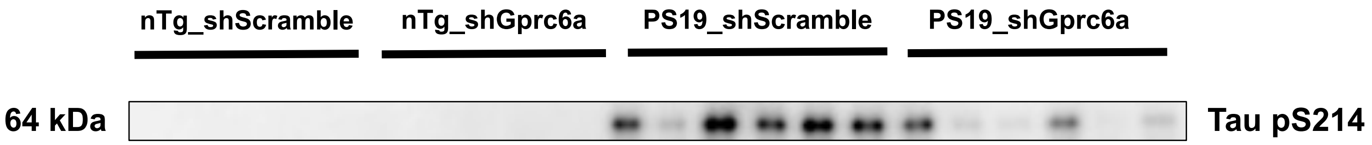

Sarkosyl Insoluble Tau (P3)

P3: Tau (pS214) Fig 9F

Additional Data requested by reviewers for Figure 10.

Overexposed

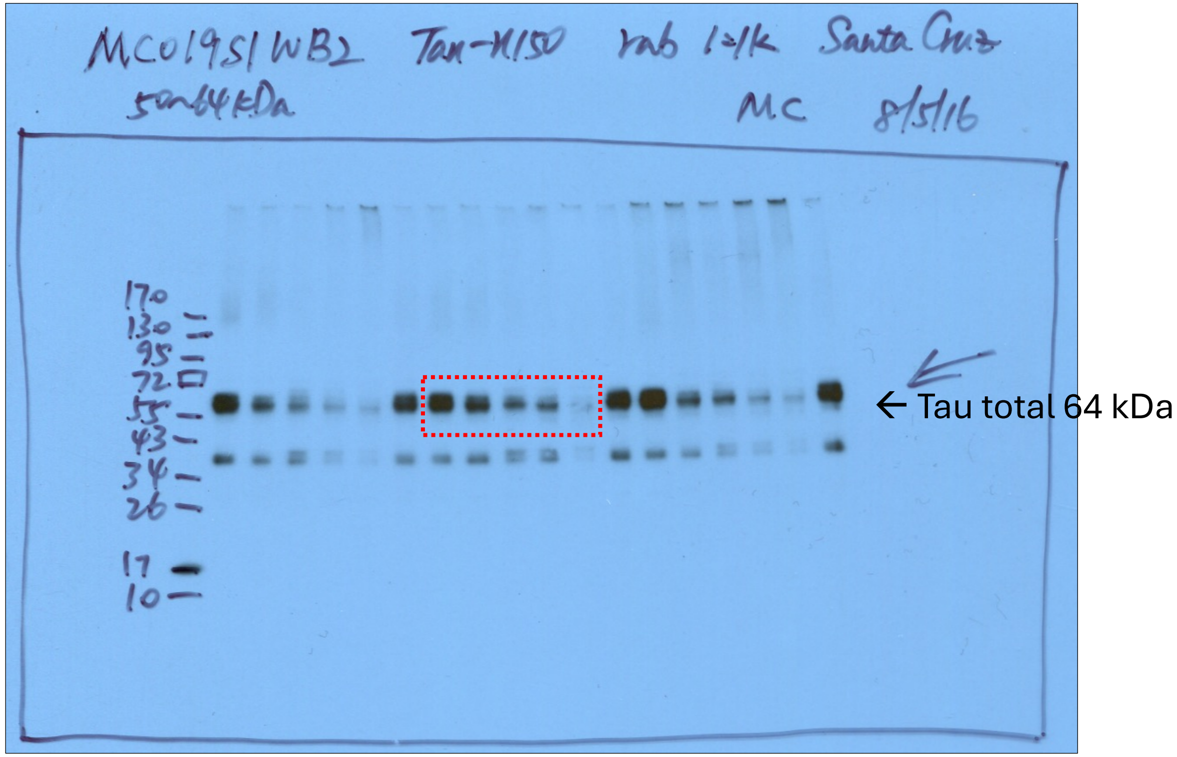

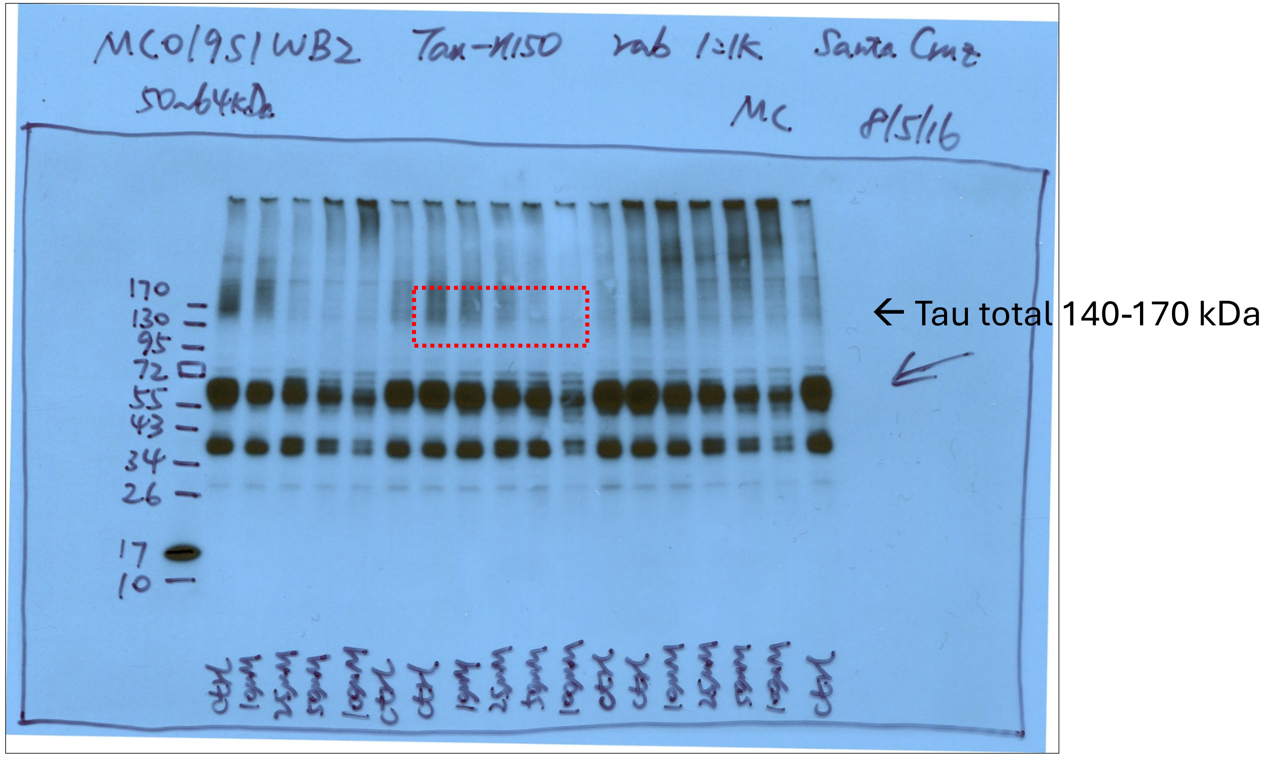

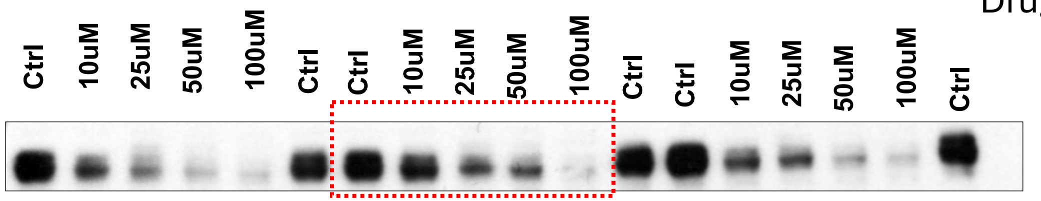

Drug #47661

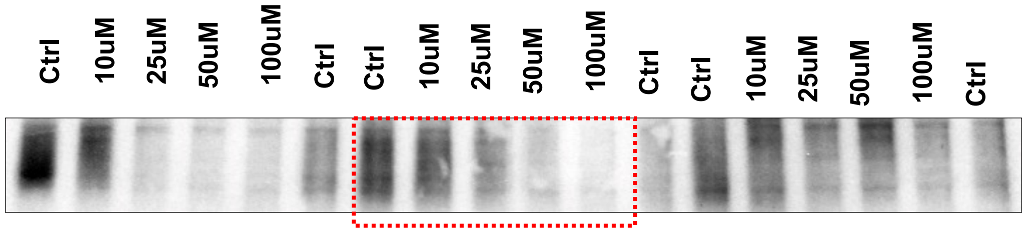

Tau (total) Fig 10G, H, I
